# Sandmeyer Chlorosulfonylation of (Hetero)Aromatic Amines Using DABSO as an SO_2_ Surrogate

**DOI:** 10.1021/acs.orglett.4c01908

**Published:** 2024-07-11

**Authors:** Lucia Pincekova, Aurélien Merot, Gabriel Schäfer, Michael C. Willis

**Affiliations:** † Department of Chemistry, University of Oxford, Chemistry Research Laboratory, Mansfield Road, Oxford OX1 3TA, U.K.; ‡ Chemistry Process R&D, Idorsia Pharmaceuticals Ltd., Hegenheimermattweg 91, CH-4123 Allschwil, Switzerland

## Abstract

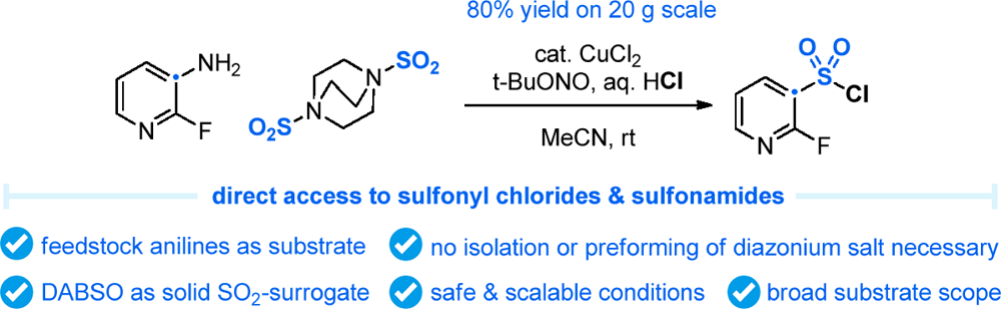

Sulfonyl chlorides not only play a crucial role in protecting group chemistry but also are important starting materials in the synthesis of sulfonamides, which are in-demand motifs in drug discovery chemistry. Despite their importance, the number of different synthetic approaches to sulfonyl chlorides is limited, and most of them rely on traditional oxidative chlorination chemistry from thiol precursors. In this report, we disclose a novel Sandmeyer-type sulfonyl chloride synthesis from feedstock anilines and DABSO, used as a stable SO_2_ surrogate, in the presence of HCl and a Cu catalyst. The method works on a wide range of anilines and allows for the isolation of the sulfonyl chloride after aqueous workup or its direct conversion into the sulfonamide by simple addition of an amine after the completion of the Sandmeyer reaction. The scalability of this method was demonstrated on a 20 g scale, and the corresponding heterocyclic sulfonyl chloride was isolated in 80% yield and excellent purity.

The importance of modern organosulfur chemistry cannot be overstated, as organosulfur compounds find widespread applications in agrochemicals,^1^ materials,^2^ fine chemicals, and pharmaceuticals.^3^ Of the many different sulfur-containing functional groups, sulfonamides have a special place in modern drug discovery, as they can act as bioisosteres for carboxylic acids, esters, and amides.^4^ Sulfonamides offer several advantages, such as an additional hydrogen bond acceptor, improved hydrolytic stability, and an increased polar surface area.^5^ Despite the ubiquity of sulfonamides, their synthesis relies nearly exclusively on one textbook transformation, the reaction of an amine with a sulfonyl chloride in the presence of a base.^6^ While this method works reliably and normally affords a high yield, it has an innate flaw: the use of sulfonyl chlorides. While standard sulfonyl chlorides are commercially available, more complex examples, including many heterocyclic variants, are not commercially available and need to be prepared. Unfortunately, their preparations and isolations are far from trivial due to their propensity for hydrolysis. This issue is aggravated by most preparations relying on the use of aqueous oxidants (for example, NaClO,^7^ aqueous H_2_O_2_,^8^ or aqueous oxone^9^) or aqueous halide sources (for example, aqueous NCS or aqueous HCl).^10^ The combination of both an aqueous oxidant and HCl is especially problematic, as it will lead to the release of chlorine gas. We have recently replaced such an oxidative chlorination process in the synthesis of a sulfonamide by selective oxidation of a thiol precursor to a sulfinate salt, followed by formation of the sulfonamide with hydroxylamine-*O*-sulfonic acid (HOSA) as the electrophilic amination reagent.^11^

To address the shortcomings of sulfonyl chloride synthesis, we envisioned a method that relied on neither oxidative chlorination chemistry nor the use of oxidation-sensitive thiol precursors. To this end, we have recently reported the synthesis of sulfonyl chlorides from Grignard reagents and DABCO-bis(sulfur dioxide) (DABSO),^12^ with the DABSO reagent used as an SO_2_ surrogate.^13^ DABSO is an inexpensive, bench stable, easily handled crystalline solid, which is commercially available from multiple suppliers.^14^ To expand the DABSO methodology, we targeted the use of more widely available starting materials and were inspired by a seminal publication of Hogan and Cox on the Sandmeyer-type synthesis of sulfonyl chlorides from anilines (Scheme 1).^15^ Anilines are some of the cheapest, most widely available feedstocks in organic chemistry. While the original procedure from Hogan and Cox worked well for a handful of substrates, it was for several reasons not ideal from a process chemistry perspective. (1) The highly energetic diazonium intermediate needed to be preformed in a separate pot by using a strongly acidic environment. This presents a severe safety risk for production, as especially heteroaromatic diazonium salts are highly unstable and can violently decompose under the release of N_2_ gas. (2) SO_2_ was generated by adding neat SOCl_2_ (4.3 equiv) to water, which is a highly exothermic reaction. After addition, the aqueous mixture needed then to be slowly warmed to room temperature (rt) over the course of 17 h. (3) The preformed aqueous diazonium slurry was then added slowly (while being kept cold at −5 °C) to the aqueous sulfur dioxide solution containing catalytic CuCl. (4) The reaction only worked well for sulfonyl chlorides that were directly crystallized from the aqueous reaction mixture and therefore protected from hydrolysis. For soluble or noncrystalline sulfonyl chlorides, the highly acidic aqueous reaction conditions will lead to their hydrolysis.^16^ In addition, an *in situ* quenching of the sulfonyl chloride with an amine will not be possible due to the large excess of 36% aqueous HCl (12 equiv) used in the diazonium formation step. We wanted to address these shortcomings by using DABSO as a bench stable SO_2_ surrogate and develop a simple one-pot process that can be applied to a wide range of anilines and allowed for facile isolation of the sulfonyl chloride or sulfonamide after quenching with an amine. Crucially, although DABSO has been used in combination with aryl diazonium salts, with the salts being both isolated and generated *in situ*, toward a variety of sulfonyl-containing targets, it has not been used directly with anilines.^17^ The synthesis of sulfonyl chlorides from the combination of aryl diazonium salts and DABSO is also unknown.^18^

**Scheme 1:**
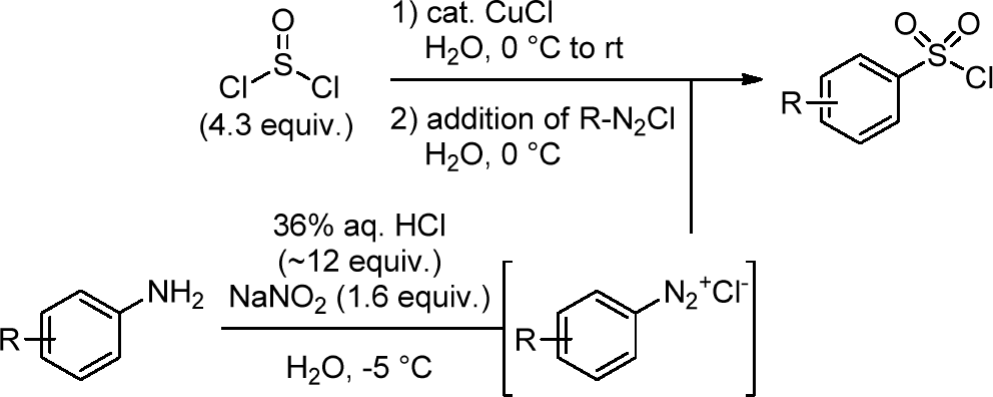
Sandmeyer Chlorosulfonylation by Hogan and Cox Using Preformed Diazonium Salts^15^

At the outset of the project, we selected 2-fluoropyridin-3-amine (**2**) as model substrate, as the corresponding sulfonyl chloride **1** was needed on a multigram scale for structure–activity relationship studies at Idorsia Pharmaceuticals Ltd. Our idea was to simply combine aniline **2**, DABSO, and a Cu source in a mixture of HCl (2.0 equiv) and MeCN and then add *tert*-butyl nitrite (1.1 equiv) in a controlled manner (Table 1). After some preliminary screening, we found a promising initial hit by using a combination of 32% aqueous HCl and catalytic CuCl (entry 1). The addition of *tert*-butyl nitrite was found to be dose-controlled, and full conversion of aniline **2** was reached after only 15 min at rt. Changing the Cu source to CuCl_2_ had a beneficial effect on the overall purity of the reaction (entry 2), even if only 0.025 equiv of the Cu catalyst was used (entry 3). Reducing the amount of 32% aqueous HCl (entry 4) or adding more diluted HCl solutions (entries 5 and 6) still led to full conversion of the amine, but with a negative effect on the overall purity of the reaction. The largest impurity formed in these reactions (entries 1–6) was 3-chloro-2-fluoropyridine stemming from a Sandmeyer chlorination process.^19^ Replacing aqueous HCl with 5–6 M HCl in *i*-PrOH was not possible, as full conversion of **2** was not reached and several side products were formed (entry 7).

**Table 1:**
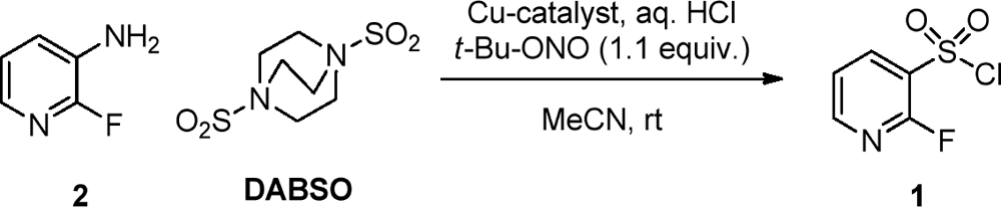
Optimization of the DABSO Sandmeyer Procedure^a^

entry	HCl source (equiv)	Cu catalyst (equiv)	conversion (%), overall purity (%)^b^
1	32% aqueous HCl (2.0)	CuCl (0.1)	>99, 74 a/a
2	32% aqueous HCl (2.0)	CuCl_2_ (0.1)	>99, 82 a/a
3	32% aqueous HCl (2.0)	CuCl_2_ (0.025)	>99, 82 a/a
4	32% aqueous HCl (1.0)	CuCl_2_ (0.025)	>99, 71 a/a
5	25% aqueous HCl (2.0)	CuCl_2_ (0.025)	>99, 74 a/a
6	2 M aqueous HCl (2.0)	CuCl_2_ (0.025)	>99, 64 a/a
7	HCl in *i*-PrOH (2.0)	CuCl_2_ (0.025)	75, 40 a/a

a Reaction conditions: **2** (1.0 g, 8.92 mmol, 1.0 equiv), DABSO (1.31 g, 5.35 mmol, 0.60 equiv), and a Cu catalyst in MeCN (10 mL) and HCl, then dropwise addition of 90% *tert*-butyl nitrite (1.3 mL, 9.81 mmol, 1.1 equiv) over 15 min at rt. Sampling was performed 1 h after the addition.

b Conversion was judged by the consumption of **2** relative to the formation of **1** by LC/MS at 210 nm. The overall purity refers to the area/area (a/a) percentage of **1** after 1 h as determined by LC/MS at 210 nm.

With the optimized conditions in hand, we continued with process safety investigations. The reaction was performed in a calorimeter (RC-1) on a 12 g scale, adding *tert*-butyl nitrite over 30 min at 20 °C (see the Supporting Information for details). The measurement confirmed the addition to be fully dose-controlled without any thermal accumulation. By taking samples during the *tert*-butyl nitrite addition and analyzing them by LC/MS, we confirmed the absence of the diazonium intermediate, which showed that no significant amount of this highly energetic species was ever built up during the reaction. This makes our protocol inherently safe and straightforward compared to that reported by Hogan and Cox,^15^ as no preformation of the diazonium salt is needed, which is a crucial improvement with regard to process safety and scale up. With this information in hand, we performed the reaction on a 20 g scale (Scheme 2). After full conversion of aniline **2**, CPME was added, followed by quenching with aqueous sulfamic acid. After being washed with H_2_O, the organic phase was concentrated to dryness, and the residue was purified by Kugelrohr distillation. The desired sulfonyl chloride was isolated as an orange oil in 80% yield with good purity [95% (w/w) as determined by the ^1^H NMR assay].

**Scheme 2:**
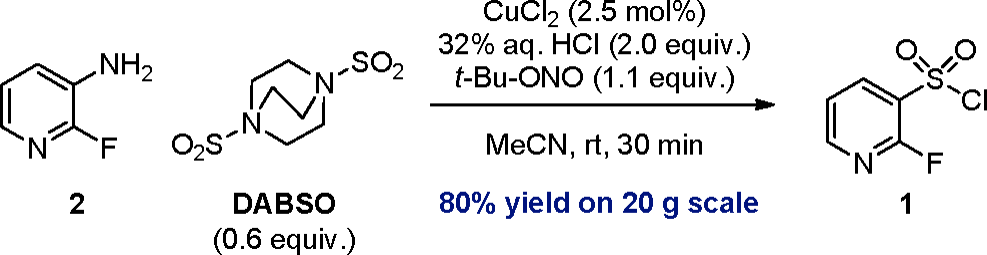
DABSO Sandmeyer Reaction for the Preparation of Sulfonyl Chloride **1** on a 20 g Scale

We were curious to test the generality of the optimized DABSO Sandmeyer conditions and therefore explored the substrate scope. Given the increased stability and ease of isolation of sulfonamides, we decided to convert the generated sulfonyl chlorides *in situ* into their corresponding sulfonamides by the addition of morpholine. For our scope study, we reduced the scale of the reactions from those used in the initial optimization study and routinely performed experiments on a 0.5 mmol scale of amine. While we believe these reactions also to be dose-controlled, we allowed the mixtures to stir overnight after the completed *tert*-butyl nitrite addition and added the morpholine at the start of the next day, as this allowed us to set up multiple substrates simultaneously. We were pleased to find that the reaction translated well to a series of carbocyclic anilines featuring electron-withdrawing substituents, with the corresponding morpholine-derived sulfonamides isolated in good yields (Scheme 3, **3a**–**3o**). A variety of functionalities, including halogen (**3a**–**3c**, **3i**, **3j**, **3l**, and **3n**), trifluoromethyl (**3d**, **3k**, and **3m**), trifluoromethoxy (**3e**), nitrile (**3f**), nitro (**3g**), and ester (**3h**) groups, were all well tolerated, and products featuring groups placed at the *ortho*, *meta*, and *para* positions were accessible. Many of these products could be further functionalized by well-established protocols, such as cross-coupling reactions (e.g., **3b** and **3c**), reductions (e.g., **3f**–**3h**), or S_N_Ar (e.g., **3n**), which opens the door for a rapid structural diversification from a simple aniline precursor. When 2-aminobenzamide was used as a substrate, 1*H*-1λ^6^,2-benzothiazole-1,1,3(2*H*)-trione (**3o**), also known as Saccharin, was isolated as the sole product in 59% yield. To highlight the scalability of direct sulfonamide formation, *p*-chloro derivative **3b** was prepared on a 5 mmol scale with only a minimal reduction in yield. Such a direct conversion into the sulfonamide would be challenging with existing methods for sulfonyl chloride synthesis due to the strongly oxidative or acidic reaction conditions. Electron-neutral anilines also proved to be competent substates, and the corresponding products **3p**–**3t** were isolated in good yields. On the contrary, anilines bearing electron-donating substituents turned out to be more challenging substrates, as full conversion of the aniline could not be achieved at room temperature. Therefore, we decided to increase the reaction temperature to 75 °C to ensure full conversion to the corresponding sulfonyl chlorides. This is a clear indication that for these electron-rich substrates, the corresponding diazonium salts accumulated at room temperature. While for small scale reactions (0.5 mmol scale) this is not an issue, it would be an inherent safety risk for large scale reactions. Therefore, for larger scale reactions, we recommend trying to dose the *tert*-butyl nitrite directly at 75 °C to avoid accumulation of highly energetic diazonium intermediates. With this slight modification in place, products featuring a variety of electron-donating substituents at different ring positions were successfully synthesized (**3u**–**3z**). The high isolated yield of **3z** highlights the mildness of our reaction conditions, as this product would be extremely difficult to synthesize using the traditional oxidative chlorination approach due to the competing oxidation of the thioether moiety. We also revalidated the possibility of isolating the sulfonyl chloride intermediate, as shown by example **3u′**, which was isolated in 79% yield. This is an important feature of our reaction, as the isolated sulfonyl chloride might be needed for library synthesis or large scale manufacturing. The high yield of **3u′** is a direct consequence of the mildness of the reaction conditions and the use of an only minimal amount of water (from 32% aqueous HCl). A further reduction of the number of equivalents of 32% aqueous HCl (from 2.0 to 1.2) then also ensured good isolated yields for heterocyclic anilines. Using this modification, substituted pyridine (**3aa** and **3ab**), benzothiophene (**3ac**), and thiophene (**3ad**) examples were all successfully prepared. To confirm that our new Sandmeyer chlorosulfonylation reaction proceeded via the typical diazonium mechanism, the following control experiment was performed. *p*-Anisidine was treated with 37% aqueous HCl in MeCN, followed by slow addition of *tert*-butyl nitrite at rt. After full conversion of *p*-anisidine and formation of the diazonium intermediate, DABSO and CuCl_2_ were added, and the reaction mixture was heated to 75 °C. After 2 h at this temperature, cooling to rt, and aqueous workup, the corresponding sulfonyl chloride **3u′** was isolated in 83% yield as determined by quantitative ^1^H NMR spectroscopy using dibromomethane as an internal standard (see the Supporting Information for details).

**Scheme 3:**
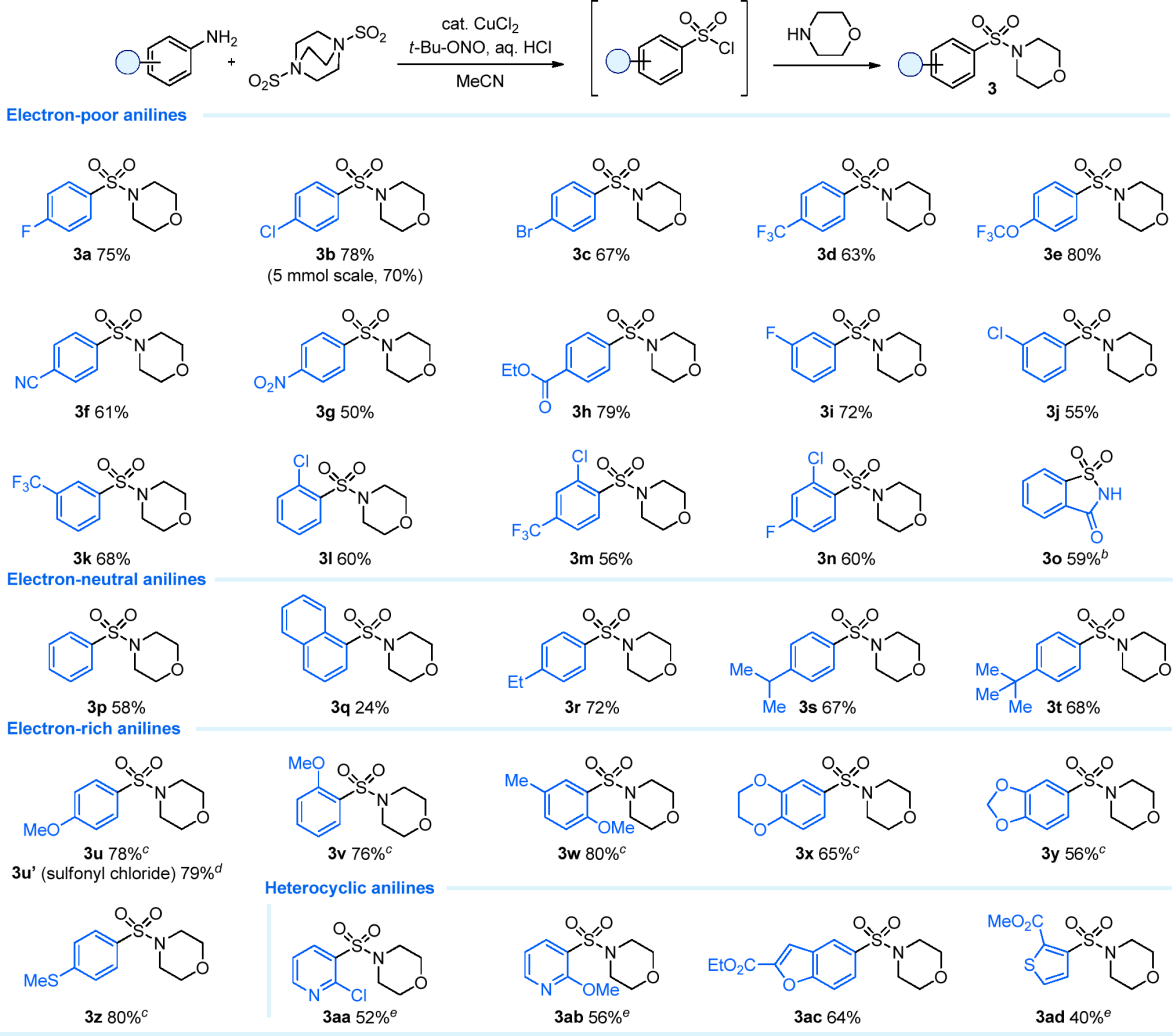
DABSO Sandmeyer Reaction for the Synthesis of Sulfonamides from Aniline Substrates Reaction conditions: aniline substrate (1.0 equiv), DABSO (0.60 equiv), CuCl_2_ (5 mol %), and 37% aqueous HCl (2.0 equiv) in MeCN (0.2 M) at rt, then dropwise addition of *tert*-butyl nitrite (1.1 equiv); after 17 h cool to 0 °C and add morpholine (2.2 equiv). Isolated yields. Saccharin isolated with no morpholine being added. After the addition of *tert*-butyl nitrite, the reaction mixture was heated at 75 °C for 2 h. Sulfonyl chloride isolated with no morpholine being added. With 1.2 equiv of 37% aqueous HCl.

In conclusion, we have developed a novel Sandmeyer-type sulfonyl chloride synthesis from feedstock anilines with DABSO as stable SO_2_ surrogate, in the presence of HCl and a Cu catalyst. Due to the mildness of our reaction conditions, a wide range of carbo- and heterocyclic anilines were successfully converted into their corresponding sulfonyl chlorides or sulfonamides in high yields. Importantly, the aryl diazonium intermediate did not need to be isolated or preformed but was generated *in situ* without any accumulation of this highly energetic species, which makes our protocol inherently safe and operationally simple. Because of these advantages, the reaction was successfully performed on up to 20 g scale and the heterocyclic sulfonyl chloride isolated in 80% yield. We believe that our novel Sandmeyer chlorosulfonylation methodology will find widespread applications in academic and industrial laboratories, as witnessed by the rapid implementation of this new reaction in the medicinal chemistry laboratories at Idorsia Pharmaceuticals Ltd.

## Data Availability

The data underlying this study are available in the published article and its Supporting Information.

## Supporting Information Available

Experimental procedures and supporting characterization data and spectra (PDF)
