# Supplementary material for: Sandmeyer Chlorosulfonylation of (Hetero)Aromatic Amines Using DABSO as an SO2 Surrogate
Source: Org Lett. 2024 Jul 11;26(28):5951–5. doi: 10.1021/acs.orglett.4c01908 (PMC11267597; doi:10.1021/acs.orglett.4c01908)
Supplement: Supplementary file 1 — ol4c01908_si_001.pdf [file ol4c01908_si_001.pdf]

Supporting Information

The Sandmeyer Chlorosulfonylation of (Hetero)Aromatic Amines using DABSO as an SO_2-Surrogate

Lucia Pincekova,[a] Aurélien Merot,[b] Gabriel Schäfer,*,[b,c] Michael C. Willis*,[a]

[a] Department of Chemistry, University of Oxford, Chemistry Research Laboratory, Mansfield Road, Oxford, OX1 3TA, UK. [b] Chemistry Process R&D, Idorsia Pharmaceuticals Ltd., Hegenheimermattweg 91, CH-4123 Allschwil, Switzerland. [c] Biosynth AG, Rietlistrasse 4, CH-9422 Staad, Switzerland.

Emails: schaefer.gabri@gmail.com; michael.willis@chem.ox.ac.uk

Table of Contents

1. General Experimental Considerations 3

2. Reaction Optimization 4

3. Experimental Procedures and Characterization Data 5

3.1 General Procedure A – Synthesis of Sulfonamides – Electron-poor and Electron-neutral Anilines 5

3.2 General Procedure B – Synthesis of Sulfonamides – Electron-rich Anilines 17

3.3 General Procedure C – Synthesis of Sulfonamides – Heterocyclic Anilines 21

3.4 Synthesis of Sulfonylchloride – 4-Methoxybenzenesulfonyl chloride (3u') 24

3.5 Scale-up – 4-((4-Chlorophenyl)sulfonyl)morpholine (3b) 25

3.6 Scale-up – 2-fluoropyridine-3-sulfonyl chloride (1) 25

3.7 Control Experiment with pre-formed diazonium salt 26

4. Process Safety Measurements 28

4.1 RC-1 Run 28

4.2 DSC Measurements 30

5. NMR spectra 31

5. GCMS – 4-Methoxybenzenesulfonyl chloride (3u') 72

6. References 73

1. General Experimental Considerations

Handling techniques: Unless otherwise stated, all reactions were conducted under an atmosphere of nitrogen with anhydrous solvents using standard Schlenk techniques. Cooling of reaction mixtures to 0 °C was achieved using an ice-water bath. 'Room temperature' refers to an ambient temperature of 21 ± 2 °C. The 20 g scale up was performed in a 1.0 L Sulfonation flask with a Teflon overhead stirrer under N_2-atmosphere.

Reagents: Unless otherwise stated, all chemicals were purchased from commercial sources (Sigma-Aldrich, Fluorochem, Fisher Scientific, Alfa-Aesar or Apollo Scientific) and were used without further purification. For the scale up, DABSO was purchased from Combi-Blocks. Anhydrous solvents were purified by filtration through dried alumina columns using the University of Oxford internal solvent drying system (Innovative Technology Inc. PS-400-7) and sparged with nitrogen before use. All inert gases were sourced from the University of Oxford internal supplies and dried through $CaCl_2$ drying columns. 'Petrol' refers to the fraction of petroleum ether which boils in the range 40 – 60 °C.

Chromatography: Thin-layer chromatography (TLC) was performed on Merck silica gel 60 F_{254} pre-coated aluminium backed TLC sheets with a visualization under a UV lamp (λ_{max} = 254 nm) and/or by staining with $KMnO_4$ solution. Flash column chromatography (FCC) was performed using Merck silica gel 60 (230-400 mesh) with the solvent system indicated in parenthesis.

NMR Spectroscopy: ^{1}H NMR spectra were recorded on a Bruker AVIIIHD 400 spectrometer at 400 MHz. ^{13}C NMR spectra were recorded on a Bruker AVIIIHD 400 spectrometer at 101 MHz. ^{19}F NMR spectra were recorded on a Bruker AVIIIHD 400 spectrometer at 377 MHz. All reported ^{1}H and ^{13}C chemical shifts (δ_H, δ_C) are referenced to the residual signal of deuterated solvents ($CDCl_3$: δ_H = 7.26 ppm, δ_C = 77.16 ppm; $(CD_3)_2SO$: δ_H = 2.50 ppm, δ_C = 39.52 ppm; CD_3OD: δ_H = 3.31 ppm, δ_C = 49.00 ppm). ^{19}F chemical shifts (δ_F) are referenced externally to $CFCl_3$ (δ_F = 0.0 ppm). Chemical shifts (δ) are reported in parts per million (ppm) to the nearest 0.01 ppm for ^{1}H NMR, and 0.1 ppm for ^{13}C and ^{19}F NMR. Coupling constants (*J*) are reported in Hertz (Hz) and rounded to the nearest 0.5 Hz. Multiplicities are reported as followings: s = singlet, d = doublet, t = triplet, q = quartet, m = multiplet, bs = broad singlet, "t" for dd with two identical or similar coupling constants, "dt" or "td" for ddd with two identical or similar coupling constants.

IR Spectroscopy: Infrared spectra were recorded on a Bruker Tensor 27 Fourier Transform spectrometer with an internal range 600 – 4000 cm^{-1} and all absorption maximum (ν_{max}) are given in wavenumbers (cm^{-1}).

Melting point: Melting points were recorded in degrees Celsius (°C) using a STUART scientific hot-stage microscope apparatus SMP1 or a Reichert melting point apparatus and are reported uncorrected.

Mass Spectroscopy: High resolution mass spectra (HRMS) were recorded on a Bruker Daltonic µTOF spectrometer through electrospray ionization (ESI) by the mass spectrometry service at Chemistry Research Laboratory, University of Oxford. m/z values are reported in Daltons (Da) and high-resolution values are calculated to four decimal places from the molecular formula. Samples for mass spectra were prepared in 1 mg/mL solution in MeCN or MeOH (HRMS-ESI).

2. Reaction Optimization

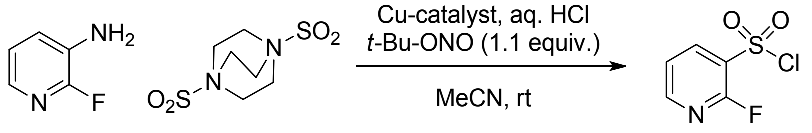

2 DABSO 1

Entry	HCl source	Cu-catalyst	Conversion/overall purity[a]
1	32% aq. HCl (2.0 eq.)	CuCl (0.1 eq.)	>99%, 74% a/a
2	32% aq. HCl (2.0 eq.)	$CuCl_2$ (0.1 eq.)	>99%, 82% a/a
3	32% aq. HCl (2.0 eq.)	$CuCl_2$ (0.025 eq.)	>99%, 82% a/a
4	32% aq. HCl (1.0 eq.)	$CuCl_2$ (0.025 eq.)	>99%, 71% a/a
5	25% aq. HCl (2.0 eq.)	$CuCl_2$ (0.025 eq.)	>99%, 74% a/a
6	2 M aq. HCl (2.0 eq.)	$CuCl_2$ (0.025 eq.)	>99%, 64% a/a
7	5-6 M HCl in i-PrOH (2.0 eq.)	$CuCl_2$ (0.025 eq.)	75%, 40% a/a

Reaction conditions: A 100 mL three-necked flask under N_2-atmosphere was charged with **2** (1.0 g, 8.92 mmol, 1.0 equiv.), **DABSO** (1.31 g, 5.35 mmol, 0.60 equiv.) and the Cu-catalyst in MeCN (10 mL) and HCl (2.0 equiv.). Then, *tert*-butyl nitrite 90% (1.3 mL, 9.81 mmol, 1.1 equiv.) was added dropwise via syringe pump over 15 min at rt. After 1 h at rt, the sampling for LC/MS analysis was performed.
[a] The conversion was judged by the consumption of starting material **2** relative to the formation of the desired product **1** by LC/MS at 210 nm; Overall purity refers to area/area (a/a)-percentage of desired product **1** after 1 h by LC/MS at 210 nm.

3. Experimental Procedures and Characterization Data

3.1 General Procedure A – Synthesis of Sulfonamides – Electron-poor and Electron-neutral Anilines

Round-bottom flask containing the corresponding amine (1.0 equiv.), DABSO (0.60 equiv.), $CuCl_2$ catalyst (5 mol%) was sealed and subjected to three N_2 evacuation/refill cycles before pre-sparged anhydrous MeCN (amine conc. 0.2 M) was added. The resulting solution was placed to cold-water bath (18 °C) and 37% aq. HCl (2.0 equiv.) was added dropwise (slightly exothermic 22 °C to 24 °C). After 10 min, *tert*-butyl nitrite 90% (1.1 equiv.) was added dropwise (slightly exothermic: 22 °C to 29 °C). After 10 min cold-water bath was removed and rection mixture was stirred overnight. After 17 h reaction mixture was cooled down to 0 °C and morpholine (2.2 equiv.) was added dropwise at the same temperature. After addition of morpholine the reaction mixture was warmed to room temperature and stirred for another 30 minutes before being quenched with saturated aqueous solution of NH_4Cl (6.0 mL). The resulting mixture was then extracted with EtOAc (3 x 10 mL). Collected organic phases were dried over anhydrous Na_2SO_4, filtered and concentrated *in vacuo*. The resulting residue was then purified by flash column chromatography with silica gel under standard eluent mixture (typically ethyl acetate [EtOAc] in petroleum ether 40 – 60 °C bp [PE]). The solvents were removed *in vacuo* and the products were finally dried under high vacuum.

4-((4-Fluorophenyl)sulfonyl)morpholine (3a)

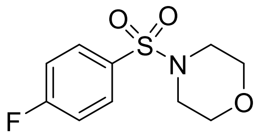

3a

Prepared according to general procedure A, using 4-fluroaniline (47 µL, 0.50 mmol, 1.0 equiv.), DABSO (72.1 mg, 0.30 mmol, 0.60 equiv.), $CuCl_2$ (3.4 mg, 0.025 mmol, 0.050 equiv.), MeCN (2.5 mL), 37% aq. HCl (84 µL, 1.0 mmol, 2.0 equiv.), *tert*-butyl nitrite 90% (73 µL, 0.55 mmol, 1.1 equiv.). Stirred at rt for 17 h, then morpholine (95 µL, 1.1 mmol, 2.2 equiv.). Flash column chromatography (SiO_2, PE:EtOAc = 4:1) afforded the desired sulfonamide **3a** as an off-white solid (92 mg, 0.38 mmol, 75%).

^{1}H NMR (400 MHz, $CDCl_3$) δ = 7.83 – 7.73 (m, 2H), 7.29 – 7.19 (m, 2H), 3.75 ("t", *J* = 4.8 Hz, 4H), 3.00 ("t", *J* = 4.8 Hz, 4H).

^{13}C NMR (101 MHz, $CDCl_3$) δ = 165.5 (d, *J* = 255.5 Hz), 131.4 (d, *J* = 3.2 Hz), 130.7 (d, *J* = 9.1 Hz), 116.6 (d, *J* = 22.6 Hz), 66.2, 46.1.

^{19}F NMR (377 MHz, $CDCl_3$) δ = -104.5.

HRMS (ESI$^+$, m/z): calculated for $C_{10}H_{12}FNO_3SNa$ $[M+Na]^+$, 268.0414, found 268.0422.

Data is in agreement with that previously reported.[1]

4-((4-Chlorophenyl)sulfonyl)morpholine (3b)

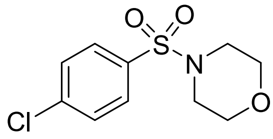

3b

Prepared according to general procedure A, using 4-chloroaniline (63.8 mg, 0.50 mmol, 1.0 equiv.), DABSO (72.1 mg, 0.30 mmol, 0.60 equiv.), $CuCl_2$ (3.4 mg, 0.025 mmol, 0.050 equiv.), MeCN (2.5 mL), 37% aq. HCl (84 µL, 1.0 mmol, 2.0 equiv.), *tert*-butyl nitrite 90% (73 µL, 0.55 mmol, 1.1 equiv.). Stirred at rt for 17 h, then morpholine (95 µL, 1.1 mmol, 2.2 equiv.). Flash column chromatography (SiO_2, PE:EtOAc = 4:1 to 7:3) afforded the desired sulfonamide **3b** as an orange solid (102 mg, 0.39 mmol, 78%).

^{1}H NMR (400 MHz, $CDCl_3$) δ = 7.73 – 7.65 (m, 2H), 7.57 – 7.49 (m, 2H), 3.74 ("t", *J* = 4.8 Hz, 4H), 3.00 ("t", *J* = 4.8 Hz, 4H).

^{13}C NMR (101 MHz, $CDCl_3$) δ = 139.9, 133.9, 129.6, 129.4, 66.2, 46.1.

HRMS (ESI$^+$, m/z): calculated for $C_{10}H_{12}ClNO_3SNa$ $[M+Na]^+$, 284.0119, found 284.0124.

Data is in agreement with that previously reported.[2]

4-((4-Bromophenyl)sulfonyl)morpholine (3c)

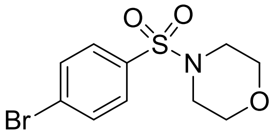

3c

Prepared according to general procedure A, using 4-bromoaniline (86.0 mg, 0.50 mmol, 1.0 equiv.), DABSO (72.1 mg, 0.30 mmol, 0.60 equiv.), $CuCl_2$ (3.4 mg, 0.025 mmol, 0.050 equiv.), MeCN (2.5 mL), 37% aq. HCl (84 µL, 1.0 mmol, 2.0 equiv.), *tert*-butyl nitrite 90% (73 µL, 0.55 mmol, 1.1 equiv.). Stirred at rt for 17 h, then morpholine (95 µL, 1.1 mmol, 2.2 equiv.). Flash column chromatography (SiO_2, PE:EtOAc = 9:1 to 4:1) afforded the desired sulfonamide **3c** as a pale-yellow solid (102 mg, 0.33 mmol, 67%).

^{1}H NMR (400 MHz, $CDCl_3$) δ = 7.74 – 7.66 (m, 2H), 7.63 – 7.59 (m, 2H), 3.74 ("t", *J* = 4.7 Hz, 4H), 2.99 ("t", *J* = 4.7 Hz, 4H).

^{13}C NMR (101 MHz, $CDCl_3$) δ = 134.3, 132.6, 129.4, 128.4, 66.2, 46.0.

HRMS (ESI$^+$, m/z): calculated for $C_{10}H_{13}BrNO_3S$ $[M+H]^+$, 305.9794, found 305.9799.

Data is in agreement with that previously reported.[3]

4-((4-(Trifluoromethyl)phenyl)sulfonyl)morpholine (3d)

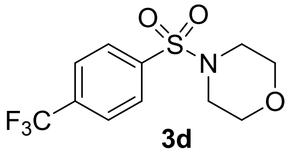

3d

Prepared according to general procedure A, using 4-(trifluoromethyl)aniline (63 µL, 0.50 mmol, 1.0 equiv.), DABSO (72.1 mg, 0.30 mmol, 0.60 equiv.), $CuCl_2$ (3.4 mg, 0.025 mmol, 0.050 equiv.), MeCN (2.5 mL), 37% aq. HCl (84 µL, 1.0 mmol, 2.0 equiv.), *tert*-butyl nitrite 90% (73 µL, 0.55 mmol, 1.1 equiv.). Stirred at rt for 17 h, then morpholine (95 µL, 1.1 mmol, 2.2 equiv.). Flash column chromatography (SiO_2, PE:EtOAc = 9:1 to 4:1) afforded the desired sulfonamide **3d** as a pale-yellow solid (93 mg, 0.32 mmol, 63%).

^{1}H NMR (400 MHz, $CDCl_3$) δ = 7.89 (d, *J* = 8.2 Hz, 2H), 7.83 (d, *J* = 8.2 Hz, 2H), 3.76 ("t", *J* = 4.8 Hz, 4H), 3.03 ("t", *J* = 4.8 Hz, 4H).

^{13}C NMR (101 MHz, $CDCl_3$) δ = 139.1, 134.9 (d, *J* = 33.0 Hz), 128.5, 126.5 (q, *J* = 3.8 Hz), 123.3 (t, *J* = 273.0 Hz), 66.2, 46.1.

^{19}F NMR (377 MHz, $CDCl_3$) δ = -63.1.

HRMS (ESI$^+$, m/z): calculated for $C_{11}H_{13}F_3NO_3S$ $[M+H]^+$, 296.0563, found 296.0566.

Data is in agreement with that previously reported.[1]

4-((4-(Trifluoromethoxy)phenyl)sulfonyl)morpholine (3e)

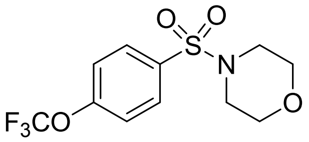

3e

Prepared according to general procedure A, using 4-(trifluoromethoxy)aniline (67 µL, 0.50 mmol, 1.0 equiv.), DABSO (72.1 mg, 0.30 mmol, 0.60 equiv.), $CuCl_2$ (3.4 mg, 0.025 mmol, 0.050 equiv.), MeCN (2.5 mL), 37% aq. HCl (84 µL, 1.0 mmol, 2.0 equiv.), *tert*-butyl nitrite 90% (73 µL, 0.55 mmol, 1.1 equiv.). Stirred at rt for 17 h, then morpholine (95 µL, 1.1 mmol, 2.2 equiv.). Flash column chromatography (SiO_2, PE:EtOAc = 4:1 to 7:3) afforded the desired sulfonamide **3e** as a pale-pink solid (124 mg, 0.40 mmol, 80%).

^{1}H NMR (400 MHz, $CDCl_3$) δ = 7.86 – 7.77 (m, 2H), 7.43 – 7.34 (m, 2H), 3.76 (“t”, *J* = 4.8 Hz, 4H), 3.02 (“t”, *J* = 4.8 Hz, 4H).

^{13}C NMR (101 MHz, $CDCl_3$) δ = 152.6, 133.8, 130.1, 121.1, 120.3 (q, *J* = 259.9 Hz), 66.2, 46.1.

^{19}F NMR (377 MHz, $CDCl_3$) δ = -57.7.

HRMS (ESI$^+$, m/z): calculated for $C_{11}H_{12}F_3NO_4SNa$ $[M+Na]^+$, 334.0331, found 334.0335.

Data is in agreement with that previously reported.[1]

4-(Morpholinosulfonyl)benzonitrile (3f)

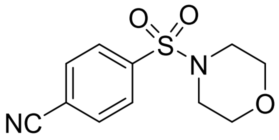

3f

Prepared according to general procedure A, using 4-aminobenzonitrile (59.1 mg, 0.50 mmol, 1.0 equiv.), DABSO (72.1 mg, 0.30 mmol, 0.60 equiv.), $CuCl_2$ (3.4 mg, 0.025 mmol, 0.050 equiv.), MeCN (2.5 mL), 37% aq. HCl (84 µL, 1.0 mmol, 2.0 equiv.), *tert*-butyl nitrite 90% (73 µL, 0.55 mmol, 1.1 equiv.). Stirred at rt for 17 h, then morpholine (95 µL, 1.1 mmol, 2.2 equiv.). Flash column chromatography (SiO_2, PE:EtOAc = 3:2) afforded the desired sulfonamide **3f** as a pale-yellow solid (76.4 mg, 0.30 mmol, 61%).

^{1}H NMR (400 MHz, $CDCl_3$) δ = 7.87 (s, 4H), 3.75 (“t”, *J* = 4.8 Hz, 4H), 3.03 (“t”, *J* = 4.8 Hz, 4H).

^{13}C NMR (101 MHz, $CDCl_3$) δ = 139.9, 133.1, 128.5, 117.3, 117.0, 66.1, 46.0.

HRMS (ESI$^+$, m/z): calculated for $C_{11}H_{12}N_2O_3S$ $[M+Na]^+$, 275.0461, found 275.0448.

Data is in agreement with that previously reported.[2]

4-((4-Nitrophenyl)sulfonyl)morpholine (3g)

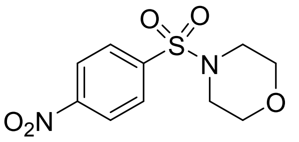

3g

Prepared according to general procedure A, using 4-nitroaniline (69.1 mg, 0.50 mmol, 1.0 equiv.), DABSO (72.1 mg, 0.30 mmol, 0.60 equiv.), $CuCl_2$ (3.4 mg, 0.025 mmol, 0.050 equiv.), MeCN (2.5 mL), 37% aq. HCl (84 µL, 1.0 mmol, 2.0 equiv.), *tert*-butyl nitrite 90% (73 µL, 0.55 mmol, 1.1 equiv.). Stirred at rt for 17 h, then morpholine (95 µL, 1.1 mmol, 2.2 equiv.). Flash column chromatography (SiO_2, PE:EtOAc = 4:1) afforded the desired sulfonamide **3g** as an orange solid (68 mg, 0.25 mmol, 50%).

^{1}H NMR (400 MHz, $CDCl_3$) δ = 8.45 – 8.37 (m, 2H), 7.99 – 7.91 (m, 2H), 3.76 (“t”, *J* = 4.7 Hz, 4H), 3.06 (“t”, *J* = 4.8 Hz, 4H).

^{13}C NMR (101 MHz, $CDCl_3$) δ = 150.5, 141.4, 129.1, 124.6, 66.1, 46.0.

HRMS (ESI$^+$, m/z): calculated for $C_{10}H_{12}N_2O_5SNa$ $[M+Na]^+$, 295.0359, found 295.0341.

Data is in agreement with that previously reported.[4]

Ethyl 4-(morpholinosulfonyl)benzoate (3h)

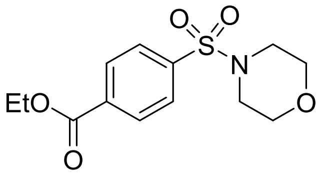

3h

Prepared according to general procedure A, using ethyl 4-aminobenzoate (82.6 mg, 0.50 mmol, 1.0 equiv.), DABSO (72.1 mg, 0.30 mmol, 0.60 equiv.), $CuCl_2$ (3.4 mg, 0.025 mmol, 0.050 equiv.), MeCN (2.5 mL), 37% aq. HCl (84 µL, 1.0 mmol, 2.0 equiv.), *tert*-butyl nitrite 90% (73 µL, 0.55 mmol, 1.1 equiv.). Stirred at rt for 17 h, then morpholine (95 µL, 1.1 mmol, 2.2 equiv.). Flash column chromatography (SiO_2, PE:EtOAc = 4:1 to 7:3) afforded the desired sulfonamide **3h** as a pale-yellow solid (118 mg, 0.39 mmol, 79%).

^{1}H NMR (400 MHz, $CDCl_3$) δ = 8.25 – 8.18 (m, 2H), 7.86 – 7.78 (m, 2H), 4.43 (q, *J* = 7.1 Hz, 2H), 3.74 (“t”, *J* = 4.7 Hz, 4H), 3.02 (“t”, *J* = 4.7 Hz, 4H), 1.42 (t, *J* = 7.1 Hz, 3H).

^{13}C NMR (101 MHz, $CDCl_3$) δ = 165.2, 139.2, 134.7, 130.4, 127.9, 66.2, 61.9, 46.1, 14.4.

HRMS (ESI$^+$, m/z): calculated for $C_{13}H_{17}NO_5SNa$ $[M+Na]^+$, 322.0720, found 322.0732.

Data is in agreement with that previously reported.[5]

4-((3-Fluorophenyl)sulfonyl)morpholine (3i)

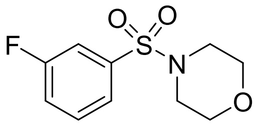

3i

Prepared according to general procedure A, using 3-fluoroaniline (48 µL, 0.50 mmol, 1.0 equiv.), DABSO (72.1 mg, 0.30 mmol, 0.60 equiv.), $CuCl_2$ (3.4 mg, 0.025 mmol, 0.050 equiv.), MeCN (2.5 mL), 37% aq. HCl (84 µL, 1.0 mmol, 2.0 equiv.), *tert*-butyl nitrite 90% (73 µL, 0.55 mmol, 1.1 equiv.). Stirred at rt for 17 h, then morpholine (95 µL, 1.1 mmol, 2.2 equiv.). Flash column chromatography (SiO_2, PE:EtOAc = 9:1 to 4:1) afforded the desired sulfonamide **3i** as a white solid (88 mg, 0.36 mmol, 72%).

^{1}H NMR (400 MHz, $CDCl_3$) δ = 7.58 – 7.53 (m, 2H), 7.50 – 7.43 (m, 1H), 7.40 – 7.28 (m, 1H), 3.75 (“t”, *J* = 4.8 Hz, 4H), 3.02 (“t”, *J* = 4.8 Hz, 4H).

^{13}C NMR (101 MHz, $CDCl_3$) δ = 162.6 (d, *J* = 251.9 Hz), 137.4 (d, *J* = 6.8 Hz), 131.1 (d, *J* = 7.5 Hz), 123.7 (d, *J* = 3.2 Hz), 120.5 (d, *J* = 21.1 Hz), 115.3 (d, *J* = 24.2 Hz), 66.2, 46.1.

^{19}F NMR (377 MHz, $CDCl_3$) δ = -109.2.

HRMS (ESI$^+$, m/z): calculated for $C_{10}H_{13}FNO_3S$ $[M+H]^+$, 246.0595, found 246.0597.

Data is in agreement with that previously reported.[1]

4-((3-Chlorophenyl)sulfonyl)morpholine (3j)

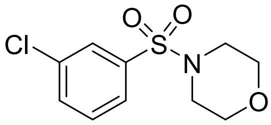

3j

Prepared according to general procedure A, using 3-chloroaniline (53 µL, 0.50 mmol, 1.0 equiv.), DABSO (72.1 mg, 0.30 mmol, 0.60 equiv.), $CuCl_2$ (3.4 mg, 0.025 mmol, 0.050 equiv.), MeCN (2.5 mL), 37% aq. HCl (84 µL, 1.0 mmol, 2.0 equiv.), *tert*-butyl nitrite 90% (73 µL, 0.55 mmol, 1.1 equiv.). Stirred at rt for 17 h, then morpholine (95 µL, 1.1 mmol, 2.2 equiv.). Flash column chromatography (SiO_2, PE:EtOAc = 9:1 to 4:1) afforded the desired sulfonamide **3j** as a white solid (71 mg, 0.27 mmol, 55%).

^{1}H NMR (400 MHz, $CDCl_3$) δ = 7.74 (t, *J* = 1.9 Hz, 1H), 7.64 (“dt”, *J* = 7.7, 1.5 Hz, 1H), 7.60 (ddd, *J* = 8.1, 2.1, 1.1 Hz, 1H), 7.51 (t, *J* = 7.9 Hz, 1H), 3.75 (“t”, *J* = 4.7 Hz, 4H), 3.02 (“t”, *J* = 4.7 Hz, 4H).

^{13}C NMR (101 MHz, $CDCl_3$) δ = 137.1, 135.7, 133.4, 130.6, 127.9, 126.0, 66.2, 46.1.

HRMS (ESI$^+$, m/z): calculated for $C_{10}H_{13}ClNO_3S$ $[M+H]^+$, 262.0299, found 262.0307.

Data is in agreement with that previously reported.[1]

4-((3-(Trifluoromethyl)phenyl)sulfonyl)morpholine (3k)

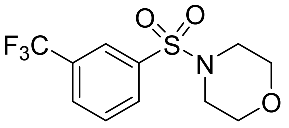

3k

Prepared according to general procedure A, using 3-(trifluoromethyl)aniline (63 µL, 0.50 mmol, 1.0 equiv.), DABSO (72.1 mg, 0.30 mmol, 0.60 equiv.), $CuCl_2$ (3.4 mg, 0.025 mmol, 0.050 equiv.), MeCN (2.5 mL), 37% aq. HCl (84 µL, 1.0 mmol, 2.0 equiv.), *tert*-butyl nitrite 90% (73 µL, 0.55 mmol, 1.1 equiv.). Stirred at rt for 17 h, then morpholine (95 µL, 1.1 mmol, 2.2 equiv.). Flash column chromatography (SiO_2, PE:EtOAc = 4:1) afforded the desired sulfonamide **3k** as a white solid (100 mg, 0.34 mmol, 68%).

Mp 104 – 106 °C.

^{1}H NMR (400 MHz, $CDCl_3$) δ = 8.02 (s, 1H), 7.95 (d, *J* = 8.0 Hz, 1H), 7.90 (d, *J* = 7.9 Hz, 1H), 7.73 (t, *J* = 7.8 Hz, 1H), 3.76 (“t”, *J* = 4.7 Hz, 4H), 3.03 (“t”, *J* = 4.7 Hz, 4H).

^{13}C NMR (101 MHz, $CDCl_3$) δ = 136.8, 131.9 (q, *J* = 33.8 Hz), 131.1, 130.1, 129.9 (q, *J* = 3.6 Hz), 124.9 (q, *J* = 3.8 Hz), 123.3 (q, *J* = 273.2 Hz), 66.2, 46.0.

^{19}F NMR (377 MHz, $CDCl_3$) δ = -62.8.

HRMS (ESI$^+$, m/z): calculated for $C_{11}H_{16}F_3N_2O_3S$ $[M+NH_4]^+$, 313.0828, found 313.0830.

IR (ν_{max}, cm^{-1}) 3089, 2988, 2924, 2858, 1611, 1353, 1330, 1315, 1173, 1130, 1072, 946, 816, 739, 696, 645.

4-((2-Chlorophenyl)sulfonyl)morpholine (3l)

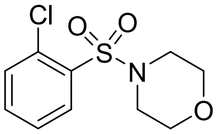

3l

Prepared according to general procedure A, using 2-chloroaniline (53 µL, 0.50 mmol, 1.0 equiv.), DABSO (72.1 mg, 0.30 mmol, 0.60 equiv.), $CuCl_2$ (3.4 mg, 0.025 mmol, 0.050 equiv.), MeCN (2.5 mL), 37% aq. HCl (84 µL, 1.0 mmol, 2.0 equiv.), *tert*-butyl nitrite 90% (73 µL, 0.55 mmol, 1.1 equiv.). Stirred at rt for 17 h, then morpholine (95 µL, 1.1 mmol, 2.2 equiv.). Flash column chromatography (SiO_2, PE:EtOAc = 4:1) afforded the desired sulfonamide **3l** as a white solid (78 mg, 0.30 mmol, 60%). **Mp** 92 – 94 °C.

^{1}H NMR (400 MHz, $CDCl_3$) δ = 8.04 (dd, *J* = 8.0, 1.5 Hz, 1H), 7.55 (dd, *J* = 7.8, 1.7 Hz, 1H), 7.51 ((“td”, *J* = 7.5, 1.5 Hz, 1H), 7.41 (ddd, *J* = 7.9, 7.0, 1.7 Hz, 1H), 3.73 (“t”, *J* = 4.7 Hz, 4H), 3.29 (“t”, *J* = 4.7 Hz, 4H).

^{13}**C NMR** (101 MHz, $CDCl_3$) δ = 135.7, 134.0, 132.5, 132.4, 132.3, 127.2, 66.6, 45.9.

HRMS (ESI$^+$, m/z): calculated for $C_{10}H_{12}ClNO_3SK$ [M+K]$^+$, 299.9858, found 299.9860.

IR (ν_{max}, cm^{-1}) 3097, 2920, 2896, 2862, 2360, 2341, 1578, 1454, 1358, 1262, 1171, 1116, 953, 758, 702, 613.

4-((2-Chloro-4-(trifluoromethyl)phenyl)sulfonyl)morpholine (3m)

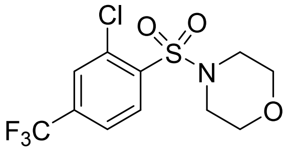

3m

Prepared according to general procedure A, using 2-chloro-4-(trifluoromethyl)aniline (71 µL, 0.50 mmol, 1.0 equiv.), DABSO (72.1 mg, 0.30 mmol, 0.60 equiv.), $CuCl_2$ (3.4 mg, 0.025 mmol, 0.050 equiv.), MeCN (2.5 mL), 37% aq. HCl (84 µL, 1.0 mmol, 2.0 equiv.), *tert*-butyl nitrite 90% (73 µL, 0.55 mmol, 1.1 equiv.). Stirred at rt for 17 h, then morpholine (95 µL, 1.1 mmol, 2.2 equiv.). Flash column chromatography (SiO_2, PE:EtOAc = 4:1) afforded the desired sulfonamide **3m** as a colourless oil (93 mg, 0.28 mmol, 56%).

^{1}**H NMR** (400 MHz, $CDCl_3$) δ = 8.18 (dd, *J* = 8.2, 1.0 Hz, 1H), 7.83 – 7.78 (m, 1H), 7.67 (ddd, *J* = 8.2, 1.7, 0.8 Hz, 1H), 3.74 ("t", *J* = 4.7 Hz, 4H), 3.32 ("t", *J* = 4.7 Hz, 4H).

^{13}**C NMR** (101 MHz, $CDCl_3$) δ = 139.6, 135.8 (d, *J* = 33.8 Hz), 133.4, 132.8, 129.5 (q, *J* = 3.8 Hz), 124.1 (q, *J* = 3.7 Hz), 122.6 (d, *J* = 273.4 Hz), 66.6, 46.0.

^{19}**F NMR** (377 MHz, $CDCl_3$) δ = -63.3.

HRMS (ESI$^+$, m/z): calculated for $C_{11}H_{12}ClF_3NO_3S$ [M+H]$^+$, 330.0173, found 330.0184.

IR (ν_{max}, cm^{-1}) 3105, 2969, 2928, 2863, 1453, 1390, 1326, 1178, 1144, 955, 842, 734, 646.

4-((2-Chloro-4-fluorophenyl)sulfonyl)morpholine (3n)

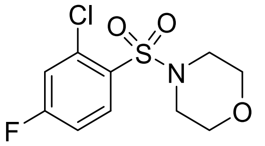

3n

Prepared according to general procedure A, using 2-chloro-4-fluoroaniline (60 µL, 0.50 mmol, 1.0 equiv.), DABSO (72.1 mg, 0.30 mmol, 0.60 equiv.), $CuCl_2$ (3.4 mg, 0.025 mmol, 0.050 equiv.), MeCN (2.5

mL), 37% aq. HCl (84 µL, 1.0 mmol, 2.0 equiv.), *tert*-butyl nitrite 90% (73 µL, 0.55 mmol, 1.1 equiv.). Stirred at rt for 17 h, then morpholine (95 µL, 1.1 mmol, 2.2 equiv.). Flash column chromatography (SiO_2, PE:EtOAc = 4:1) afforded the desired sulfonamide **3n** as pale-yellow solid (83 mg, 0.30 mmol, 60%).

Mp 63 – 66 °C.

^{1}H NMR (400 MHz, $CDCl_3$) δ = 8.06 (ddd, *J* = 8.8, 5.9, 1.3 Hz, 1H), 7.29 (ddd, *J* = 8.3, 2.6, 1.0 Hz, 1H), 7.16 – 7.07 (m, 1H), 3.72 ("td", *J* = 4.7, 1.3 Hz, 4H), 3.27 ("td", *J* = 4.8, 1.5 Hz, 4H).

^{13}C NMR (101 MHz, $CDCl_3$) δ = 166.0, 163.5, 134.4 (d, *J* = 9.8 Hz), 132.1 (d, *J* = 3.7 Hz), 119.9 (d, *J* = 25.3 Hz), 114.5 (d, *J* = 21.5 Hz), 66.6, 45.9.

^{19}F NMR (377 MHz, $CDCl_3$) δ -103.4.

HRMS (ESI$^+$, m/z): calculated for $C_{10}H_{11}ClFNO_3SNa$ $[M+Na]^+$, 302.0024, found 302.0033.

IR (ν_{max}, cm^{-1}) 3100, 2968, 2921, 2861, 1588, 1471, 1388, 1353, 1263, 1213, 1166, 1115, 1074,954, 908, 727, 649.

Benzo[*d*]isothiazol-3(2*H*)-one 1,1-dioxide (3o)

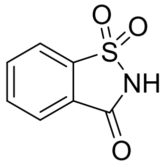

3o

Prepared according to general procedure A, using 2-aminobenzamide (68.1 mg, 0.50 mmol, 1.0 equiv.), DABSO (72.1 mg, 0.30 mmol, 0.60 equiv.), $CuCl_2$ (3.4 mg, 0.025 mmol, 0.050 equiv.), MeCN (2.5 mL), 37% aq. HCl (84 µL, 1.0 mmol, 2.0 equiv.), *tert*-butyl nitrite 90% (73 µL, 0.55 mmol, 1.1 equiv.). Stirred at rt for 17 h. Flash column chromatography (SiO_2, PE:EtOAc = 3:2 to 1:1) afforded saccharine (**3o**) as a colourless solid (53 mg, 0.29 mmol, 59%). **Mp** 201 – 202 °C.

^{1}H NMR (400 MHz, DMSO-d_6) δ = 14.96 (bs, 1H), 8.21 (ddd, *J* = 7.9, 1.5, 0.6 Hz, 1H), 8.18 (ddd, *J* = 8.1, 1.3, 0.6 Hz, 1H), 8.08 (ddd, *J* = 8.1, 7.2, 1.5 Hz, 1H), 7.90 (ddd, *J* = 7.9, 7.2, 1.2 Hz, 1H).

^{13}C NMR (101 MHz, DMSO-d_6) δ = 155.5, 144.2, 135.5, 132.6, 127.9, 124.3, 120.2.

HRMS (ESI$^-$, m/z): calculated for $C_7H_4NO_3S$ $[M-H]^-$, 181.9917, found 181.9930.

IR (ν_{max}, cm^{-1}) 3130, 3106, 3038, 3007, 2864, 2342, 1699, 1459, 1385, 1228, 1137, 949, 785, 670.

4-(Phenylsulfonyl)morpholine (3p)

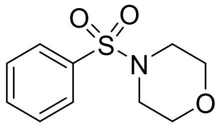

3p

Prepared according to general procedure A, using aniline (46 µL, 0.50 mmol, 1.0 equiv.), DABSO (72.1 mg, 0.30 mmol, 0.60 equiv.), $CuCl_2$ (3.4 mg, 0.025 mmol, 0.050 equiv.), MECN (2.5 mL), 37% HCl (84 µL, 1.0 mmol, 2.0 equiv.), *tert*-butyl nitrite 90% (73 µL, 0.55 mmol, 1.1 equiv.). Stirred at rt for 17 h, then morpholine (95 µL, 1.1 mmol, 2.2 equiv.). Flash column chromatography (SiO_2, PE:EtOAc = 7:3 to 3:2) afforded the desired sulfonamide **3p** as a white solid (66 mg, 0.29 mmol, 58%).

^{1}H NMR (400 MHz, $CDCl_3$) δ = 7.78 – 7.73 (m, 2H), 7.66 – 7.60 (m, 1H), 7.59 – 7.52 (m, 2H), 3.74 ("t", *J* = 4.6 Hz, 4H), 3.00 ("t", *J* = 4.6 Hz, 4H).

^{13}C NMR (101 MHz, $CDCl_3$) δ = 135.2, 133.2, 129.3, 128.0, 66.2, 46.1.

HRMS (ESI$^+$, m/z): calculated for $C_{10}H_{13}NO_3SNa$ $[M+Na]^+$, 250.0508, found 250.0512.

Data is in agreement with that previously reported.[4]

4-(Naphthalen-1-ylsulfonyl)morpholine (3q)

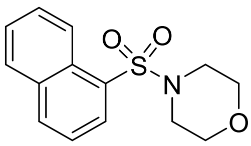

3q

Prepared according to general procedure A, using 1-naphthylamine (71.6 mg, 0.50 mmol, 1.0 equiv.), DABSO (72.1 mg, 0.30 mmol, 0.60 equiv.), $CuCl_2$ (3.4 mg, 0.025 mmol, 0.050 equiv.), MeCN (2.5 mL), 37% aq. HCl (84 µL, 1.0 mmol, 2.0 equiv.), *tert*-butyl nitrite 90% (73 µL, 0.55 mmol, 1.1 equiv.). Stirred at rt for 17 h, then morpholine (95 µL, 1.1 mmol, 2.2 equiv.). Flash column chromatography (SiO_2, PE:EtOAc = 4:1 to 7:3) afforded the desired sulfonamide **3q** as a dark-orange solid (33 mg, 0.12 mmol, 24%).

^{1}H NMR (400 MHz, $CDCl_3$) δ = 8.77 (d, *J* = 8.7 Hz, 1H), 8.21 (dd, *J* = 7.4, 1.3 Hz, 1H), 8.09 (d, *J* = 8.3 Hz, 1H), 7.94 (dd, *J* = 8.0, 1.8 Hz, 1H), 7.69 – 7.53 (m, 3H), 3.68 ("t", *J* = 4.7 Hz, 4H), 3.16 ("t", *J* = 4.7 Hz, 4H).

^{13}C NMR (101 MHz, $CDCl_3$) δ = 134.9, 134.5, 132.0, 131.0, 129.2, 129.1, 128.3, 127.1, 125.2, 124.2, 66.4, 45.8.

HRMS (ESI$^+$, m/z): calculated for $C_{14}H_{15}NO_3SNa$ $[M+Na]^+$, 300.0665, found 300.0678.

Data is in agreement with that previously reported.[1]

4-((4-Ethylphenyl)sulfonyl)morpholine (3r)

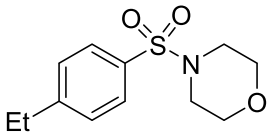

3r

Prepared according to general procedure A, using 4-ethylaniline (62 µL, 0.50 mmol, 1.0 equiv.), DABSO (72.1 mg, 0.30 mmol, 0.60 equiv.), $CuCl_2$ (3.4 mg, 0.025 mmol, 0.050 equiv.), MeCN (2.5 mL), 37% HCl (84 µL, 1.0 mmol, 2.0 equiv.), *tert*-butyl nitrite 90% (73 µL, 0.55 mmol, 1.1 equiv.). Stirred at rt for 17 h, then morpholine (95 µL, 1.1 mmol, 2.2 equiv.). Flash column chromatography (SiO_2, PE:EtOAc = 3:2 to 1:1) afforded the desired sulfonamide **3r** as a pale-orange solid (91 mg, 0.36 mmol, 72%).

^{1}H NMR (400 MHz, $CDCl_3$) δ = 7.70 – 7.62 (m, 2H), 7.40 – 7.32 (m, 2H), 3.74 (“t”, *J* = 4.8 Hz, 4H), 2.99 (“t”, *J* = 4.8 Hz, 4H), 2.74 (q, *J* = 7.6 Hz, 2H), 1.27 (t, *J* = 7.6 Hz, 3H).

^{13}C NMR (101 MHz, $CDCl_3$) δ = 150.2, 132.3, 128.7, 128.1, 66.2, 46.1, 28.9, 15.2.

HRMS (ESI$^+$, m/z): calculated for $C_{12}H_{17}NO_3SNa$ $[M+Na]^+$, 278.0821, found 278.0830.

Data is in agreement with that previously reported.[7]

4-((4-Isopropylphenyl)sulfonyl)morpholine (3s)

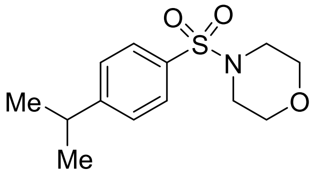

3s

Prepared according to general procedure A, using 4-isopropylaniline (68 µL, 0.50 mmol, 1.0 equiv.), DABSO (72.1 mg, 0.30 mmol, 0.60 equiv.), $CuCl_2$ (3.4 mg, 0.025 mmol, 0.050 equiv.), MeCN (2.5 mL), 37% aq. HCl (84 µL, 1.0 mmol, 2.0 equiv.), *tert*-butyl nitrite 90% (73 µL, 0.55 mmol, 1.1 equiv.). Stirred at rt for 17 h, then morpholine (95 µL, 1.1 mmol, 2.2 equiv.). Flash column chromatography (SiO_2, PE:EtOAc = 7:3 to 3:2) afforded the desired sulfonamide **3s** as an off-white solid (91 mg, 0.34 mmol, 67%). **Mp** 106 – 107 °C.

^{1}H NMR (400 MHz, $CDCl_3$) δ = 7.71 – 7.63 (m, 2H), 7.43 – 7.35 (m, 2H), 3.74 (“t”, *J* = 4.8 Hz, 4H), 3.00 (“t”, *J* = 4.8 Hz, 4H), 1.29 (s, 3H), 1.27 (s, 3H).

^{13}C NMR (101 MHz, $CDCl_3$) δ = 154.7, 132.4, 128.2, 127.3, 66.2, 46.1, 34.3, 23.8.

HRMS (ESI$^+$, m/z): calculated for $C_{13}H_{19}NO_3SNa$ $[M+Na]^+$, 292.0978, found 292.0978.

IR (ν_{max}, cm^{-1}) 3017, 2863, 2360, 2341, 1351, 1170, 1115, 946, 779, 725, 615.

4-((4-(*tert*-Butyl)phenyl)sulfonyl)morpholine (3t)

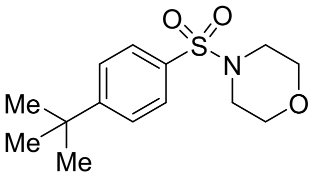

3t

Prepared according to general procedure A, using 4-*tert*-butylaniline (80 µL, 0.50 mmol, 1.0 equiv.), DABSO (72.1 mg, 0.30 mmol, 0.60 equiv.), $CuCl_2$ (3.4 mg, 0.025 mmol, 0.050 equiv.), MeCN (2.5 mL), 37% aq. HCl (84 µL, 1.0 mmol, 2.0 equiv.), *tert*-butyl nitrite 90% (73 µL, 0.55 mmol, 1.1 equiv.). Stirred at rt for 17 h, then morpholine (95 µL, 1.1 mmol, 2.2 equiv.). Flash column chromatography (SiO_2, PE:EtOAc = 7:3 to 3:2) afforded the desired sulfonamide **3t** as an off-white solid (96 mg, 0.34 mmol, 68%).

^{1}H NMR (400 MHz, $CDCl_3$) δ = 7.67 (d, *J* = 8.7 Hz, 2H), 7.55 (d, *J* = 8.8 Hz, 2H), 3.75 (“t”, *J* = 4.8 Hz, 4H), 3.00 (“t”, *J* = 4.8 Hz, 4H), 1.35 (s, 9H).

^{13}C NMR (101 MHz, $CDCl_3$) δ = 157.0, 132.0, 127.9, 126.2, 66.2, 46.1, 35.3, 31.2.

HRMS (ESI$^+$, m/z): calculated for $C_{14}H_{21}NO_3SNa$ $[M+Na]^+$, 306.1134, found 306.1146.

Data is in agreement with that previously reported.[1]

3.2 General Procedure B – Synthesis of Sulfonamides – Electron-rich Anilines

[CAUTION: For safety considerations, the reactions were performed behind a blast shield. No accident, unexpected exothermic event or explosion have ever been observed.]

Round-bottom flask containing the corresponding amine (1.0 equiv.), DABSO (0.60 equiv.), $CuCl_2$ catalyst (5 mol%) was sealed and subjected to three N_2 evacuation/refill cycles before pre-sparged anhydrous MeCN (amine conc. 0.2 M) was added. The resulting solution was placed to cold-water bath (18 °C) and 37% aq. HCl (2.0 equiv.) was added dropwise (slightly exothermic 22 °C to 24 °C). After 10 min, *tert*-butyl nitrite 90 % (1.1 equiv.) was added dropwise (slightly exothermic: 22 °C to 29 °C). After 10 min cold-water bath was removed and rection mixture was heated to 75 °C. After 2 h reaction mixture was cooled down to 0 °C and morpholine (2.2 equiv.) was added dropwise at the same temperature. After addition of morpholine the reaction mixture was warmed to room temperature and stirred for another 30 minutes before being quenched with saturated aqueous solution of NH_4Cl (6.0 mL). The resulting mixture was then extracted with EtOAc (3 x 10 mL). Collected organic phases were dried over anhydrous Na_2SO_4, filtered and concentrated *in vacuo*. The resulting residue was then purified by flash column chromatography with silica gel under standard eluent mixture (typically ethyl acetate [EtOAc] in petroleum ether 40 – 60 °C bp [PE]). The solvents were removed *in vacuo* and the products were finally dried under high vacuum.

4-((4-Methoxyphenyl)sulfonyl)morpholine (3u)

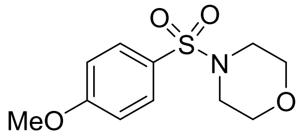

3u

Prepared according to general procedure B, using *p*-anisidine (61.6 mg, 0.50 mmol, 1.0 equiv.), DABSO (72.1 mg, 0.30 mmol, 0.60 equiv.), $CuCl_2$ (3.4 mg, 0.025 mmol, 0.050 equiv.), MECN (2.5 mL), 37% aq. HCl (84 µL, 1.0 mmol, 2.0 equiv.), *tert*-butyl nitrite 90% (73 µL, 0.55 mmol, 1.1 equiv.). Stirred at 75 °C for 2 h, then morpholine (95 µL, 1.1 mmol, 2.2 equiv.). Flash column chromatography (SiO_2, PE:EtOAc = 3:2 to 1:1) afforded the desired sulfonamide **3u** as a pale-orange solid (100 mg, 0.39 mmol, 78%).

^{1}H NMR (400 MHz, $CDCl_3$) δ = 7.73 – 7.64 (m, 2H), 7.05 – 6.96 (m, 2H), 3.87 (s, 3H), 3.73 ("t", *J* = 4.7 Hz, 4H), 2.97 ("t", *J* = 4.7 Hz, 4H).

^{13}C NMR (101 MHz, $CDCl_3$) δ = 163.4, 130.2, 126.8, 114.4, 66.2, 55.8, 46.1.

HRMS (ESI$^+$, m/z): calculated for $C_{11}H_{15}NO_4SNa$ $[M+Na]^+$, 280.0614, found 280.0619.

Data is in agreement with that previously reported.[1]

4-((2-Methoxyphenyl)sulfonyl)morpholine (3v)

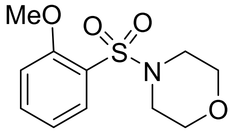

3v

Prepared according to general procedure B, *o*-anisidine (56 µL, 0.50 mmol, 1.0 equiv.), DABSO (72.1 mg, 0.30 mmol, 0.60 equiv.), $CuCl_2$ (3.4 mg, 0.025 mmol, 0.050 equiv.), MeCN (2.5 mL), 37% aq. HCl (84 µL, 1.0 mmol, 2.0 equiv.), *tert*-butyl nitrite 90% (73 µL, 0.55 mmol, 1.1 equiv.), morpholine (95 µL, 1.1 mmol, 2.2 equiv.). Flash column chromatography (SiO_2, PE:EtOAc = 3:2 to 1:1) afforded the desired sulfonamide **3v** as a slightly bronze solid (97 mg, 0.38 mmol, 76%).

^{1}H NMR (400 MHz, $CDCl_3$) δ = 7.89 (dd, *J* = 7.8, 1.7 Hz, 1H), 7.54 (ddd, *J* = 8.3, 7.4, 1.8 Hz, 1H), 7.09 – 6.99 (m, 2H), 3.93 (s, 3H), 3.72 (“t”, *J* = 4.7 Hz, 4H), 3.23 (“t”, *J* = 4.7 Hz, 4H).

^{13}C NMR (101 MHz, $CDCl_3$) δ = 157.1, 134.9, 132.0, 125.9, 120.6, 112.5, 66.8, 56.1, 46.1.

HRMS (ESI$^+$, m/z): calculated for $C_{11}H_{15}NO_4SNa$ $[M+Na]^+$, 280.0614, found 280.0615.

Data is in agreement with that previously reported.[5]

4-((2-Methoxy-5-methylphenyl)sulfonyl)morpholine (3w)

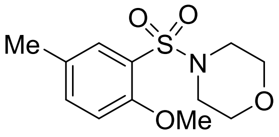

3w

Prepared according to general procedure B, 2-methoxy-5-methylaniline (68.6 mg, 0.50 mmol, 1.0 equiv.), DABSO (72.1 mg, 0.30 mmol, 0.60 equiv.), $CuCl_2$ (3.4 mg, 0.025 mmol, 0.050 equiv.), MeCN (2.5 mL), 37% aq. HCl (84 µL, 1.0 mmol, 2.0 equiv.), *tert*-butyl nitrite 90% (73 µL, 0.55 mmol, 1.1 equiv.). Stirred at 75 °C for 2 h, then morpholine (95 µL, 1.1 mmol, 2.2 equiv.). Flash column chromatography (SiO_2, PE:EtOAc = 3:2 to 1:1) afforded the desired sulfonamide **3w** as a pale-yellow solid (108 mg, 0.40 mmol, 80%).

Mp 87 – 88 °C

^{1}H NMR (400 MHz, $CDCl_3$) δ = 7.68 (dd, J = 2.3, 0.8 Hz, 1H), 7.32 (ddd, J = 8.3, 2.3, 0.8 Hz, 1H), 6.91 (d, J = 8.4 Hz, 1H), 3.89 (s, 3H), 3.72 (“t”, J = 4.7 Hz, 4H), 3.22 (“t”, J = 4.7 Hz, 4H), 2.32 (s, 3H).

^{13}C NMR (101 MHz, $CDCl_3$) δ = 155.0, 135.3, 132.2, 130.2, 125.4, 112.5, 66.9, 56.2, 46.2, 20.5.

HRMS (ESI$^+$, m/z): calculated for $C_{12}H_{17}NO_4SK$ $[M+K]^+$, 310.0510, found 310.0512.

IR (ν_{max}, cm^{-1}) 3056, 2963, 2861, 1497, 1352, 1283, 1259, 1165, 956, 730, 618.

4-((2,3-Dihydrobenzo[*b*][1,4]dioxin-6-yl)sulfonyl)morpholine (3x)

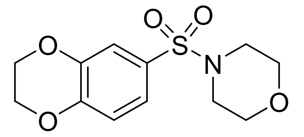

3x

Prepared according to general procedure B, using 1,4-benzodioxan-6-amine (61 µL, 0.50 mmol, 1.0 equiv.), DABSO (72.1 mg, 0.30 mmol, 0.60 equiv.), $CuCl_2$ (3.4 mg, 0.025 mmol, 0.050 equiv.), MeCN (2.5 mL), 37% aq. HCl (84 µL, 1.0 mmol, 2.0 equiv.), *tert*-butyl nitrite 90% (73 µL, 0.55 mmol, 1.1 equiv.). Stirred at 75 °C for 1 h, then morpholine (95 µL, 1.1 mmol, 2.2 equiv.). Flash column chromatography (SiO_2, PE:EtOAc = 7:3) afforded the desired sulfonamide **3x** as a pale-orange solid (93 mg, 0.33 mmol, 65%).

^{1}H NMR (400 MHz, $CDCl_3$) δ = 7.27 (d, *J* = 2.2 Hz, 1H), 7.24 (dd, *J* = 8.4, 2.2 Hz, 1H), 6.98 (d, *J* = 8.4 Hz, 1H), 4.37 – 4.27 (m, 4H), 3.74 (“t”, *J* = 4.8 Hz, 4H), 2.98 (“t”, *J* = 4.8 Hz, 4H).

^{13}C NMR (101 MHz, $CDCl_3$) δ = 147.8, 143.7, 127.5, 121.7, 117.9, 117.5, 66.2, 64.7, 64.3, 46.2.

HRMS (ESI$^+$, m/z): calculated for $C_{12}H_{15}NO_5SNa$ $[M+Na]^+$, 308.0563, found 308.0567.

Data is in agreement with that previously reported.[1]

4-(Benzo[*d*][1,3]dioxol-5-ylsulfonyl)morpholine (3y)

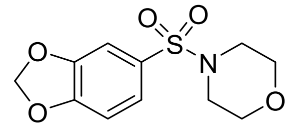

3y

Prepared according to general procedure B, using 1,3-benzodioxol-5-amine (68.6 mg, 0.50 mmol, 1.0 equiv.), DABSO (72.1 mg, 0.30 mmol, 0.60 equiv.), $CuCl_2$ (3.4 mg, 0.025 mmol, 0.050 equiv.), MeCN (2.5 mL), 37% aq. HCl (84 µL, 1.0 mmol, 2.0 equiv.), *tert*-butyl nitrite 90% (73 µL, 0.55 mmol, 1.1 equiv.). Stirred at 75 °C for 1 h, then morpholine (95 µL, 1.1 mmol, 2.2 equiv.). Flash column chromatography (SiO_2, PE:EtOAc = 4:1 to 7:3) afforded the desired sulfonamide **3y** as an orange solid (75 mg, 0.28 mmol, 56%).

^{1}H NMR (400 MHz, $CDCl_3$) δ = 7.31 (dd, *J* = 8.2, 1.8 Hz, 1H), 7.17 (d, *J* = 1.8 Hz, 1H), 6.92 (d, *J* = 8.2 Hz, 1H), 6.10 (s, 2H), 3.74 (“t”, *J* = 4.7 Hz, 4H), 2.99 (“t”, *J* = 4.7 Hz, 4H).

^{13}C NMR (101 MHz, $CDCl_3$) δ = 151.8, 148.4, 128.4, 123.6, 108.5, 108.1, 102.5, 66.2, 46.1.

HRMS (ESI$^+$, m/z): calculated for $C_{11}H_{13}NO_5SK$ $[M+K]^+$, 310.0146, found 310.0150.

Data is in agreement with that previously reported.[1]

4-((4-(Methylthio)phenyl)sulfonyl)morpholine (3z)

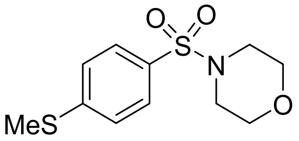

3z

Prepared according to general procedure B, using 4-(methylthio)aniline (62 µL, 0.50 mmol, 1.0 equiv.), DABSO (72.1 mg, 0.30 mmol, 0.60 equiv.), $CuCl_2$ (3.4 mg, 0.025 mmol, 0.050 equiv.), MeCN (2.5 mL), 37% aq. HCl (84 µL, 1.0 mmol, 2.0 equiv.), *tert*-butyl nitrite 90% (73 µL, 0.55 mmol, 1.1 equiv.). Stirred at 75 °C for 1 h, then morpholine (95 µL, 1.1 mmol, 2.2 equiv.). Flash column chromatography (SiO_2, PE:EtOAc = 4:1 to 7:3) afforded the desired sulfonamide **3z** as a white solid (109 mg, 0.40 mmol, 80%).

^{1}H NMR (400 MHz, $CDCl_3$) δ = 7.67 – 7.59 (m, 2H), 7.37 – 7.29 (m, 2H), 3.73 ("t", *J* = 4.8 Hz, 4H), 2.98 ("t", *J* = 4.8 Hz, 4H), 2.53 (s, 3H).

^{13}C NMR (101 MHz, $CDCl_3$) δ = 146.4, 130.9, 128.3, 125.5, 66.2, 46.1, 14.9.

HRMS (ESI$^+$, m/z): calculated for $C_{11}H_{15}NO_3S_2Na$ $[M+Na]^+$, 296.0386, found 296.0390.

Data is in agreement with that previously reported.[2]

3.3 General Procedure C – Synthesis of Sulfonamides – Heterocyclic Anilines

Round-bottom flask containing the corresponding amine (1.0 equiv.), DABSO (0.60 equiv.), $CuCl_2$ catalyst (5 mol%) was sealed and subjected to three N_2 evacuation/refill cycles before pre-sparged anhydrous MeCN (amine conc. 0.2 M) was added. The resulting solution was placed to cold-water bath (18 °C) and 37% aq. HCl (1.2 equiv.) was added dropwise (slightly exothermic 22 °C to 24 °C). After 10 min, *tert*-butyl nitrite 90% (1.1 equiv.) was added dropwise (slightly exothermic: 22 °C to 29 °C). After 10 min cold-water bath was removed and rection mixture was stirred overnight. After 17 h reaction mixture was cooled down to 0 °C and morpholine (2.2 equiv.) was added dropwise at the same temperature. After addition of morpholine the reaction mixture was warmed to room temperature and stirred for another 30 minutes before being quenched with saturated aqueous solution of NH_4Cl (6.0 mL). The resulting mixture was then extracted with EtOAc (3 x 10 mL). Collected organic phases were dried over anhydrous Na_2SO_4, filtered and concentrated *in vacuo*. The resulting residue was then purified by flash column chromatography with silica gel under standard eluent mixture (typically ethyl acetate [EtOAc] in petroleum ether 40 – 60 °C bp [PE]). The solvents were removed *in vacuo* and the products were finally dried under high vacuum.

4-((2-Chloropyridin-3-yl)sulfonyl)morpholine (3aa)

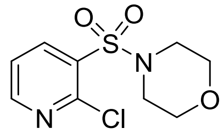

3aa

Prepared according to general procedure C, using 2-chloropyridin-3-amine (64.3 mg, 0.50 mmol, 1.0 equiv.), DABSO (72.1 mg, 0.30 mmol, 0.60 equiv.), $CuCl_2$ (3.4 mg, 0.025 mmol, 0.050 equiv.), MeCN (2.5 mL), 37% aq. HCl (50 µL, 0.60 mmol, 1.2 equiv.), *tert*-butyl nitrite 90% (73 µL, 0.55 mmol, 1.1 equiv.). Stirred at rt for 17 h, then morpholine (95 µL, 1.1 mmol, 2.2 equiv.). Flash column chromatography (SiO_2, PE:EtOAc = 3:2) the desired sulfonamide **3aa** as an orange solid (68 mg, 0.26 mmol, 52%).

Mp 107 – 108 °C.

^{1}H NMR (400 MHz, $CDCl_3$) δ = 8.58 (dd, *J* = 4.8, 1.9 Hz, 1H), 8.38 (dd, *J* = 7.8, 1.9 Hz, 1H), 7.43 (dd, *J* = 7.8, 4.8 Hz, 1H), 3.74 (“t”, *J* = 4.8 Hz, 4H), 3.35 (“t”, *J* = 4.8 Hz, 4H).

^{13}C NMR (101 MHz, $CDCl_3$) δ = 152.8, 148.6, 141.1, 133.8, 122.7, 66.6, 46.1.

HRMS (ESI$^+$, m/z): calculated for $C_9H_{11}ClN_2O_3SNa$ $[M+Na]^+$, 285.0071, found 285.0066.

IR (ν_{max}, cm^{-1}) 3128, 3070, 2967, 2918, 2861, 1617, 1394, 1350, 1299, 1245, 1169, 1115, 954, 781, 739, 653.

4-((2-Methoxypyridin-3-yl)sulfonyl)morpholine (3ab)

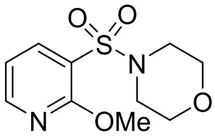

3ab

Prepared according to general procedure C, using 2-methoxypyridin-3-amine (62.1 mg, 0.50 mmol, 1.0 equiv.), DABSO (72.1 mg, 0.30 mmol, 0.60 equiv.), $CuCl_2$ (3.4 mg, 0.025 mmol, 0.050 equiv.), MeCN (2.5 mL), 37% aq. HCl (50 µL, 0.60 mmol, 1.2 equiv.), *tert*-butyl nitrite 90% (73 µL, 0.55 mmol, 1.1 equiv.). Stirred at rt for 17 h, then morpholine (95 µL, 1.1 mmol, 2.2 equiv.). Flash column chromatography (SiO_2, PE:EtOAc = 3:2 to 1:1) the desired sulfonamide **3ab** as an orange solid (72 mg, 0.28 mmol, 56%).

Mp 116 – 117 °C.

^{1}H NMR (400 MHz, $CDCl_3$) δ = 8.33 (dd, *J* = 4.9, 1.9 Hz, 1H), 8.16 (dd, *J* = 7.6, 1.9 Hz, 1H), 7.01 (dd, *J* = 7.6, 4.9 Hz, 1H), 4.06 (s, 3H), 3.72 ("t", *J* = 4.7 Hz, 4H), 3.27 ("t", *J* = 4.7 Hz, 4H).

^{13}C NMR (101 MHz, $CDCl_3$) δ = 160.1, 151.6, 141.1, 121.2, 116.8, 66.8, 54.3, 46.2.

HRMS (ESI$^+$, m/z): calculated for $C_{10}H_{14}N_2O_4SNa$ $[M+Na]^+$, 281.0567, found 281.0578.

IR (ν_{max}, cm^{-1}) 3079, 2958, 2859, 1583, 1472, 1405, 1348, 1242, 1170, 1115, 1073, 1009, 958, 839, 770, 730, 618.

Ethyl 5-(morpholinosulfonyl)benzofuran-2-carboxylate (3ac)

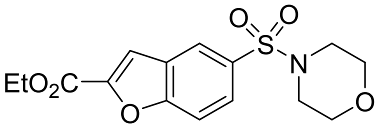

3ac

Prepared according to general procedure C, using ethyl 5-aminobenzofuran-2-carboxylate (102.6 mg, 0.50 mmol, 1.0 equiv.), DABSO (72.1 mg, 0.30 mmol, 0.60 equiv.), $CuCl_2$ (3.4 mg, 0.025 mmol, 0.050 equiv.), MeCN (2.5 mL), 37% aq. HCl (84 µL, 1.0 mmol, 2.0 equiv.), *tert*-butyl nitrite 90% (73 µL, 0.55 mmol, 1.1 equiv.). Stirred at rt for 17 h, then morpholine (95 µL, 1.1 mmol, 2.2 equiv.). Flash column chromatography (SiO_2, PE:EtOAc = 7:3) the desired sulfonamide **3ac** as a slightly pink solid (108 mg, 0.32 mmol, 64%).

Mp 157 – 158 °C.

^{1}H NMR (400 MHz, $CDCl_3$) δ = 8.15 (dd, *J* = 1.8, 0.7 Hz, 1H), 7.83 (dd, *J* = 8.8, 1.9 Hz, 1H), 7.74 ("td", *J* = 8.8, 0.9 Hz, 1H), 7.60 (d, *J* = 0.9 Hz, 1H), 4.47 (q, *J* = 7.1 Hz, 2H), 3.74 ("t", *J* = 4.7 Hz, 4H), 3.02 ("t", *J* = 4.7 Hz, 4H), 1.44 (t, *J* = 7.1 Hz, 3H).

^{13}C NMR (101 MHz, $CDCl_3$) δ = 158.9, 157.4, 148.1, 131.3, 127.5, 126.8, 123.9, 113.6, 113.3, 66.2, 62.2, 46.2, 14.4.

HRMS (ESI$^+$, m/z): calculated for $C_{15}H_{17}NO_6SNa$ $[M+Na]^+$, 362.0669, found 362.0669.

IR (ν_{max}, cm^{-1}) 3100, 2984, 2906, 2861, 1733, 1586, 1357, 1300, 1264, 1158, 1188, 1158, 1114, 1077, 919, 740, 638.

Methyl 3-(morpholinosulfonyl)thiophene-2-carboxylate (3ad)

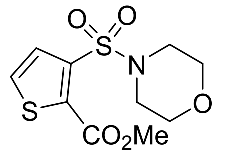

3ad

Prepared according to general procedure C, using methyl 3-aminothiophene-2-carboxylate (78.6 mg, 0.50 mmol, 1.0 equiv.), DABSO (72.1 mg, 0.30 mmol, 0.60 equiv.), $CuCl_2$ (3.4 mg, 0.025 mmol, 0.050 equiv.), MeCN (2.5 mL), 37% aq. HCl (50 µL, 0.60 mmol, 1.2 equiv.), *tert*-butyl nitrite 90% (73 µL, 0.55 mmol, 1.1 equiv.). Stirred at rt for 17 h, then morpholine (95 µL, 1.1 mmol, 2.2 equiv.). Flash column chromatography (SiO_2, PE:EtOAc = 4:1 to 7:3) the desired sulfonamide **3ad** as an yellow solid (58 mg, 0.20 mmol, 40%).

^{1}H NMR (400 MHz, $CDCl_3$) δ = 7.51 (d, *J* = 5.2 Hz, 1H), 7.45 (d, *J* = 5.3 Hz, 1H), 3.91 (s, 3H), 3.73 ("t", *J* = 4.7 Hz, 4H), 3.31 ("t", *J* = 4.7 Hz, 4H).

^{13}C NMR (101 MHz, $CDCl_3$) δ = 160.0, 140.1, 134.3, 131.4, 129.3, 66.6, 53.2, 46.3.

HRMS (ESI$^+$, m/z): calculated for $C_{10}H_{13}NO_5S_2Na$ $[M+Na]^+$, 314.0127, found 314.0125.

Data is in agreement with that previously reported.[4]

3.4 Synthesis of Sulfonylchloride – 4-Methoxybenzenesulfonyl chloride (3u')

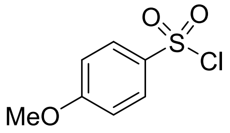

3u'

Round-bottom flask containing *p*-anisidine (61.6 mg, 0.50 mmol, 1.0 equiv.), DABSO (72.1 mg, 0.30 mmol, 0.60 equiv.), $CuCl_2$ (3.4 mg, 0.025 mmol, 0.050 equiv.) was sealed and subjected to three N_2 evacuation/refill cycles before pre-sparged anhydrous MeCN (2.5 mL) was added. The resulting suspension was placed to cold-water bath (18 °C) and 37% aq. HCl (84 µL, 1.0 mmol, 2.0 equiv.) was added dropwise followed by *tert*-butyl nitrite 90% (73 µL, 0.55 mmol, 1.1 equiv.) (slightly exothermic 22 °C to 24 °C) (10 min break between addition of 37% aq. HCl and *tert*-butyl nitrite). After 10 min cold-water bath was removed and rection mixture was heated to 75 °C. After 2 h reaction mixture was cooled down to 0 °C and quenched with saturated aqueous solution of NH_4Cl (6.0 mL). The resulting mixture was then extracted with DCM (3 x 10 mL). Collected organic phases were dried over anhydrous Na_2SO_4, filtered and concentrated *in vacuo*. The resulting residue was purified by flash column chromatography (SiO_2, PE:EtOAc = 9:1 to 4:1) providing the desired sulfonyl chloride **3u'** as an orange oil (81 mg, 0.39 mmol, 79%).

^{1}H NMR (400 MHz, $CDCl_3$) δ = 8.03 – 7.93 (m, 2H), 7.09 – 7.00 (m, 2H), 3.92 (s, 3H).

^{13}C NMR (101 MHz, $CDCl_3$) δ = 165.0, 136.3, 129.7, 114.9, 56.1.

HRMS (ESI$^+$, m/z): calculated for $C_8H_{11}O_4S$ $[M+H]^+$, 203.0373, found 203.0365. MS confirmed sulfonate ester (MeOH), sulfonyl chloride confirmed by GC-MS (see page S67).

Data is in agreement with that previously reported.[8]

3.5 Scale-up – 4-((4-Chlorophenyl)sulfonyl)morpholine (3b)

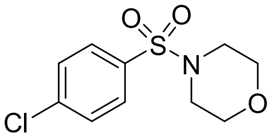

3b

Prepared according to general procedure A, using 4-chloroaniline (0.638 g, 5.0 mmol, 1.0 equiv.), DABSO (0.721 g, 3.0 mmol, 0.60 equiv.), $CuCl_2$ (33.6 mg, 0.25 mmol, 0.050 equiv.), MeCN (25.0 mL), 37% aq. HCl (0.836 mL, 10.0 mmol, 2.0 equiv.), *tert*-butyl nitrite 90% (0.727 mL, 5.5 mmol, 1.1 equiv.). Stirred at rt for 17 h, then morpholine (0.950 mL, 1.1 mmol, 2.2 equiv.). Flash column chromatography (SiO_2, PE:EtOAc = 4:1 to 7:3) afforded the desired sulfonamide **3b** as an orange solid (910 mg, 3.49 mmol, 70%).

^{1}H NMR (400 MHz, $CDCl_3$) δ = δ 7.73 – 7.64 (m, 2H), 7.57 – 7.49 (m, 2H), 3.74 (“t”, *J* = 4.8 Hz, 4H), 3.00 (“t”, *J* = 4.8 Hz, 4H).

^{13}C NMR (101 MHz, $CDCl_3$) δ = 139.9, 133.9, 129.6, 129.4, 66.2, 46.1.

HRMS (ESI$^+$, m/z): HRMS (ESI$^+$, m/z): calculated for $C_{10}H_{12}ClNO_3SH$ $[M+H]^+$, 262.0299, found 262.0302. Data is in agreement with that previously reported.[2]

3.6 Scale-up – 2-fluoropyridine-3-sulfonyl chloride (1)

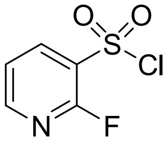

1

Under N_2-atmosphere, a 1 L sulfonation flask with Teflon overhead stirrer was charged with 2-fluoropyridin-3-amine (**2**, 20.0 g, 178.4 mmol, 1.0 equiv.), DABSO (25.7 g, 107.0 mmol, 0.60 equiv.), $CuCl_2$ (600 mg, 4.46 mmol, 0.025 equiv.) and MeCN (200 mL, 10.0 vol.). The flask was placed into an ambient water-bath and 32% aq. HCl (35 mL, 356.8 mmol, 2.0 equiv.) was added. Then, *tert*-butyl nitrite 90% (25.9 mL, 196.2 mmol, 1.1 equiv.) was added dropwise via syringe pump over 40 min, keeping the internal temperature below 30 °C. After 15 min at rt, LC/MS confirmed full conversion of **2**. CPME (200 mL, 10.0 vol.) was added, followed by addition of a cold solution of sulfamic acid (3.46 g) in H_2O (200 mL, 10.0 vol.). The biphasic solution was transferred into a separatory funnel and the phases separated. The organic phase was washed twice with cold H_2O (2 x 100 mL, 2 x 5.0 vol.) and then concentrated *in vacuo* to obtain a brown crude oil. The residue was purified by Kugelrohr

distillation (125 °C, ca. 0.1 mBar) to obtain the pure product (95% a/a by LC/MS) as an orange oil (28.0 g, 143.1 mmol, 80%).

^{1}H NMR (400 MHz, $CDCl_3$) δ 8.59 (dt, *J* = 4.9, 1.4 Hz, 1H), 8.41 (ddd, *J* = 9.5, 7.8, 1.9 Hz, 1H), 7.49 (ddd, *J* = 7.8, 4.9, 1.3 Hz, 1H).

^{13}C NMR (101 MHz, $CDCl_3$) δ 157.9 (d, *J* = 249.5 Hz), 154.6 (d, *J* = 15.2 Hz), 140.5, 127.8 (d, *J* = 28.9 Hz), 122.3 (d, *J* = 5.2 Hz).

^{19}F NMR (377 MHz, $CDCl_3$) δ -57.66 (s).

IR (ν_{max}, cm^{-1}) 1588, 1572, 1459, 1431, 1388, 1346, 1298, 1228, 1182, 859, 750, 694.

3.7 Control Experiment with pre-formed diazonium salt

General procedure B was performed in a two-step protocol, with the diazonium salt formed *in situ* before addition to a stirred solution of DABSO and $CuCl_2$ in MeCN, with detailed procedure and spectroscopic data below. [CAUTION: reaction performed behind a blast shield]

A microwave vial containing *p*-anisidine (61.6 mg, 0.50 mmol, 1.0 equiv.) was sealed and subjected to three N_2-evacuation/refill cycles before pre-sparged anhydrous MeCN (1.25 mL, amine conc. 0.4 M) was added (picture 1). The resulting solution was placed in a cold-water bath (18 °C) under a N_2 atmosphere and 37% aq. HCl (84 µL, 1.0 mmol, 2.0 equiv.) was added dropwise. After 10 min (picture 2), *tert*-butyl nitrite 90% (73 µL, 0.55 mmol, 1.1 equiv.) was added dropwise. After 10 min (picture 3), the resultant solution was added dropwise to a pre-formed suspension of DABSO (72.1 mg, 0.30 mmol, 0.60 equiv.) and $CuCl_2$ (3.4 mg, 0.025 mmol, 0.050 equiv.) in pre-sparged anhydrous MeCN (1.25 mL, amine conc. 0.4 M) (picture 4). The reaction mixture was heated to 75 °C (picture 5). After 2 h the reaction mixture was cooled down to 0 °C (picture 6) and quenched with saturated aqueous solution of NH_4Cl (6.0 mL). The resulting mixture was then extracted with CH_2Cl_2 (3 x 10 mL). Collected organic phases were dried over anhydrous Na_2SO_4, filtered and concentrated *in vacuo*. The NMR yield for sulfonyl chloride **3u’** (83%) was determined by quantitative ^{1}H NMR spectroscopy using dibromomethane (35 µL, 0.50 mmol, 1.0 equiv.) as an internal standard. The integration of the CH_2Br_2 signal at 4.93 ppm (s, 2H) was compared to the signal of **3u’** at 8.00 – 7.94 (m, 2H), 7.07 – 7.01 (m, 2H).

1

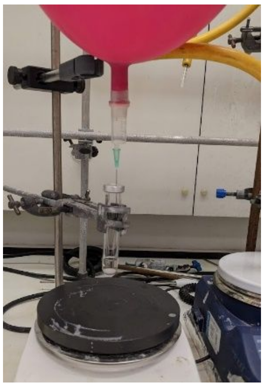

2

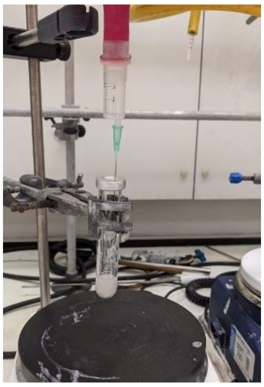

3

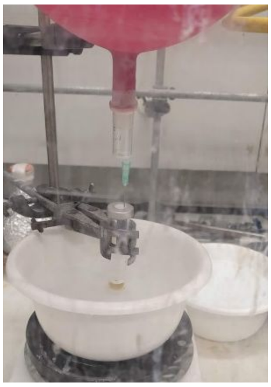

4

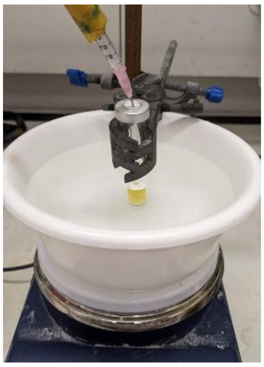

5

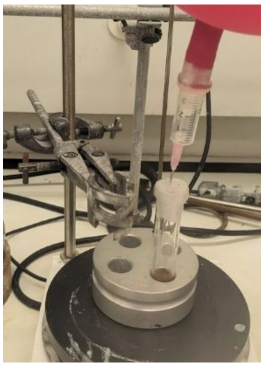

6

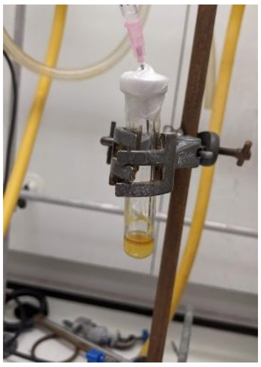

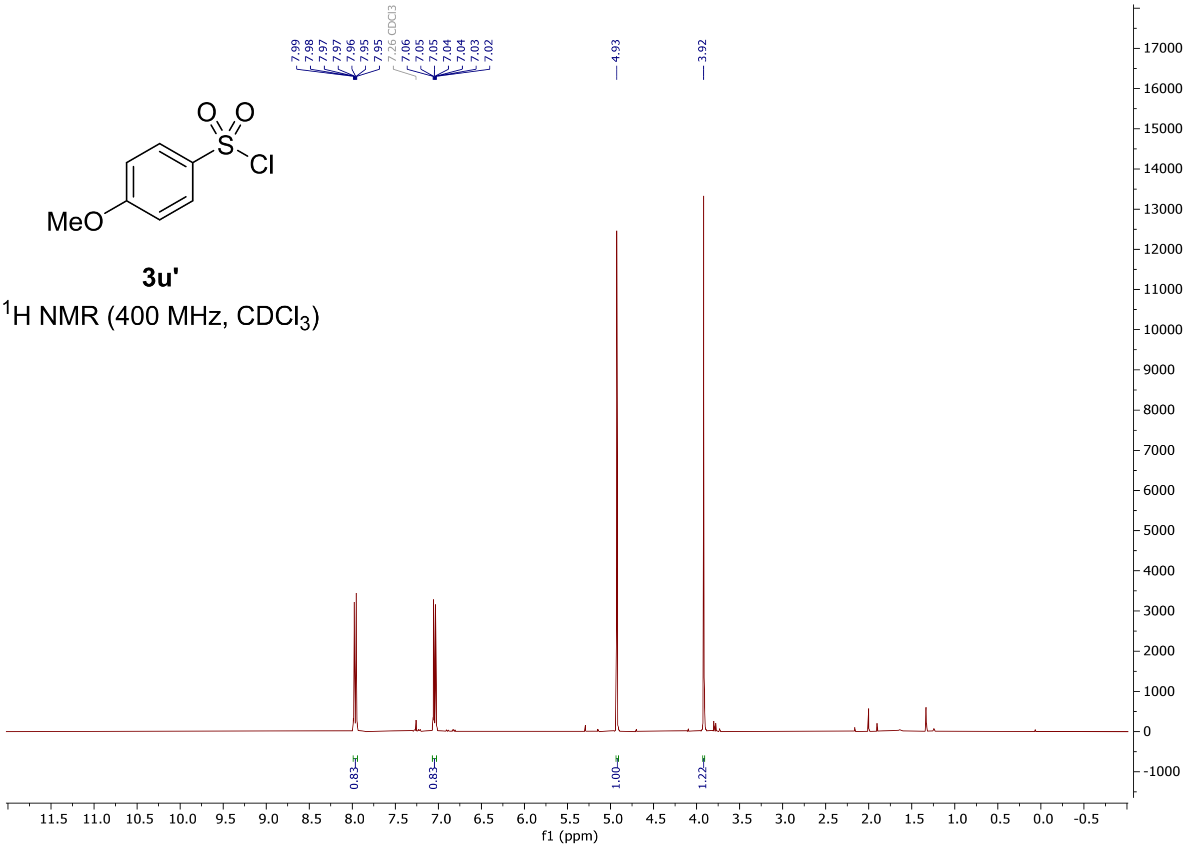

3u'

^{1}H NMR (400 MHz, $CDCl_3$)

Monitoring of the reaction progression was performed by TLC, with full consumption of *p*-anisidine to the corresponding diazonium salt observed within 20 min (confirmed by HRMS and IR, see below). The formation of 4-methoxybenzenediazonium chloride was confirmed by mass spectrometry and IR spectroscopy.

Analytical data for intermediate diazonium salt:

HRMS (ESI$^+$, m/z): calculated for $C_7H_7N_2O^+$ [M], 135.0553, found 135.0551.

IR (ν_{max}, cm^{-1}): 3374, 3099, 2244 ($N{\equiv}N^+$), 1583, 1493, 1249, 1007, 844.

4. Process Safety Measurements

4.1 RC-1 Run

The reaction was performed on 12.0 g scale of **2**, as described above in chapter 3.6. *tert*-Butyl nitrite was dosed over 30 min at IT = 20 °C. Reaction was fully dose-controlled.

1st exotherm: *tert*-butyl nitrite addition (24.85 kJ)

2nd exotherm: sulfamic acid in H_2O addition (3.35 kJ)

RC1mx	Description
Device	RC1mx
Reactor	AP01-0.5-RTCal-3w
Stirrer	Overhead (Pitched-blade up, C22, Ø 38mm / Standard shaft, C22, Length 400mm)
ECB	Gravimetric Dosings:<br>- Substance *t*-Bu-ONO (ProMinent, Frequency3Hz)<br>- Sulfamic acid in water (ProMinent, Frequency3Hz)
Other	Tr Sensor, pH Sensor<br>Calibration Heater

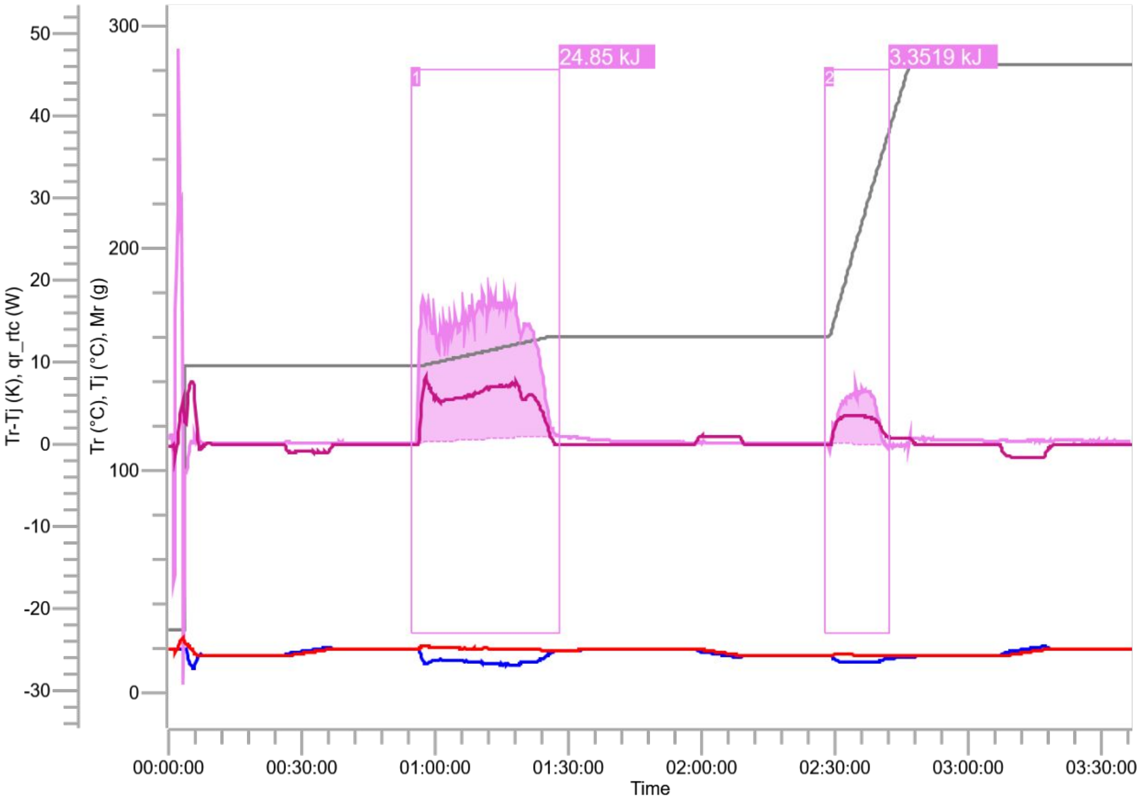

Trend	Color	Units
Tr		°C
Tj		°C
Tr-Tj		K
qr_rtc		W
Mr		g

4.2 DSC Measurements

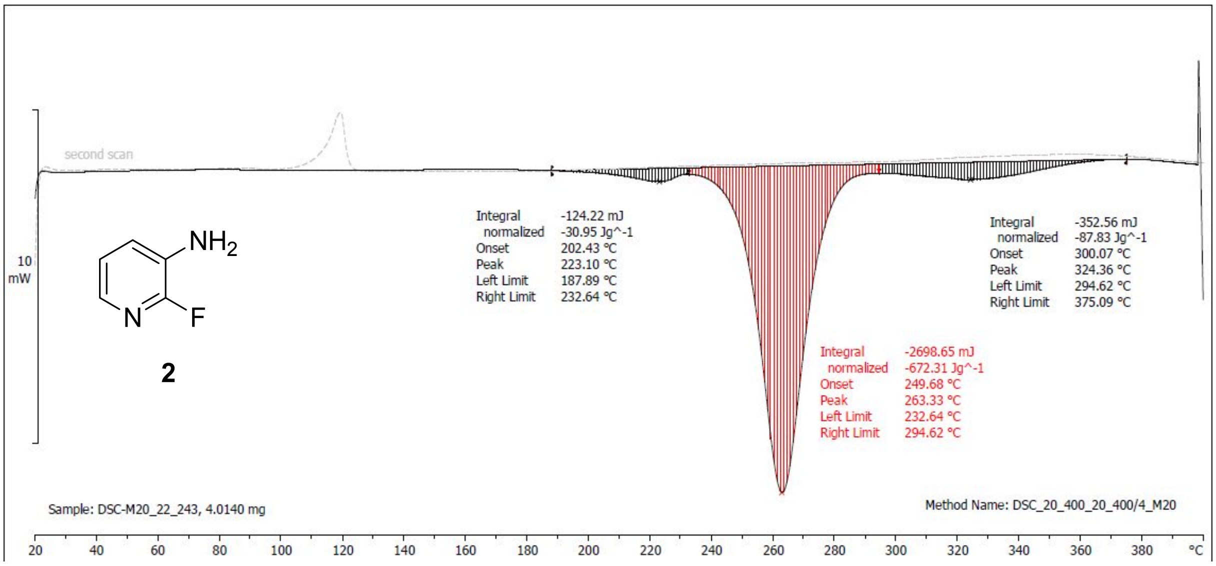

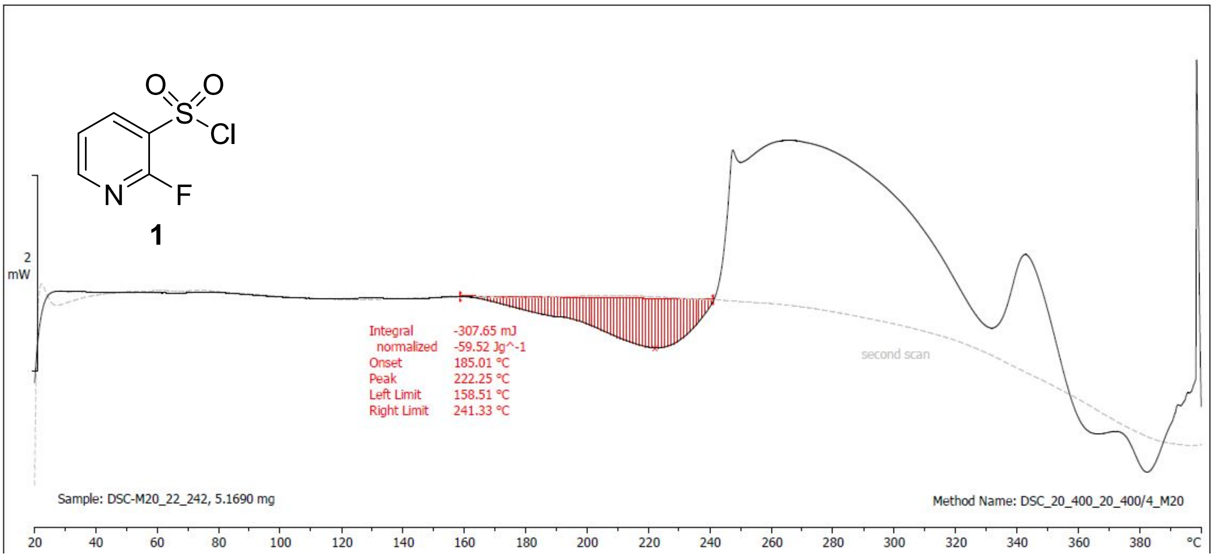

5. NMR spectra

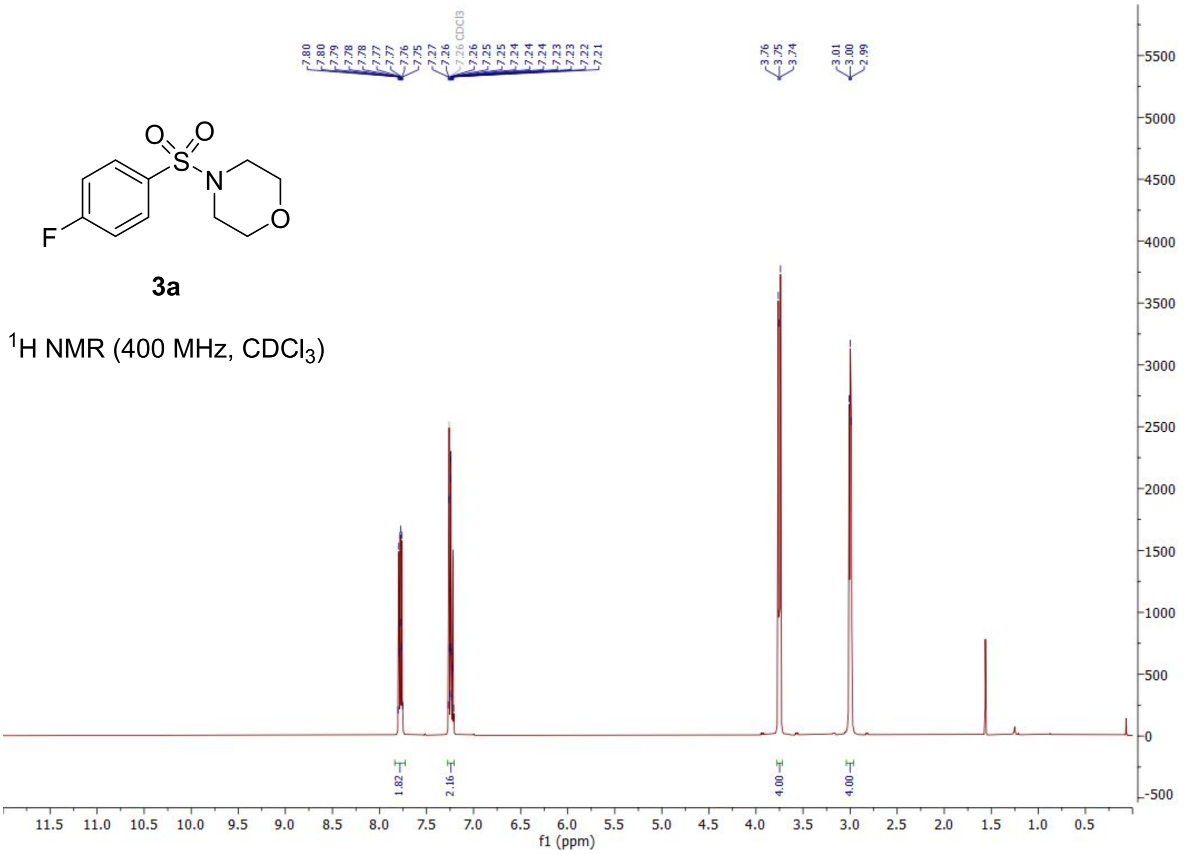

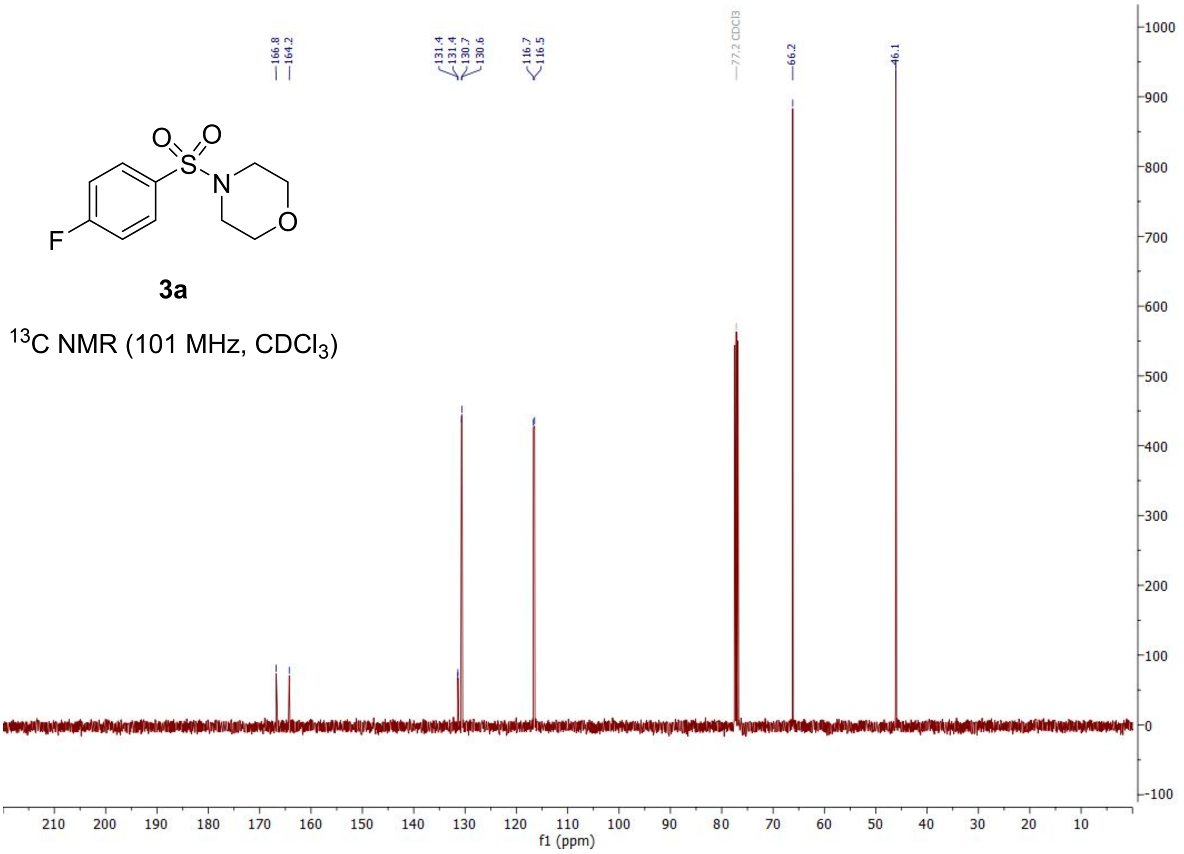

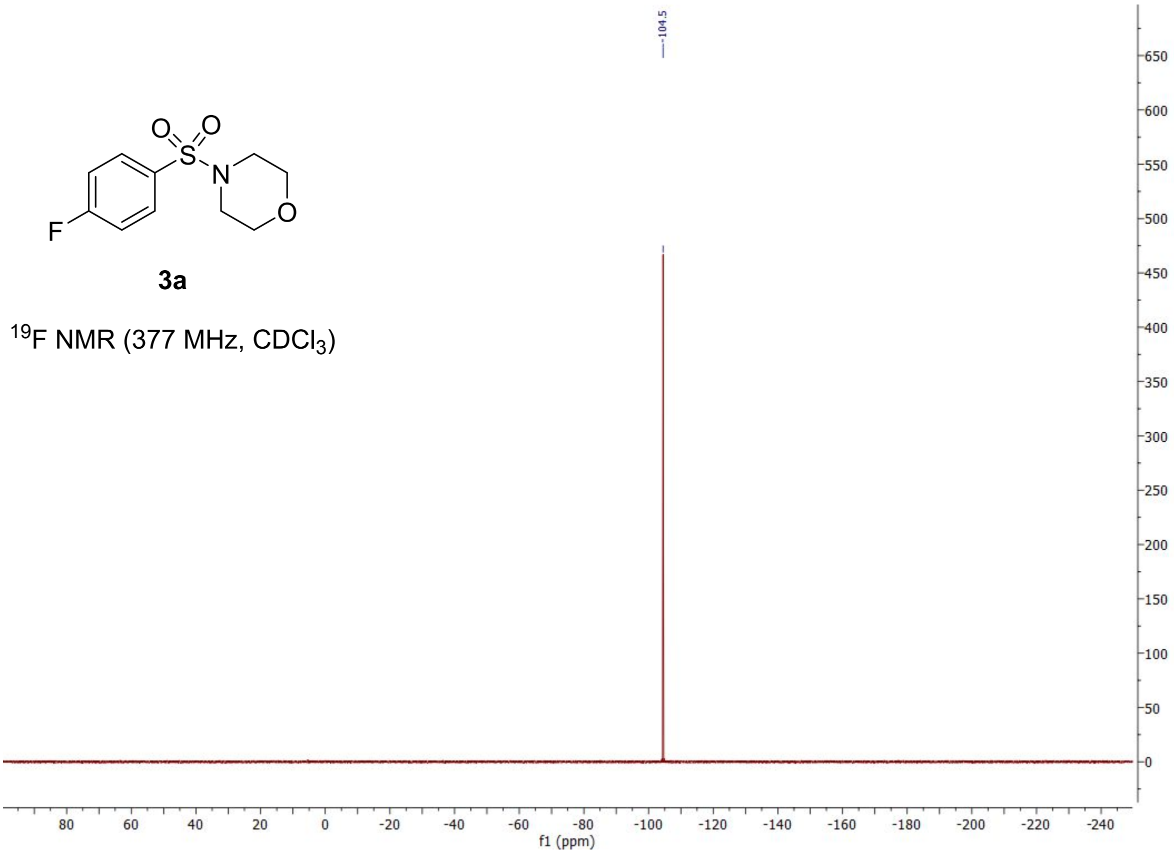

3a

^{19}F NMR (377 MHz, $CDCl_3$)

3b

^{1}H NMR (400 MHz, $CDCl_3$)

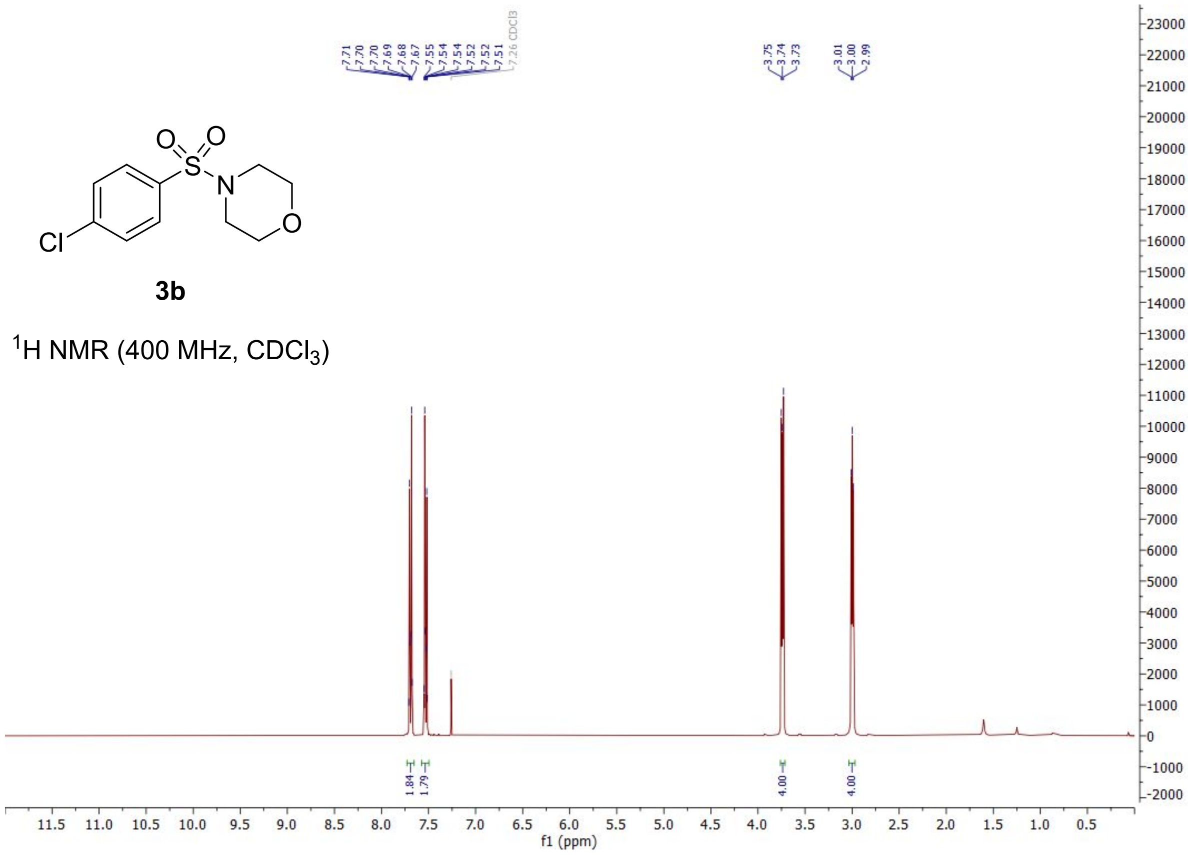

3b

^{13}C NMR (101 MHz, $CDCl_3$)

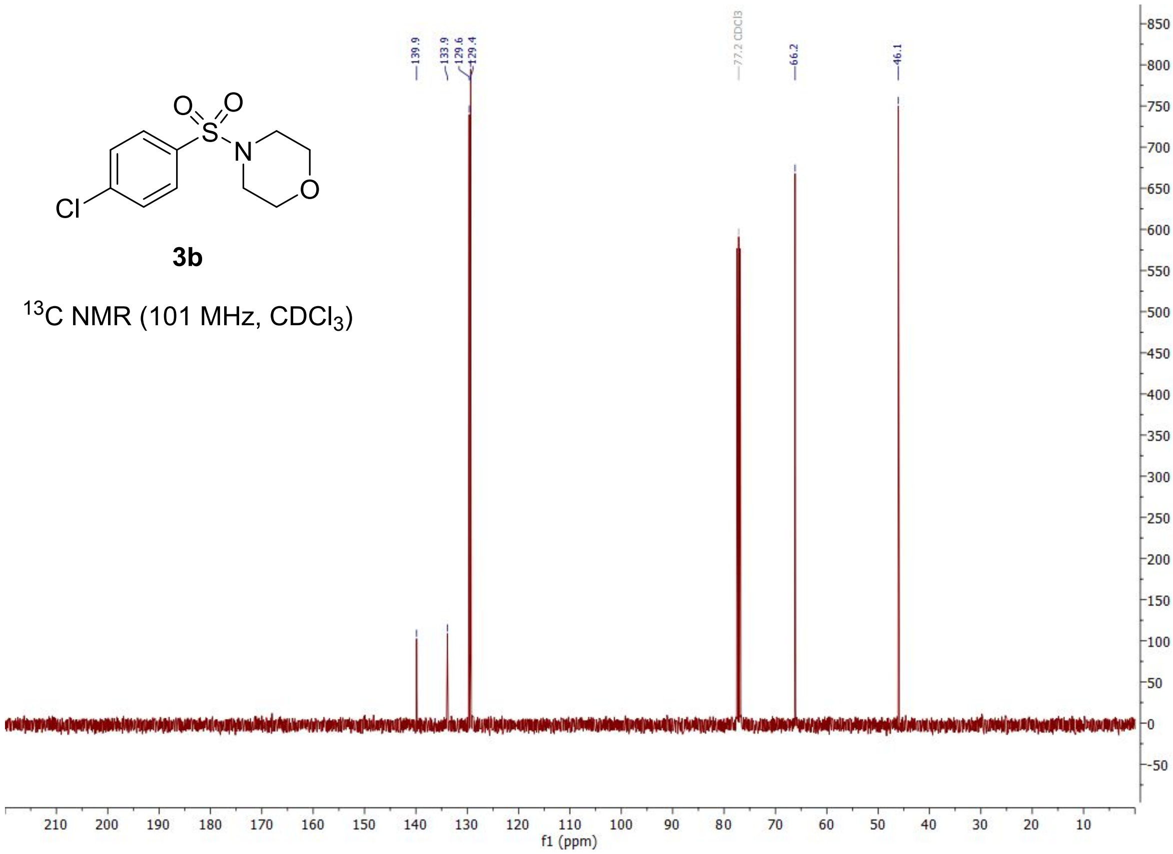

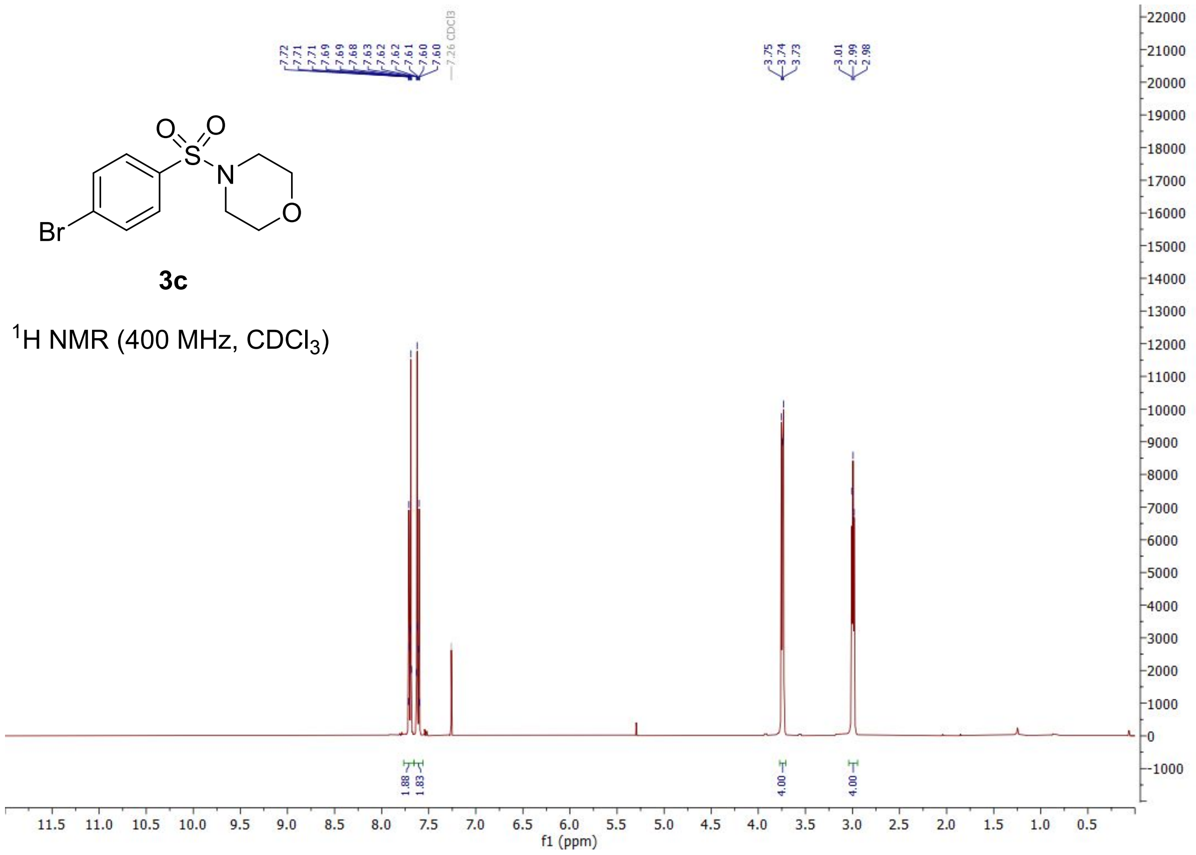
7.72
7.71
7.71
7.69
7.69
7.68
7.63
7.62
7.62
7.61
7.60
7.60
7.26 CDCl3
3.75
3.74
3.73
3.01
2.99
2.98
Br
O
S
O
N
O
3c
^{1}H NMR (400 MHz, CDCl$_3$)
1.88
1.83
4.00
4.00
f1 (ppm)

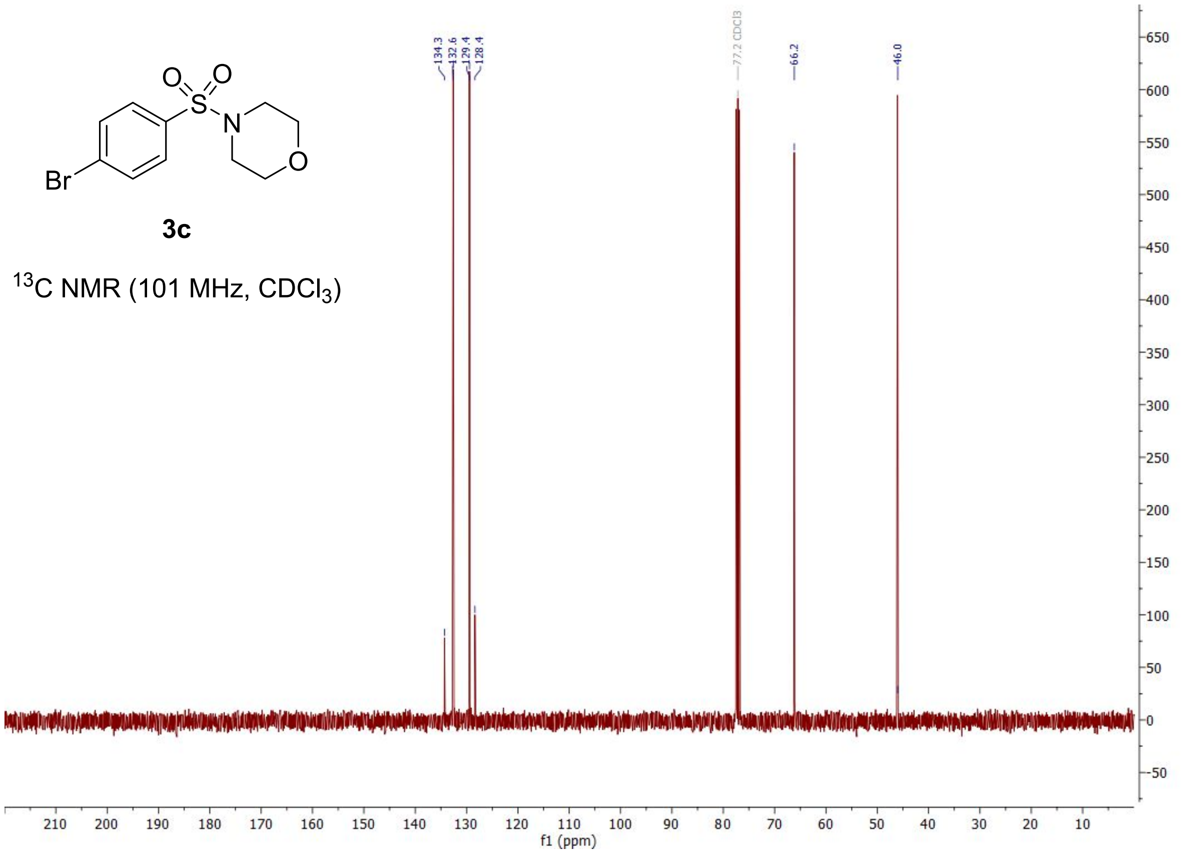
134.3
132.6
129.4
128.4
77.2 CDCl3
66.2
46.0
Br
O
S
O
N
O
3c
^{13}C NMR (101 MHz, CDCl$_3$)
f1 (ppm)

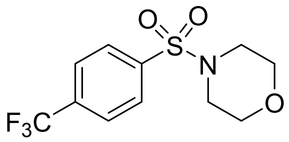

3d

^1H NMR (400 MHz, $CDCl_3$)

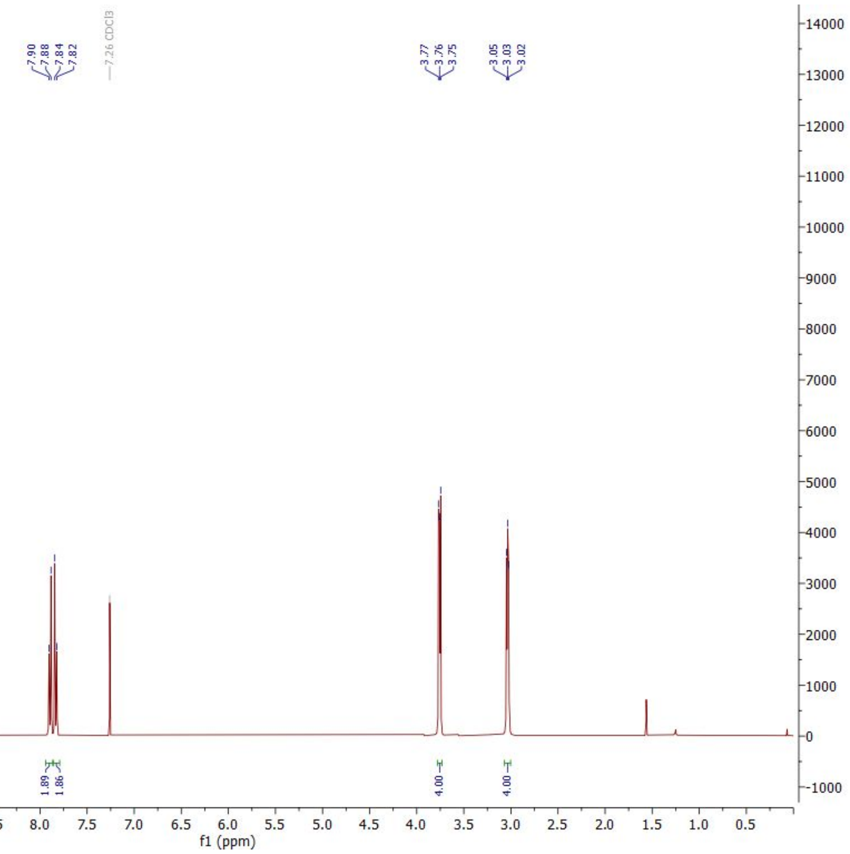

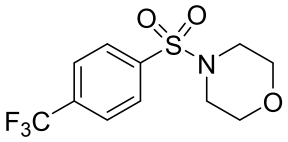

3d

^{13}C NMR (101 MHz, $CDCl_3$)

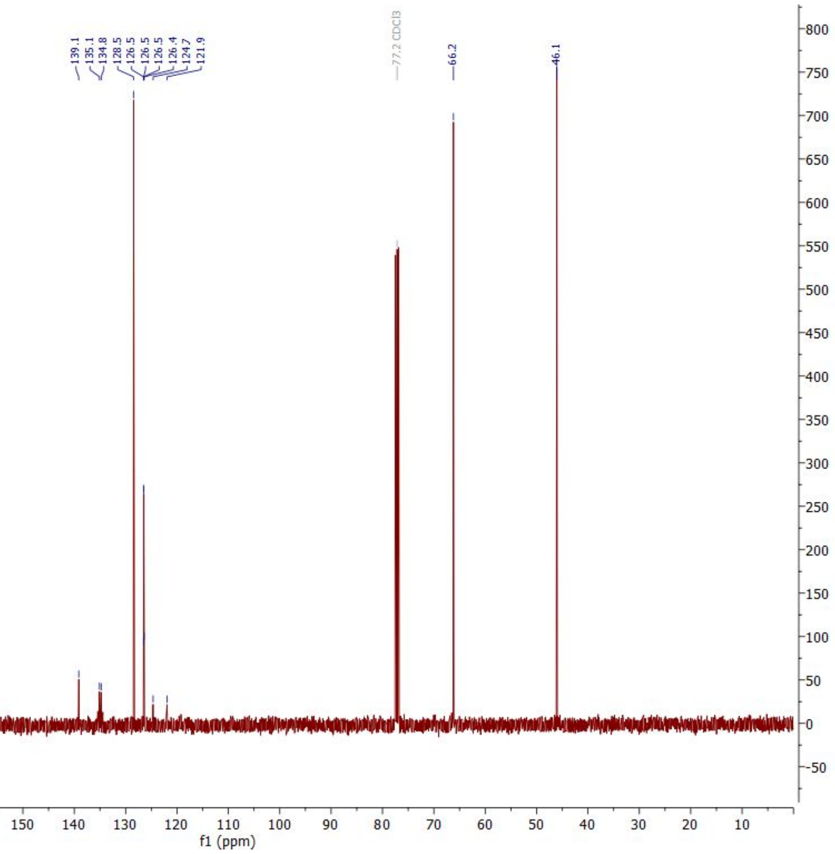

3d

^{19}F NMR (377 MHz, $CDCl_3$)

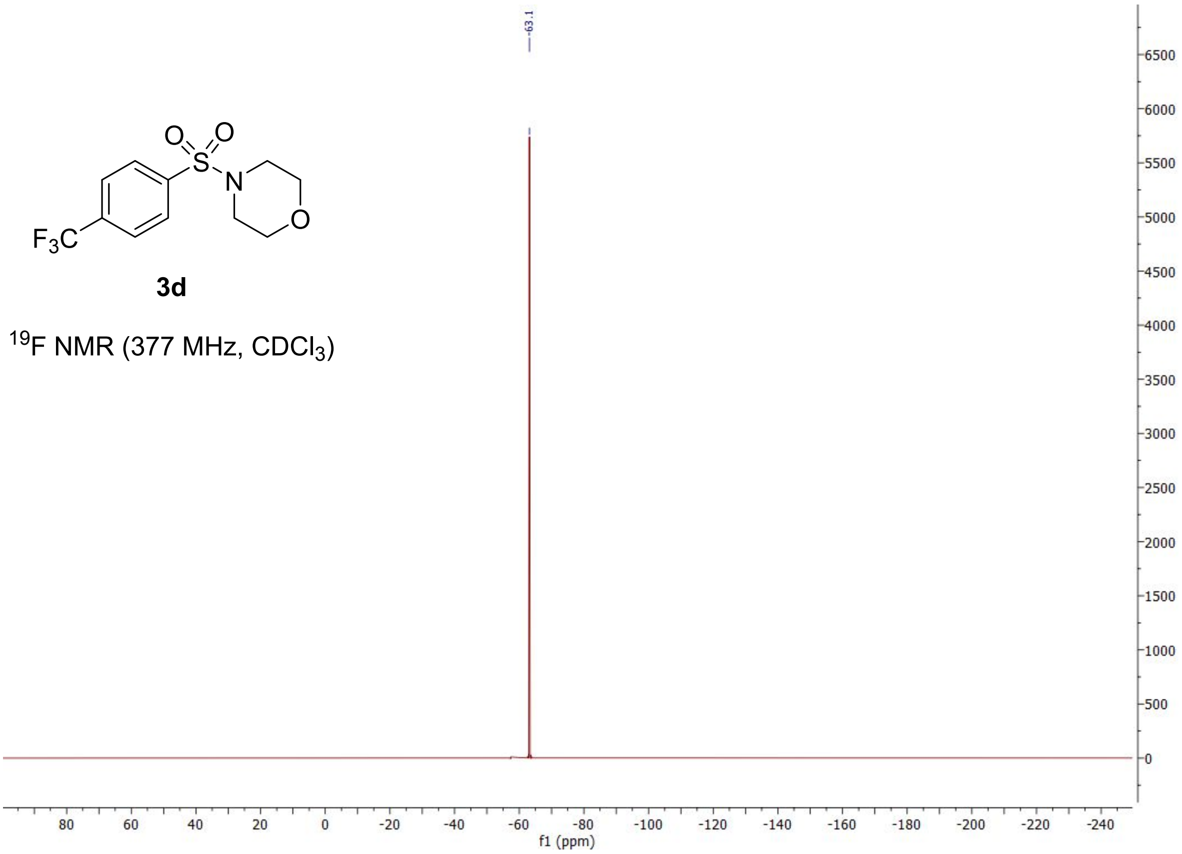

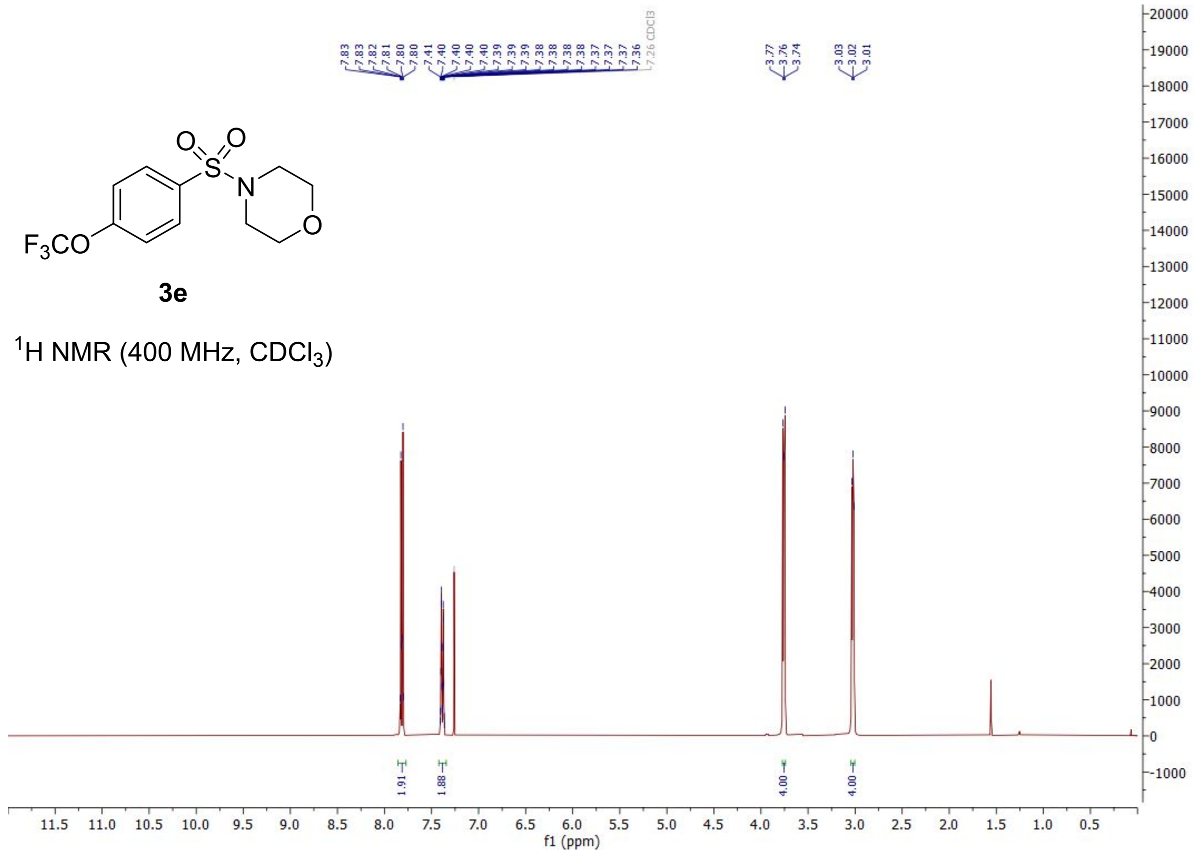
F$_3$CO
3e
^{1}H NMR (400 MHz, CDCl$_3$)
7.83 7.83 7.82 7.81 7.80 7.80 7.41 7.40 7.40 7.40 7.39 7.39 7.39 7.38 7.38 7.38 7.38 7.37 7.37 7.37 7.36
7.26 CDCl3
3.77 3.76 3.74
3.03 3.02 3.01
1.91 1.88 4.00 4.00
f1 (ppm)

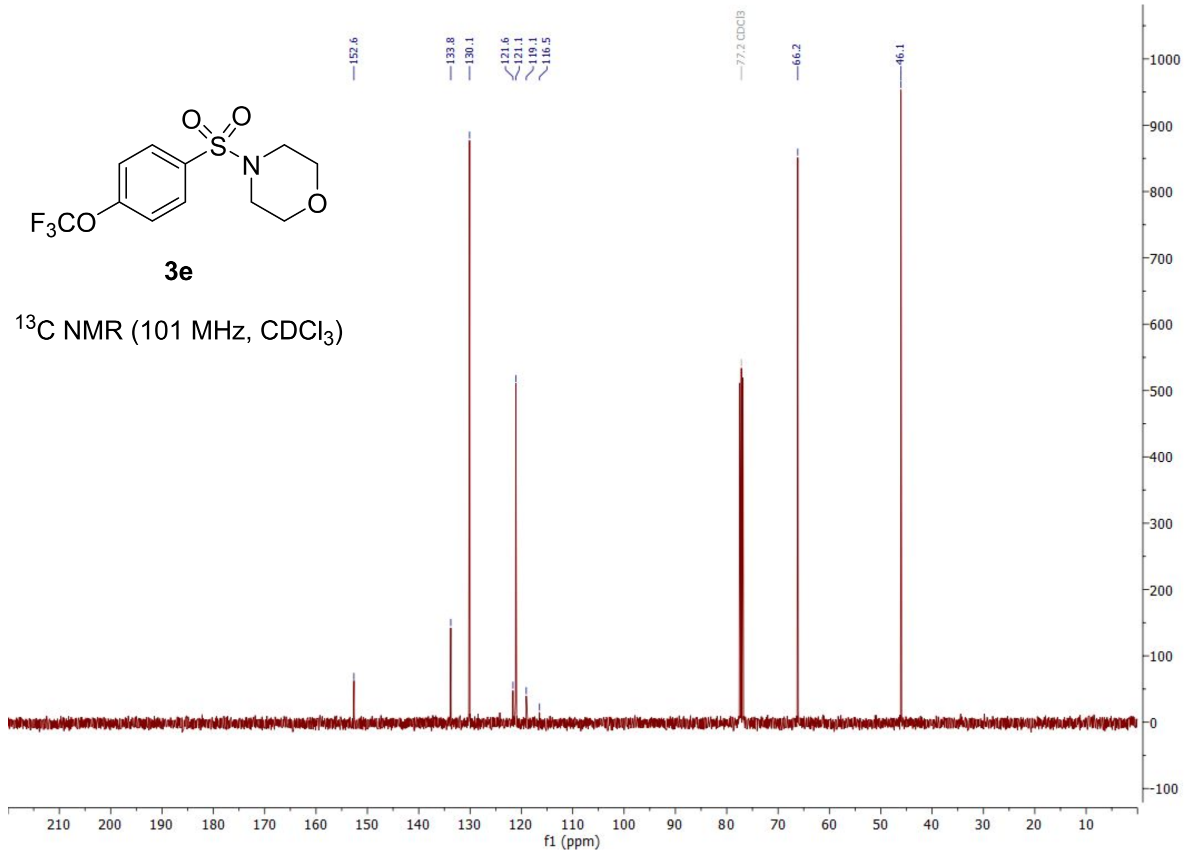
F$_3$CO
3e
^{13}C NMR (101 MHz, CDCl$_3$)
152.6
133.8 130.1
121.6 121.1 119.1 116.5
77.2 CDCl3
66.2
46.1
f1 (ppm)

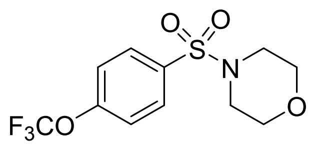

3e

^{19}F NMR (377 MHz, $CDCl_3$)

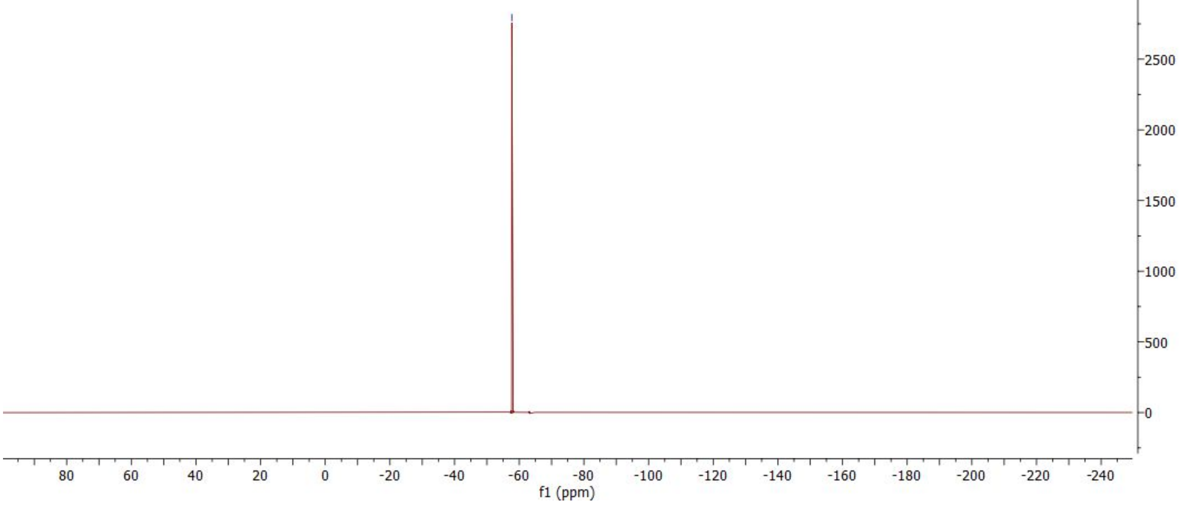

3f

^{1}H NMR (400 MHz, $CDCl_3$)

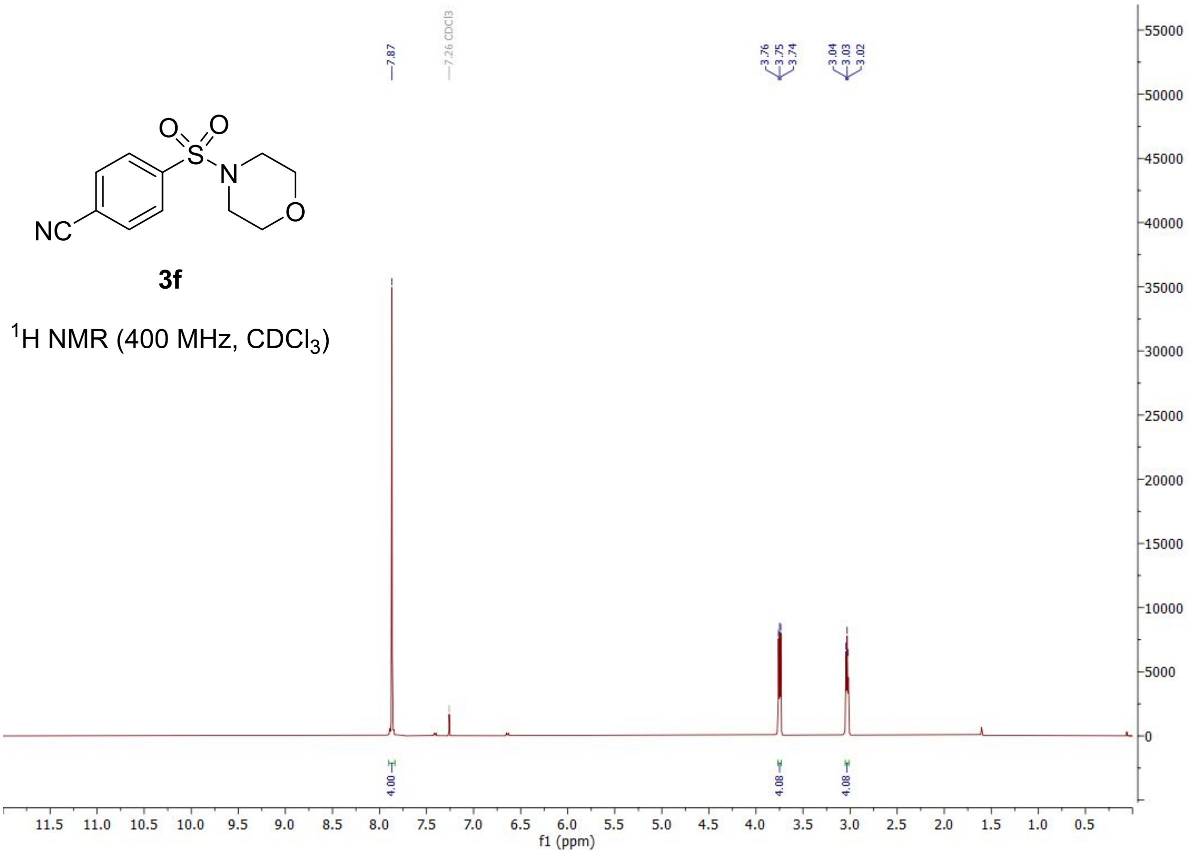

3f

^{13}C NMR (101 MHz, $CDCl_3$)

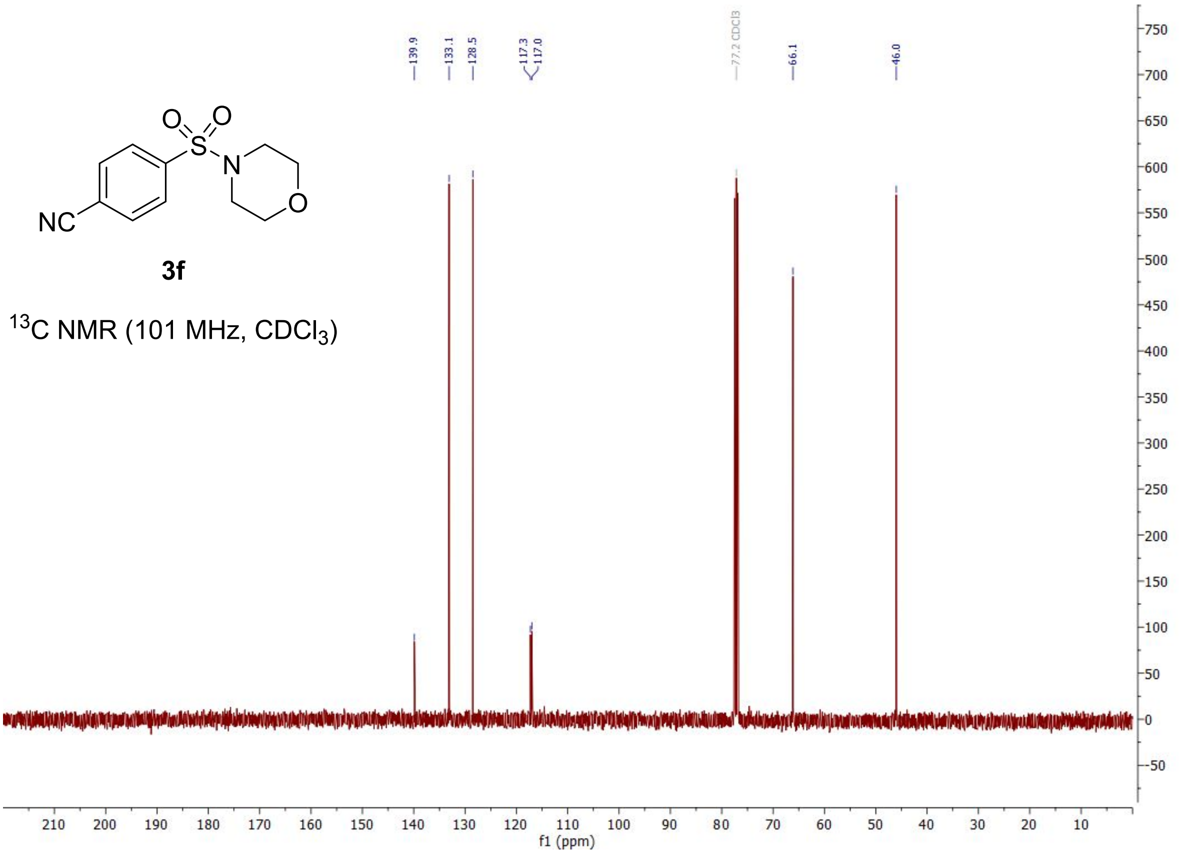

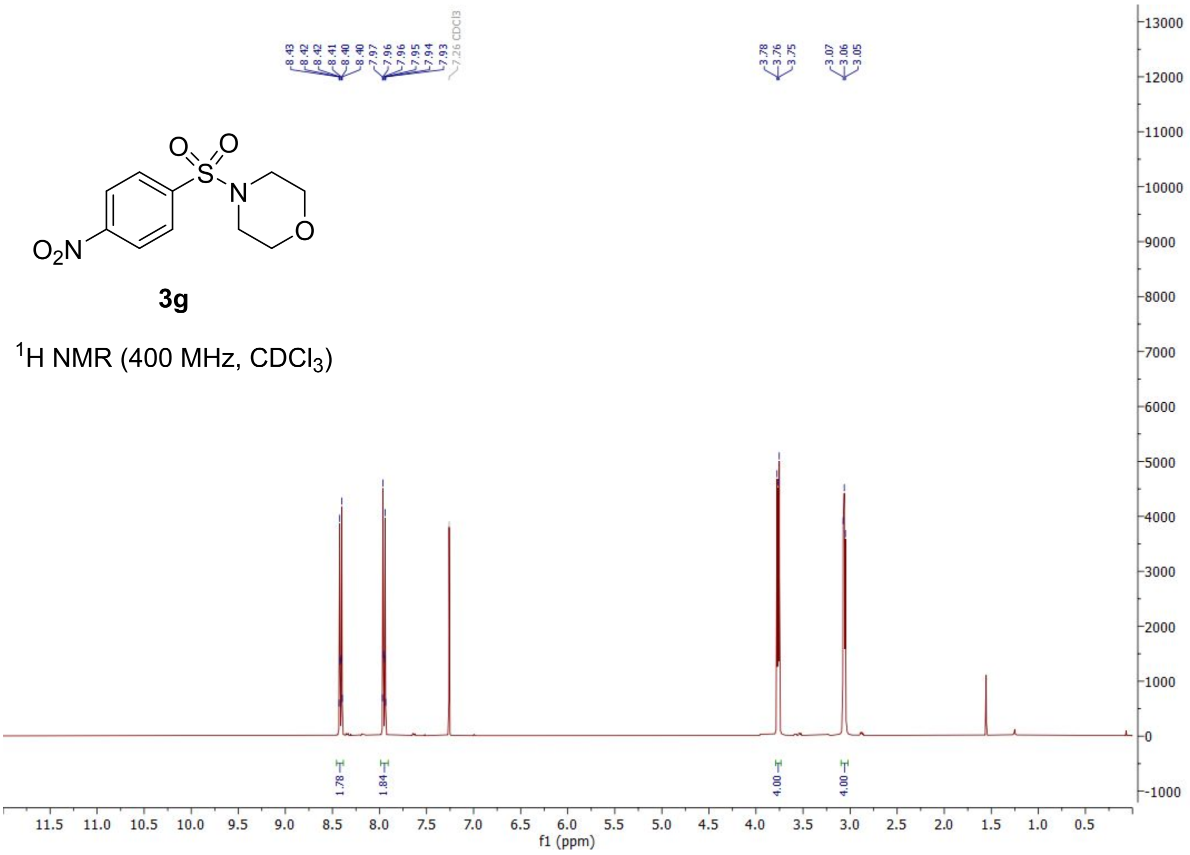

3g

^{1}H NMR (400 MHz, $CDCl_3$)

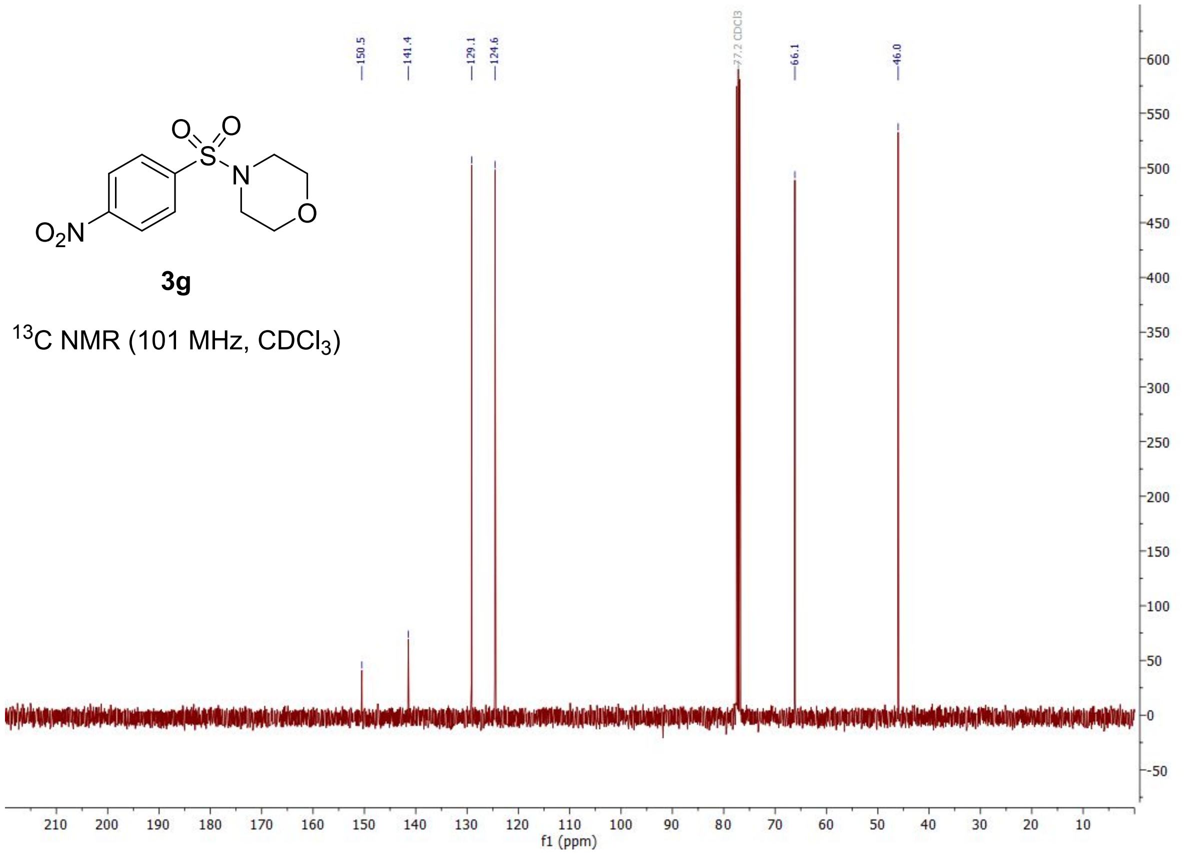

3g

^{13}C NMR (101 MHz, $CDCl_3$)

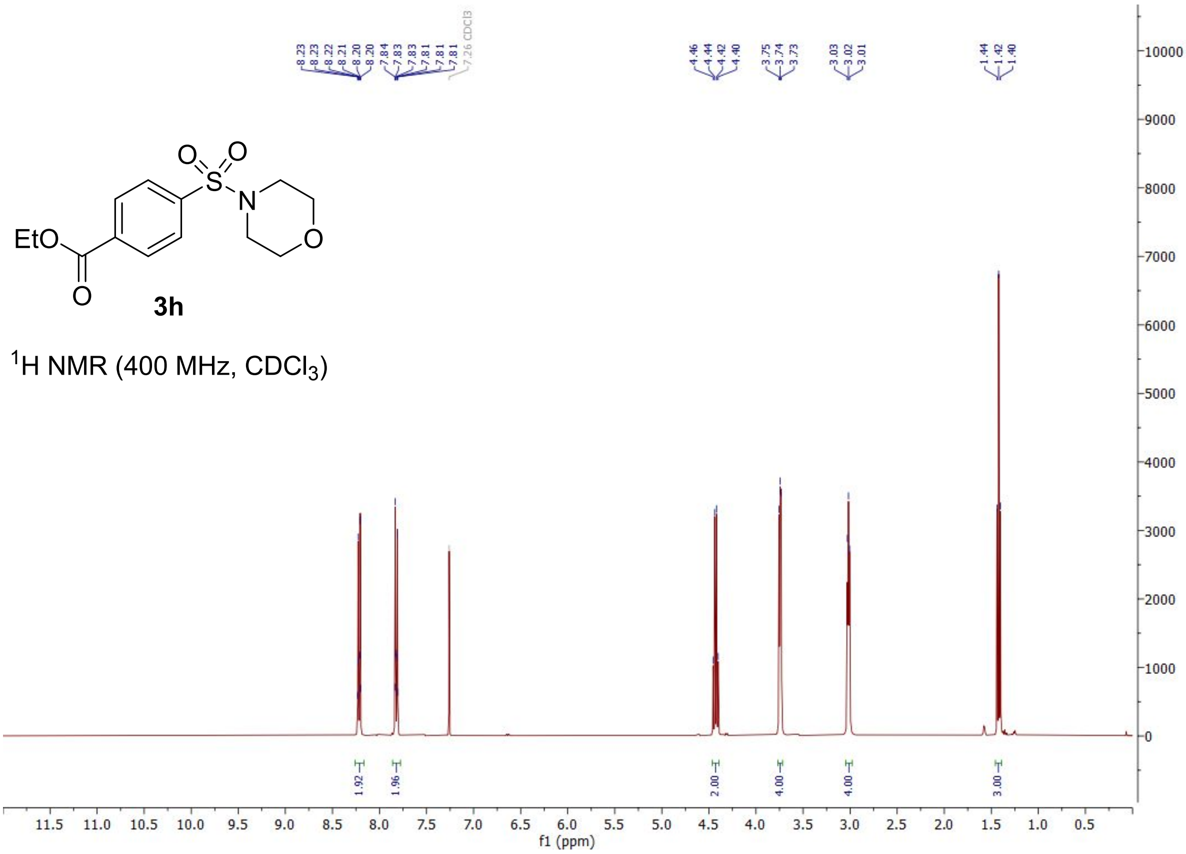

EtO
3h

^{1}H NMR (400 MHz, $CDCl_3$)

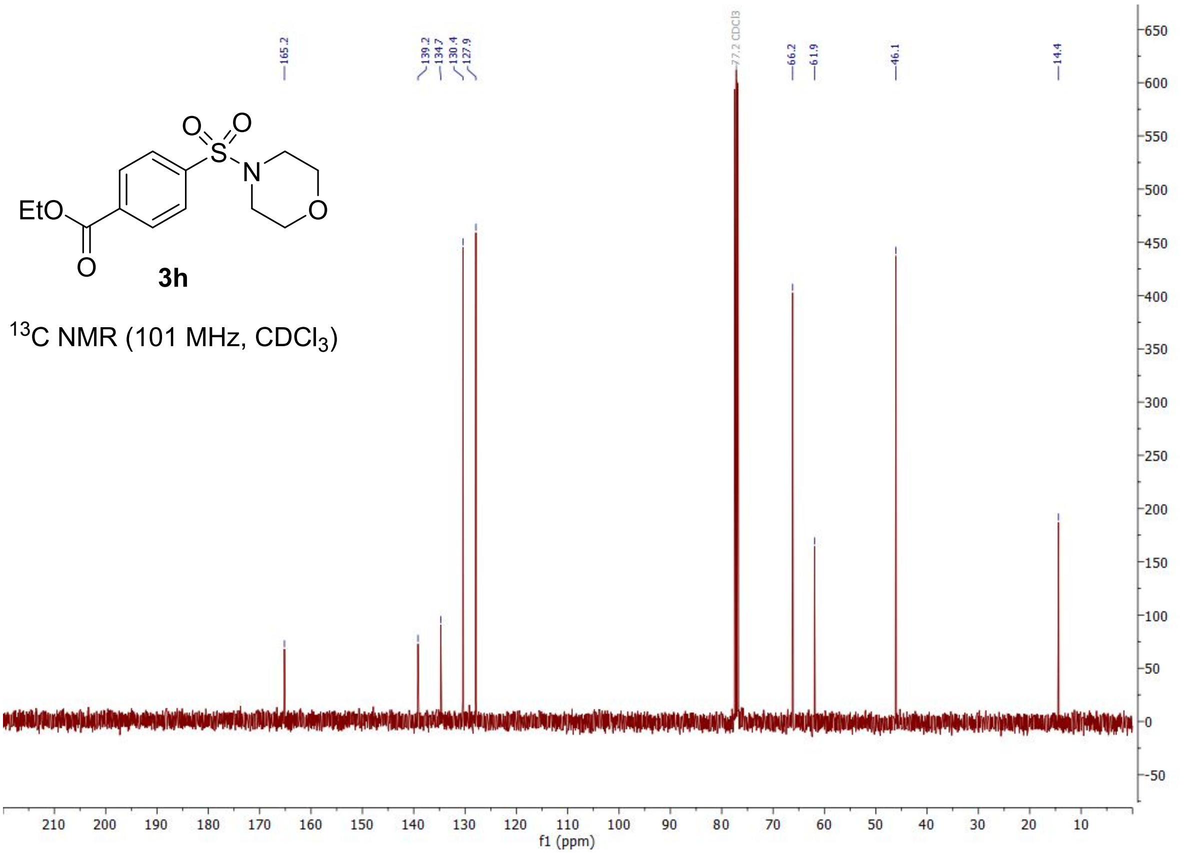

EtO
3h

^{13}C NMR (101 MHz, $CDCl_3$)

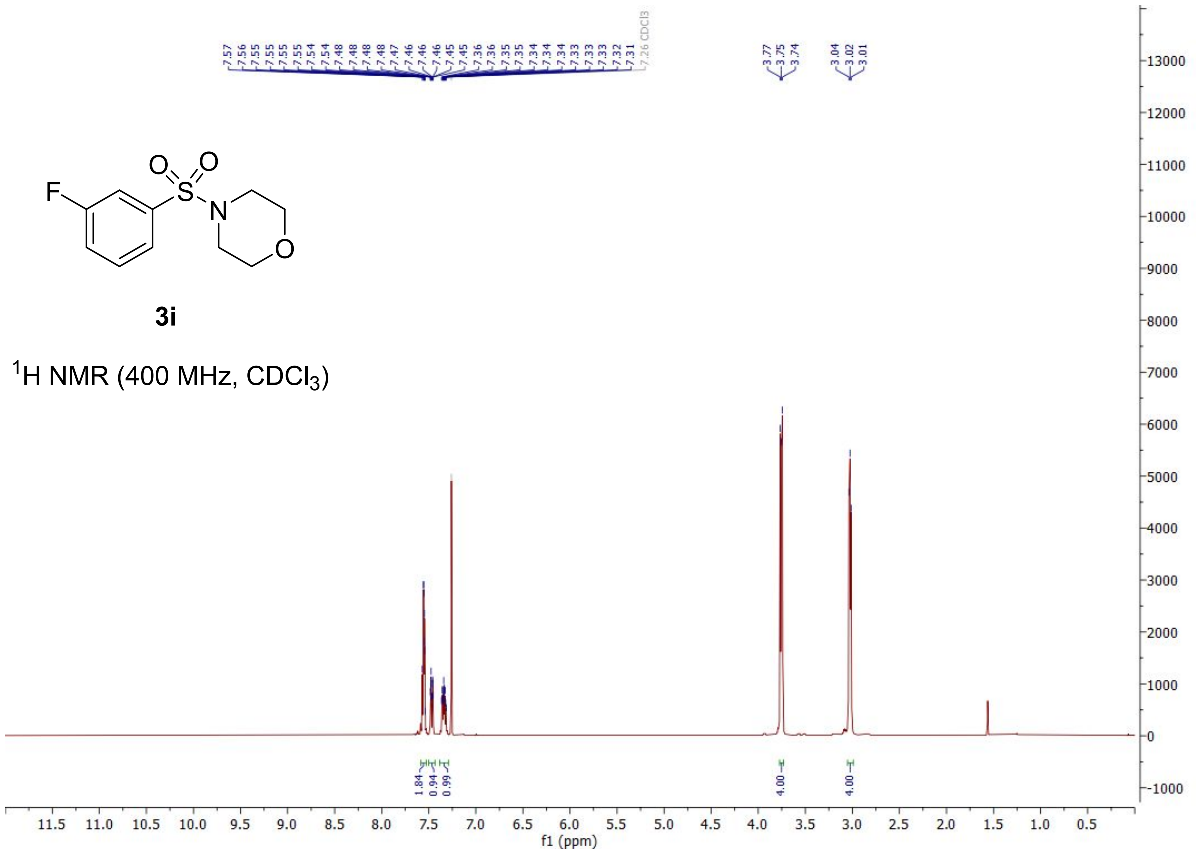
7.57 7.56 7.55 7.55 7.55 7.55 7.54 7.54 7.48 7.48 7.48 7.48 7.47 7.46 7.46 7.46 7.45 7.45 7.36 7.36 7.36 7.35 7.35 7.34 7.34 7.34 7.33 7.33 7.33 7.32 7.31 7.26 CDCl3
3.77 3.75 3.74
3.04 3.02 3.01
F
O
S
O
N
O
3i
^{1}H NMR (400 MHz, $CDCl_3$)
1.84 0.94 0.99
4.00
4.00
f1 (ppm)

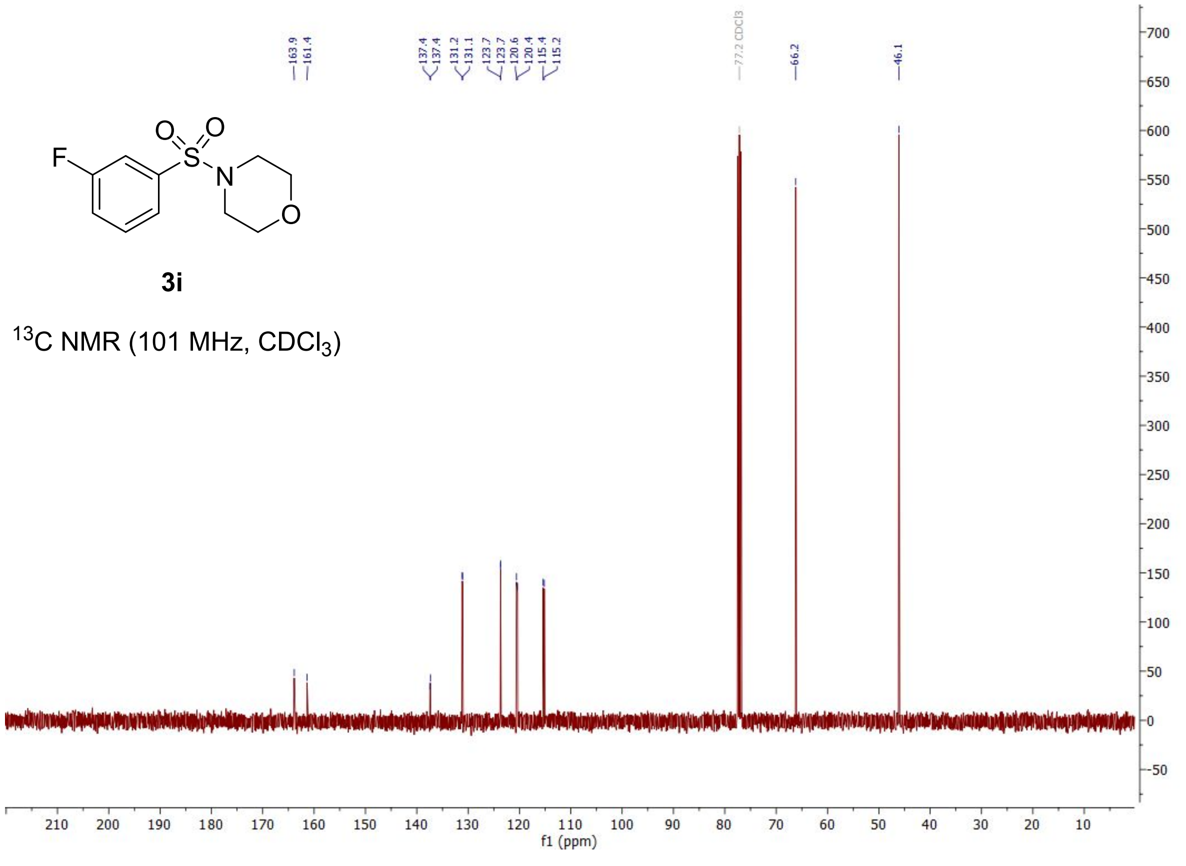
163.9 161.4
137.4 137.4 131.2 131.1 123.7 123.7 120.6 120.4 115.4 115.2
77.2 CDCl3
66.2
46.1
F
O
S
O
N
O
3i
^{13}C NMR (101 MHz, $CDCl_3$)
f1 (ppm)

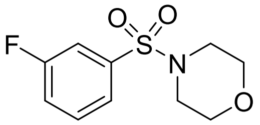

3i

^{19}F NMR (377 MHz, $CDCl_3$)

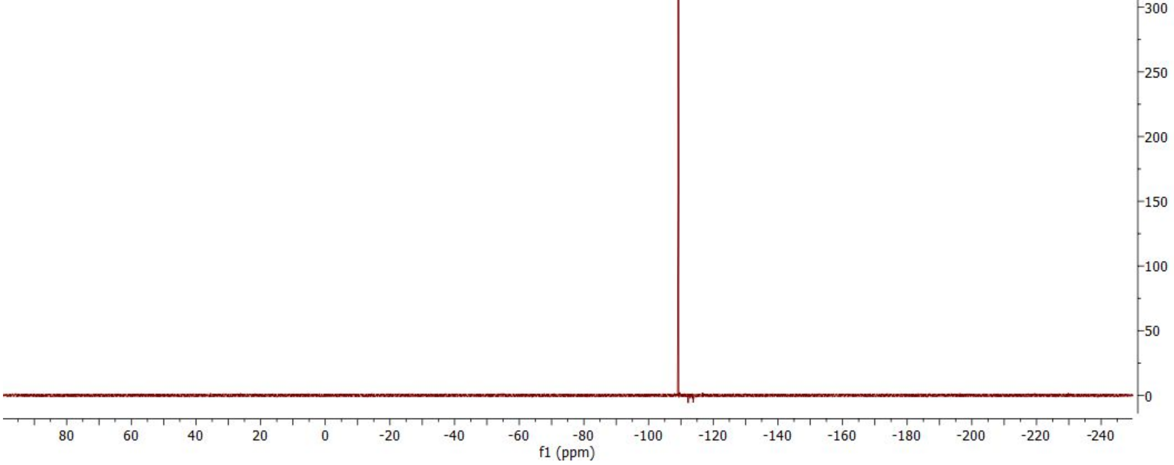

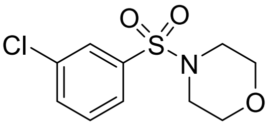

3j

^{1}H NMR (400 MHz, $CDCl_3$)

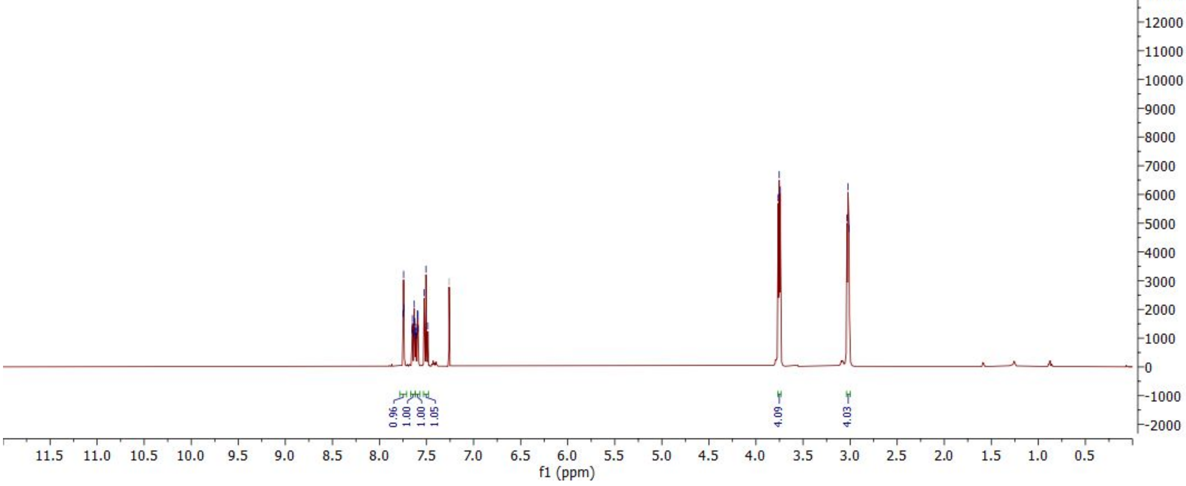

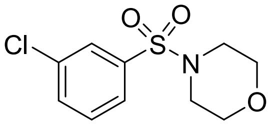

3j

^{13}C NMR (101 MHz, $CDCl_3$)

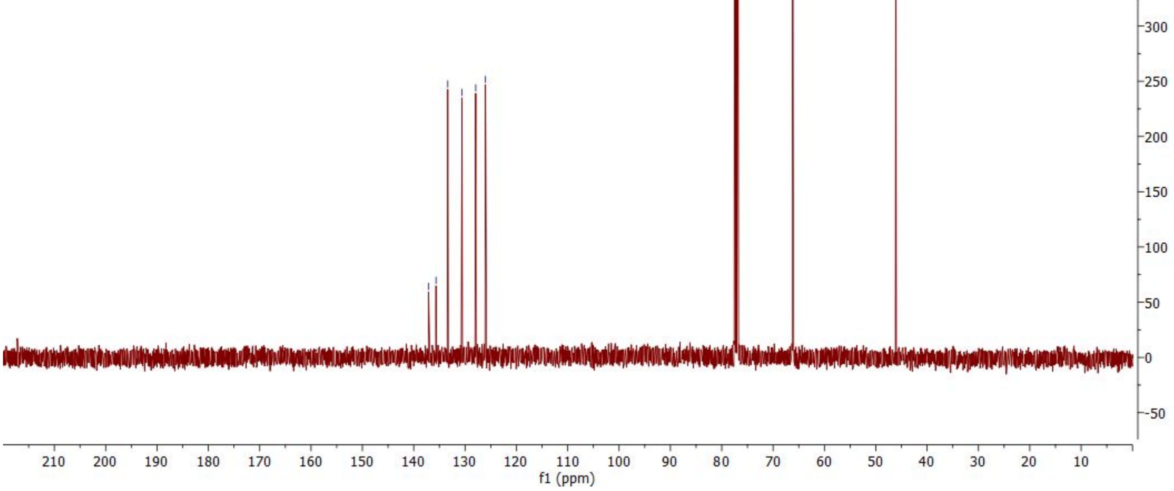

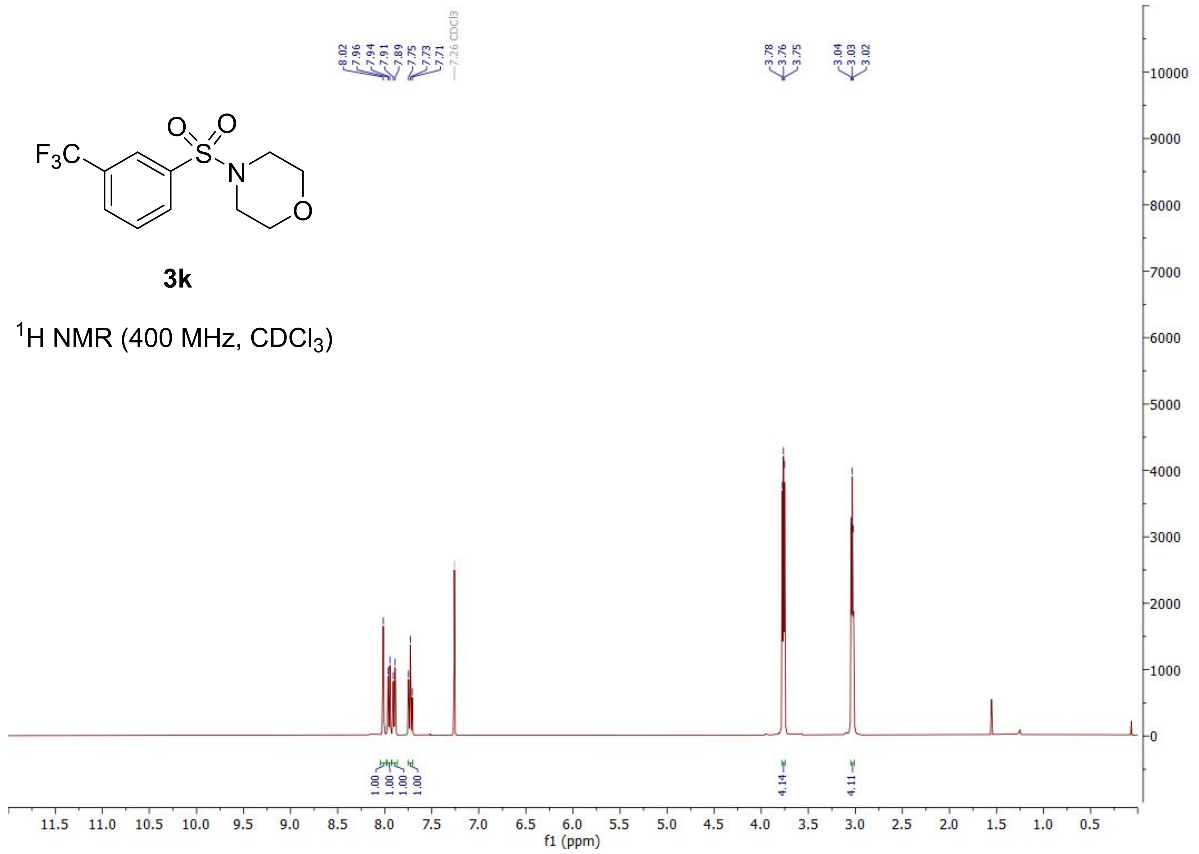

3k

^{1}H NMR (400 MHz, $CDCl_3$)

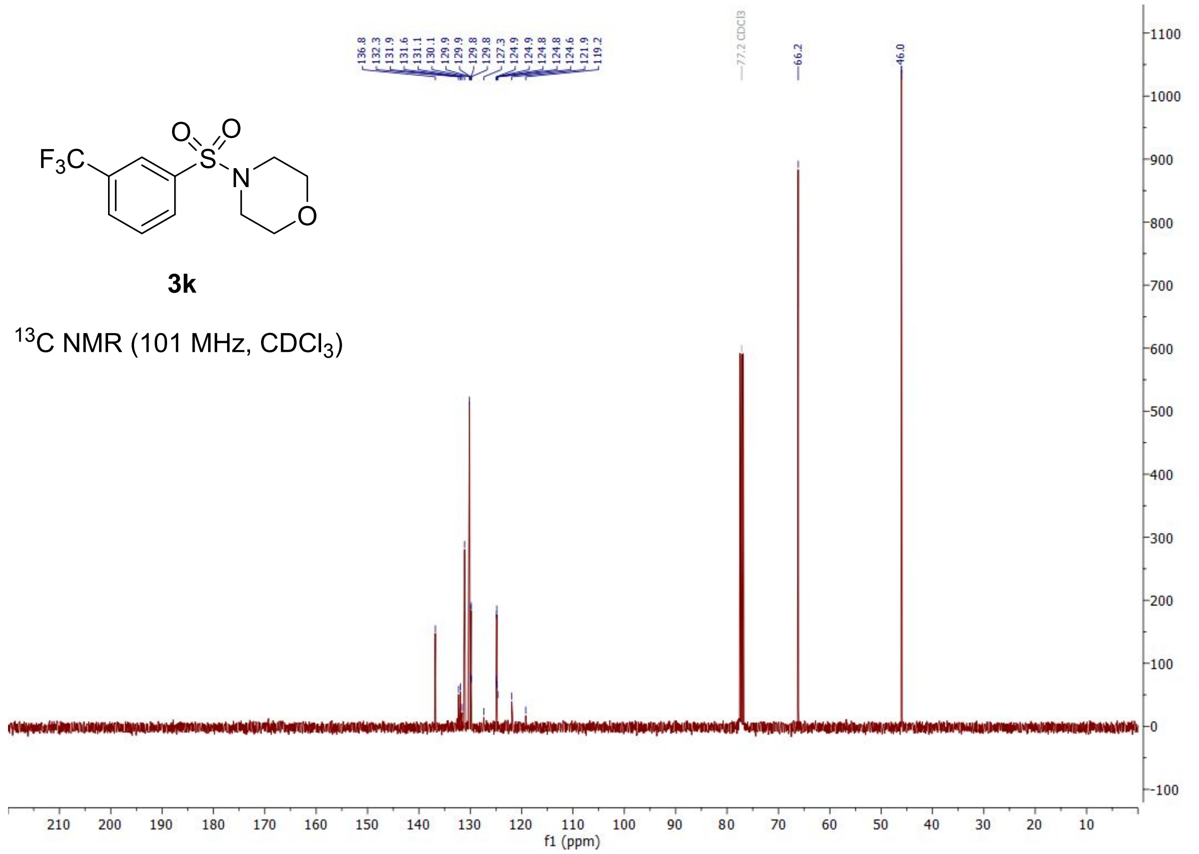

3k

^{13}C NMR (101 MHz, $CDCl_3$)

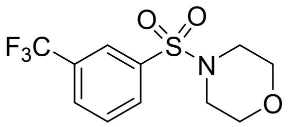

3k

^{19}F NMR (377 MHz, CDCl$_3$)

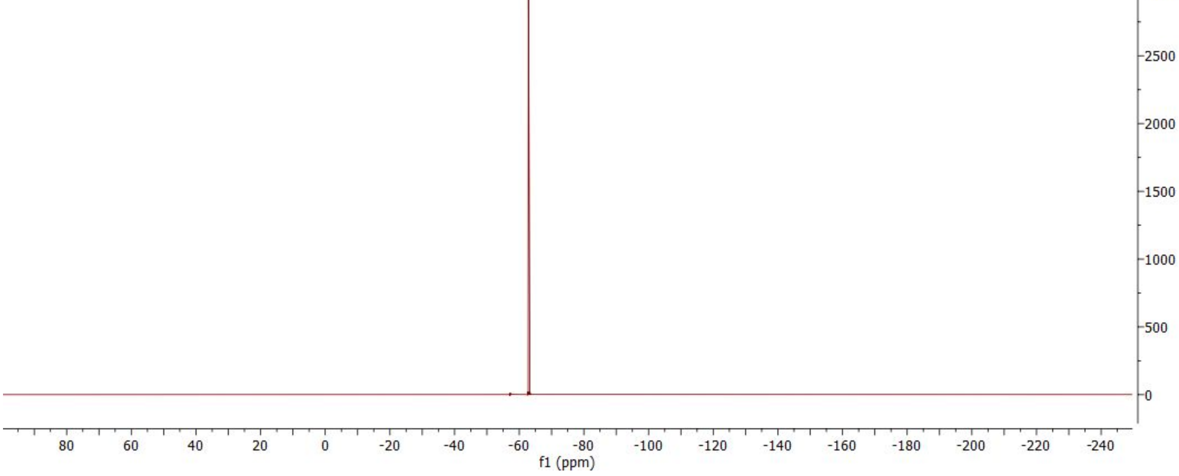

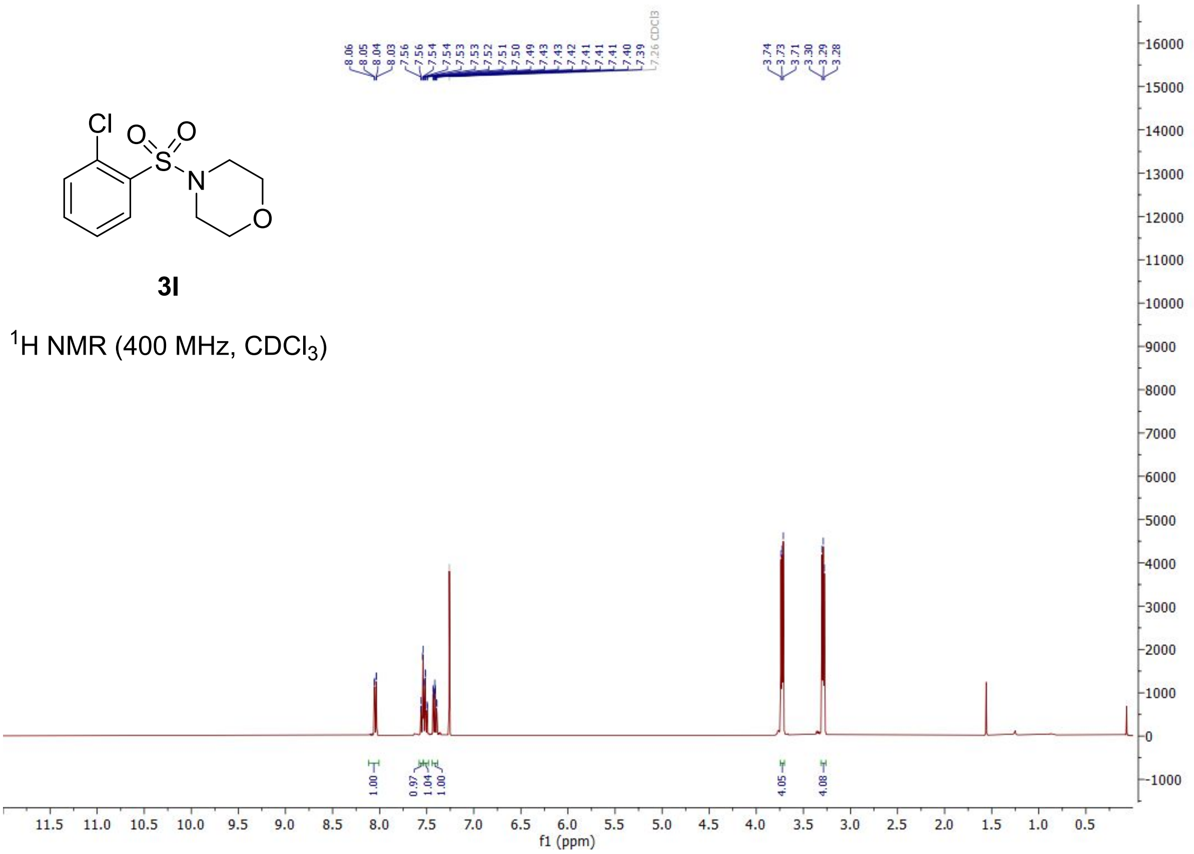

3l

^{1}H NMR (400 MHz, $CDCl_3$)

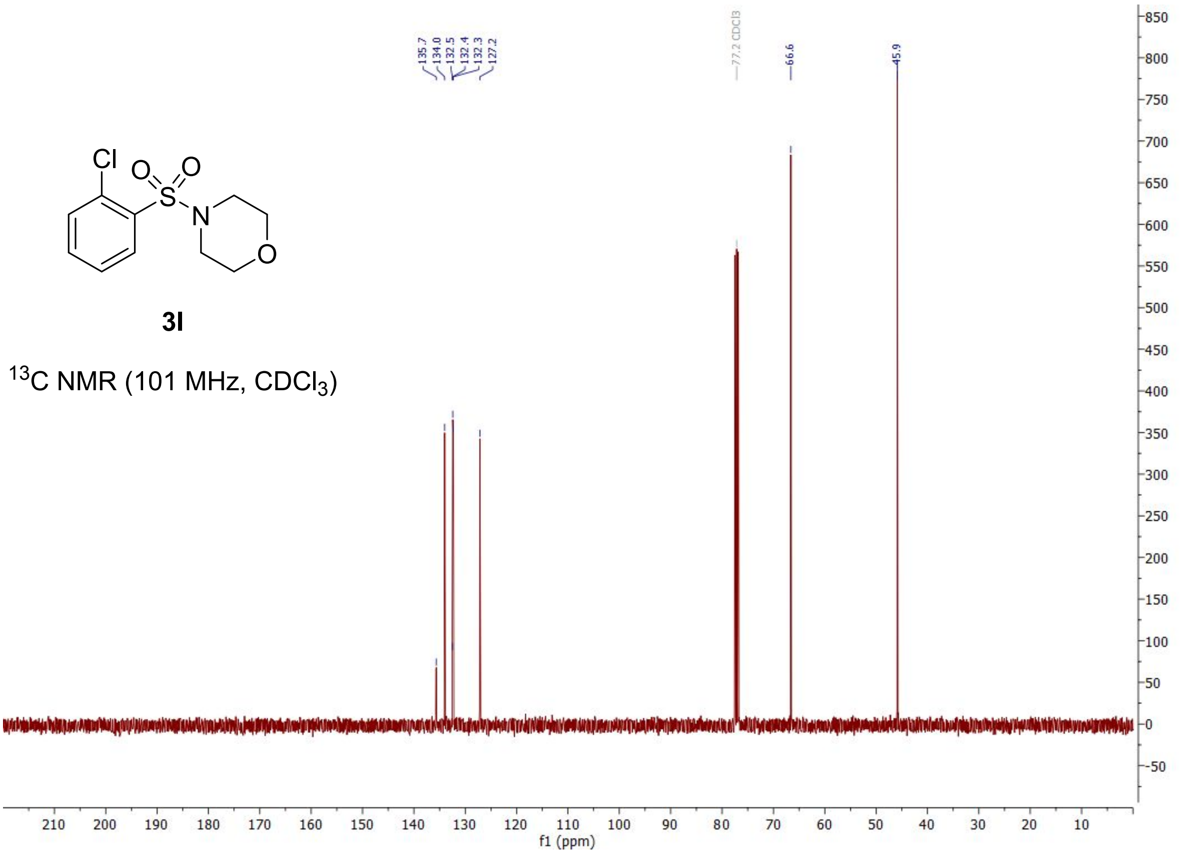

3l

^{13}C NMR (101 MHz, $CDCl_3$)

3m

^1H NMR (400 MHz, $CDCl_3$)

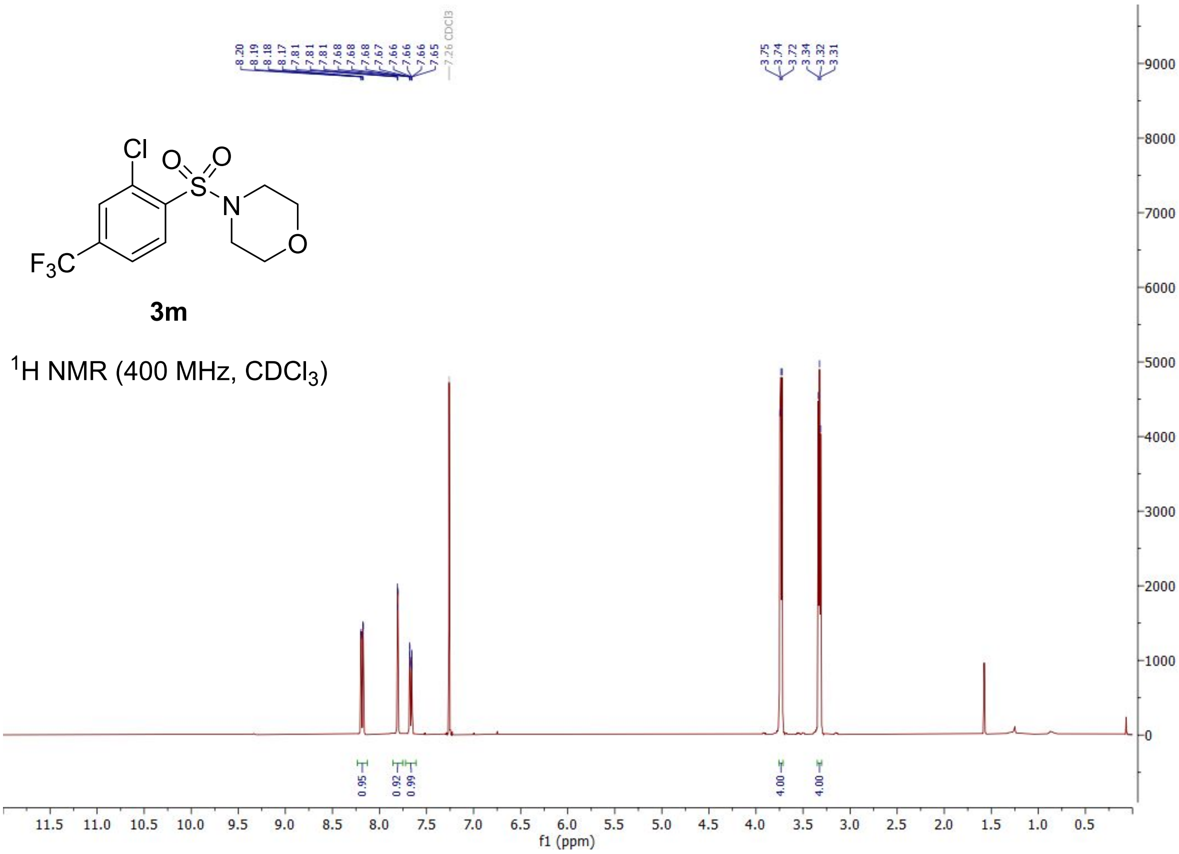

3m

^{13}C NMR (101 MHz, $CDCl_3$)

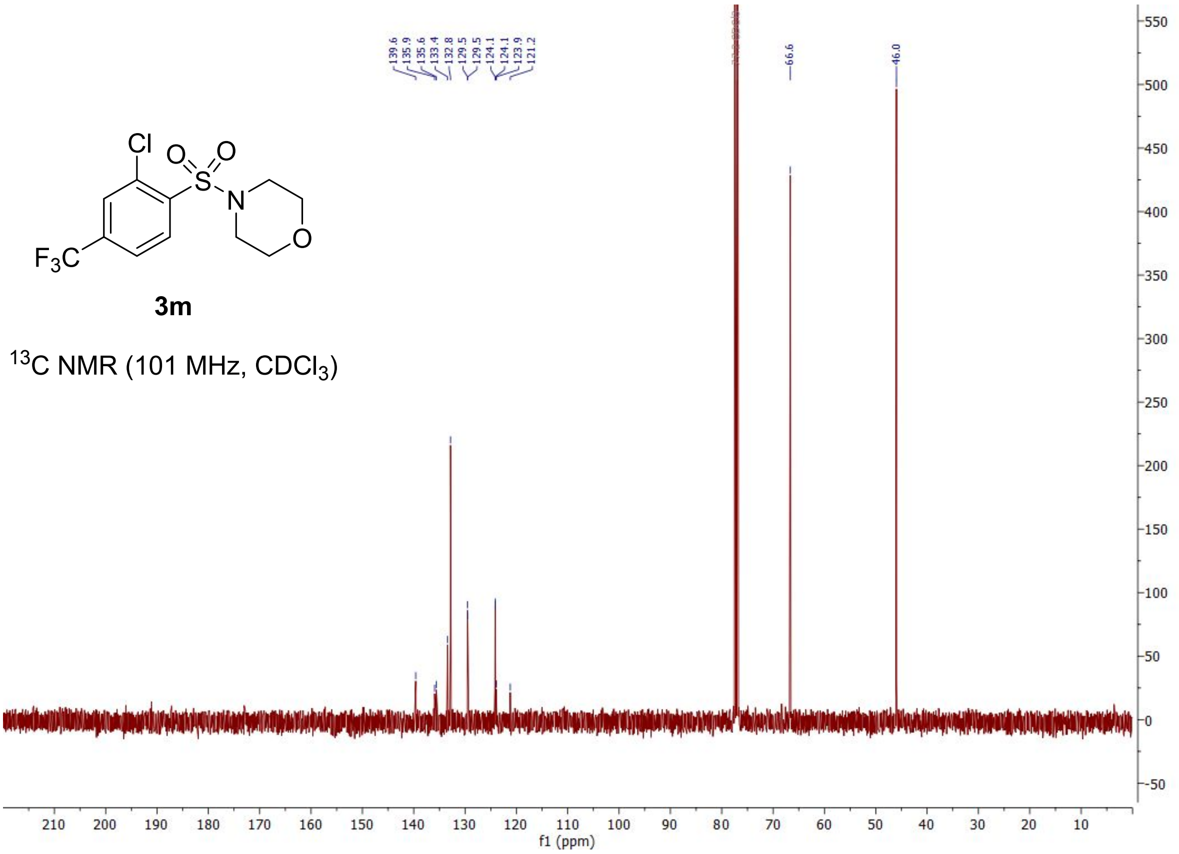

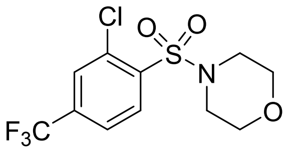

3m

^{19}F NMR (377 MHz, $CDCl_3$)

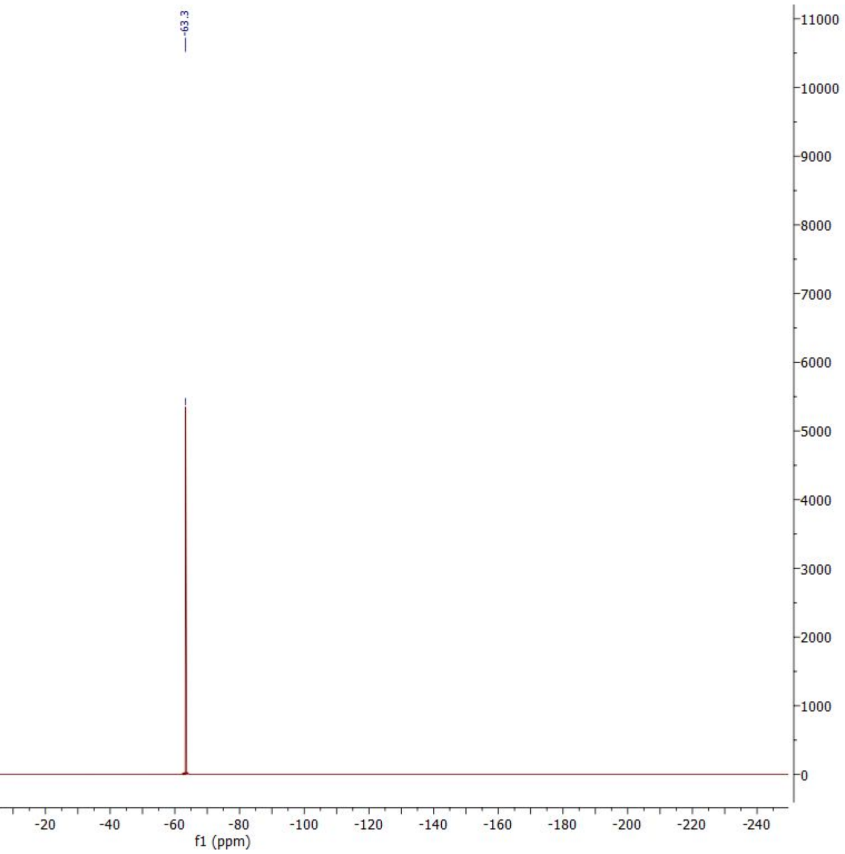

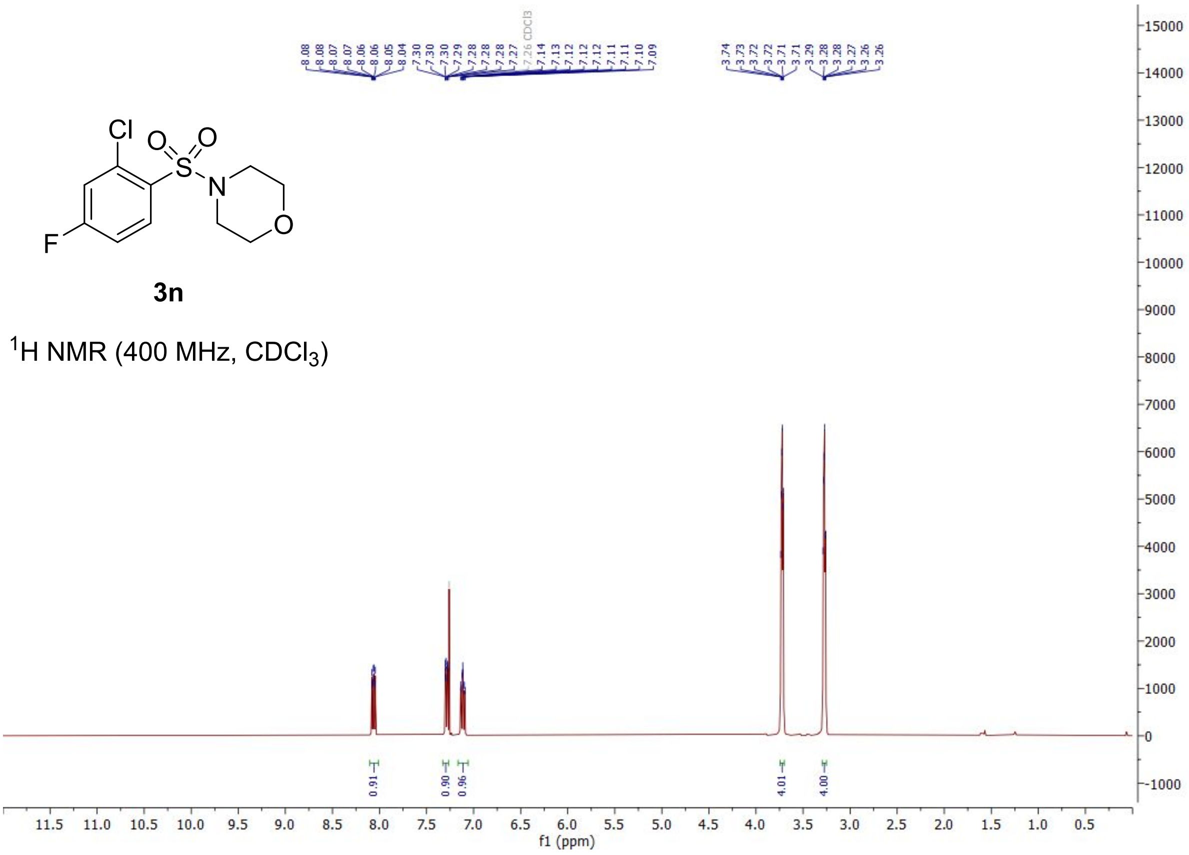

3n

^{1}H NMR (400 MHz, $CDCl_3$)

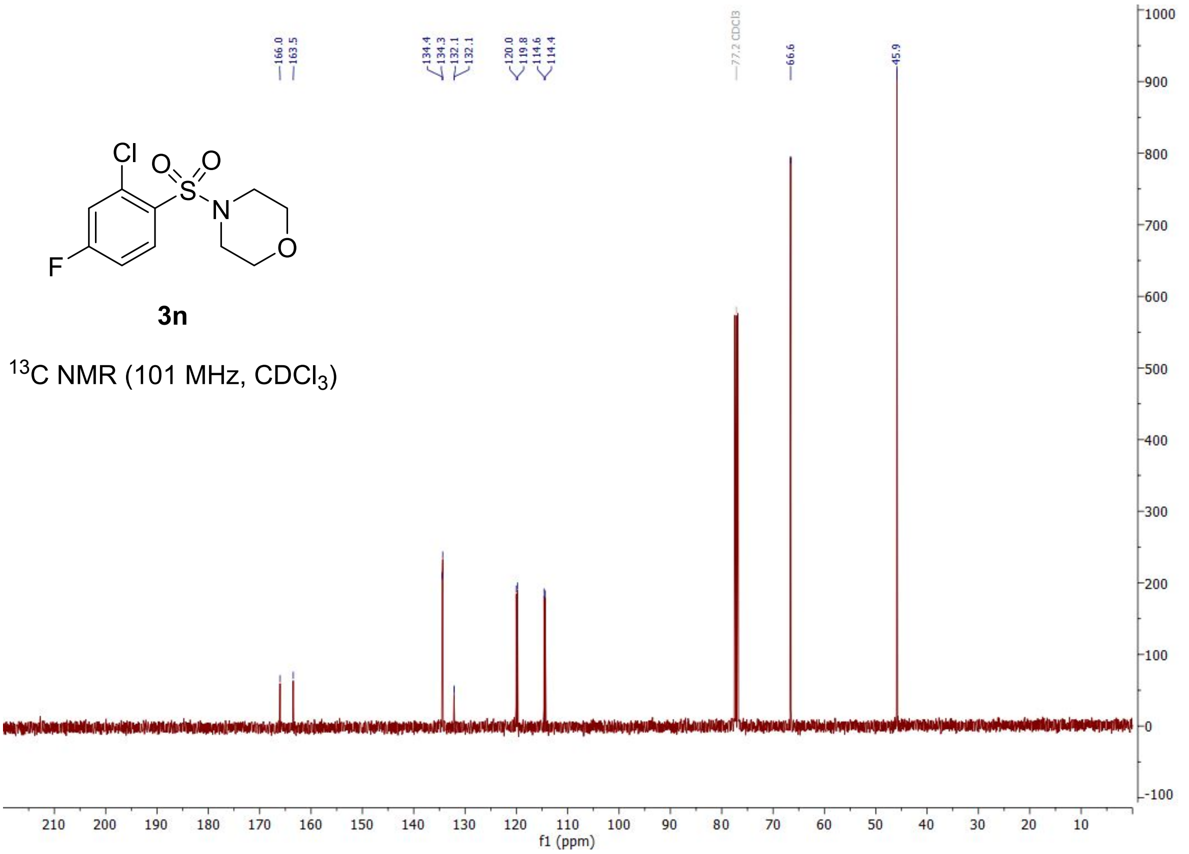

3n

^{13}C NMR (101 MHz, $CDCl_3$)

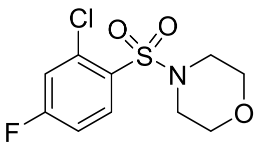

3n

^{19}F NMR (377 MHz, $CDCl_3$)

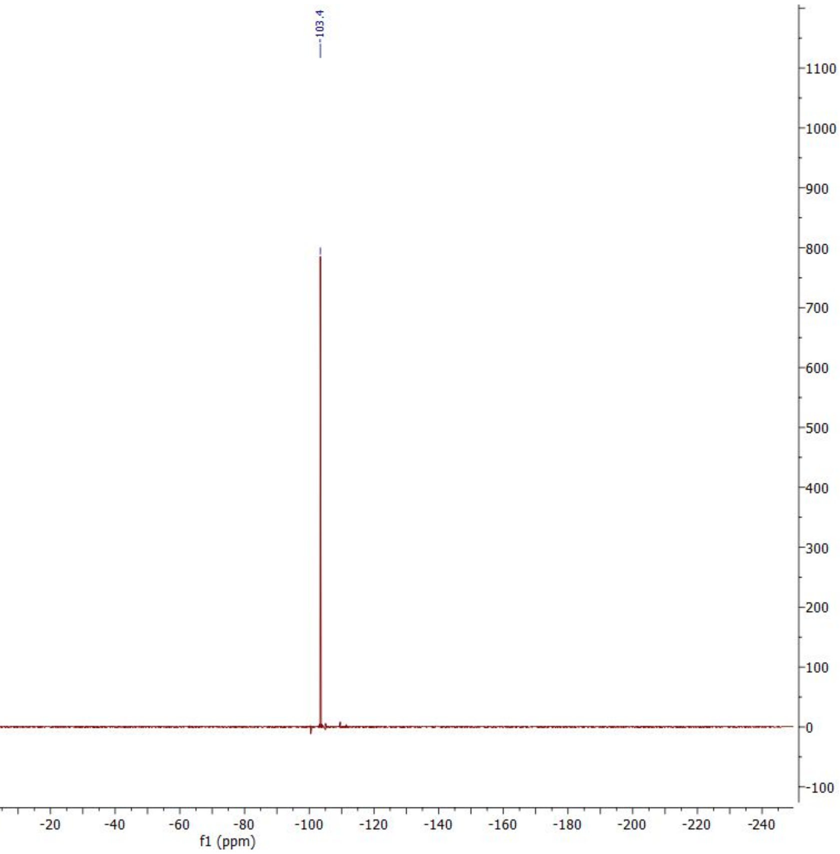

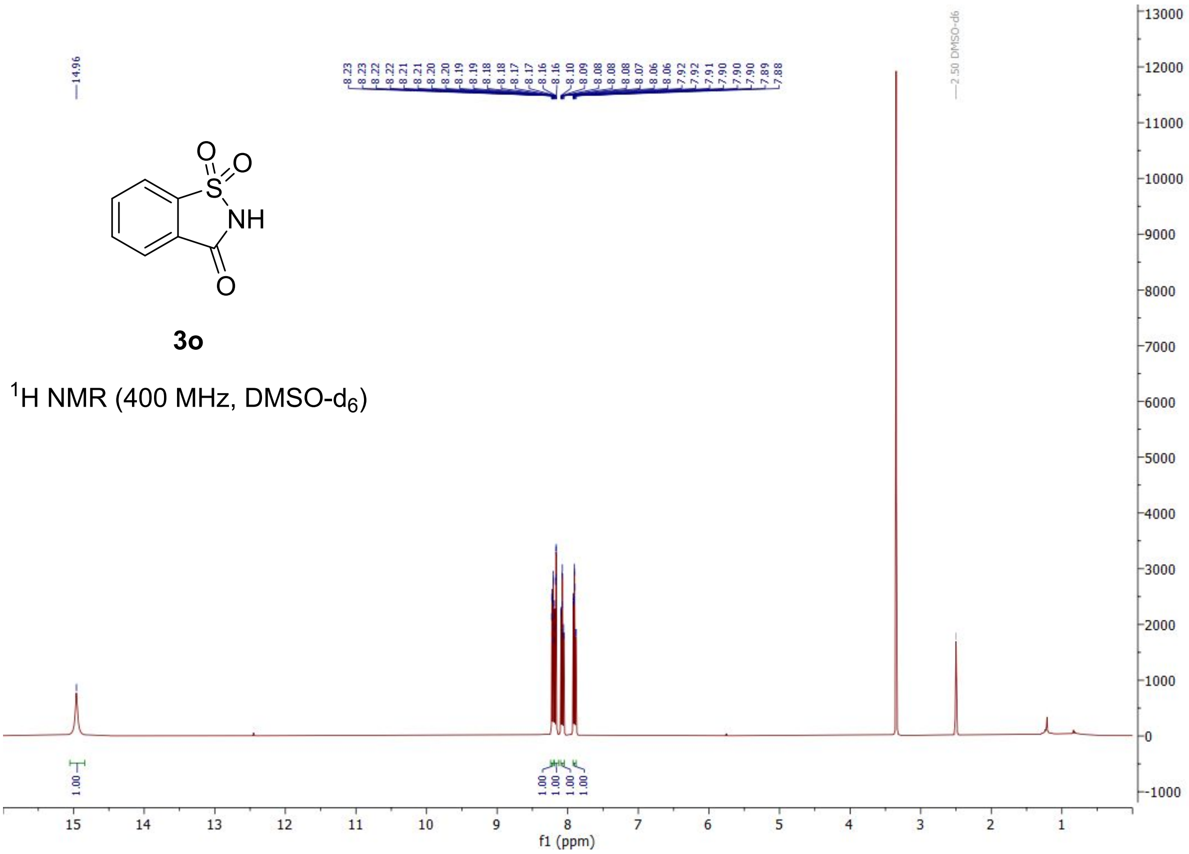

3o

^{1}H NMR (400 MHz, DMSO-d_6)

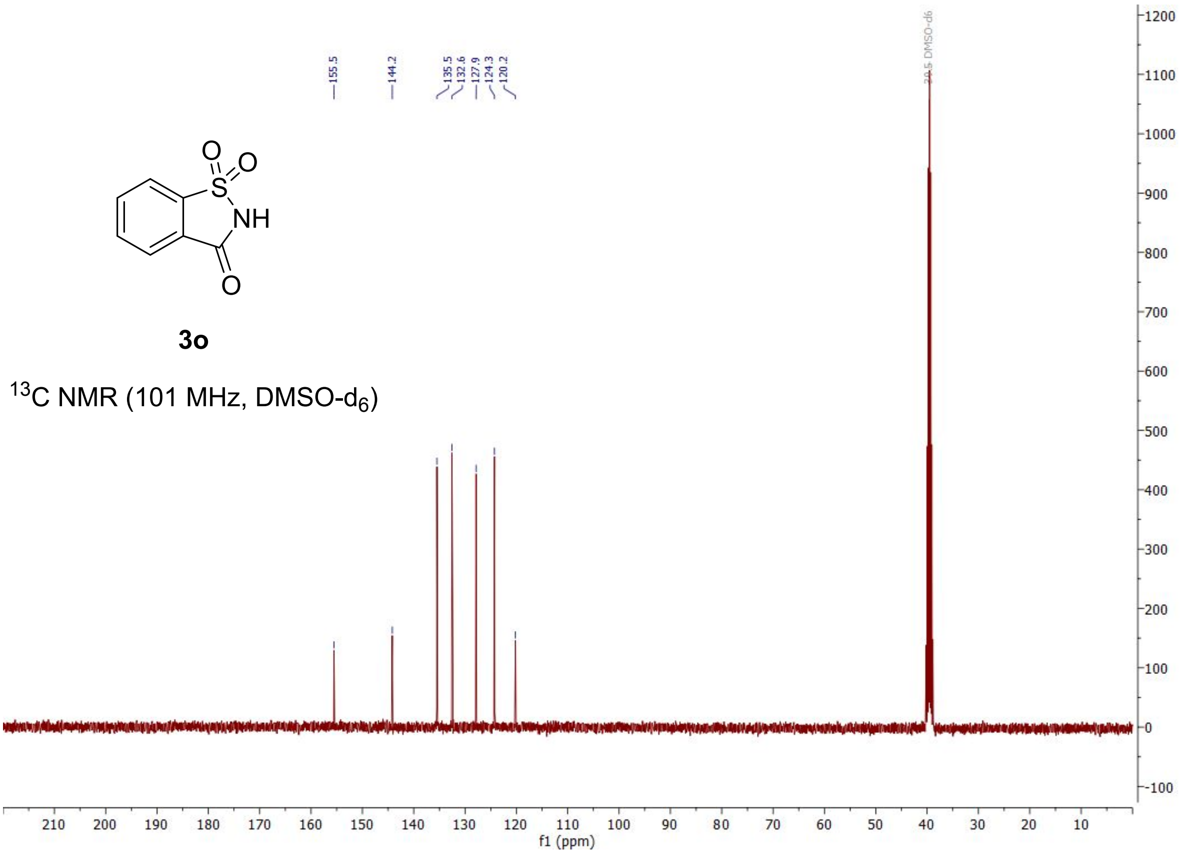

3o

^{13}C NMR (101 MHz, DMSO-d_6)

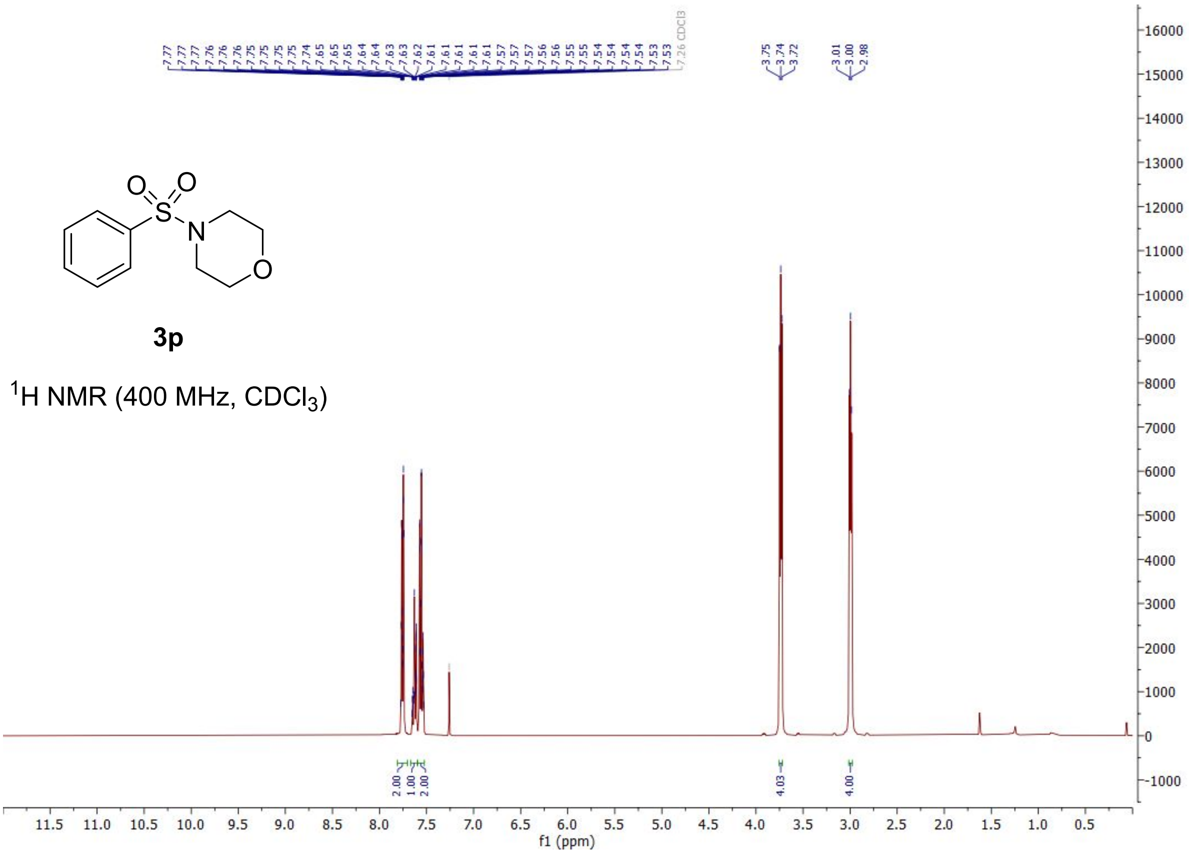

3p

^1H NMR (400 MHz, $CDCl_3$)

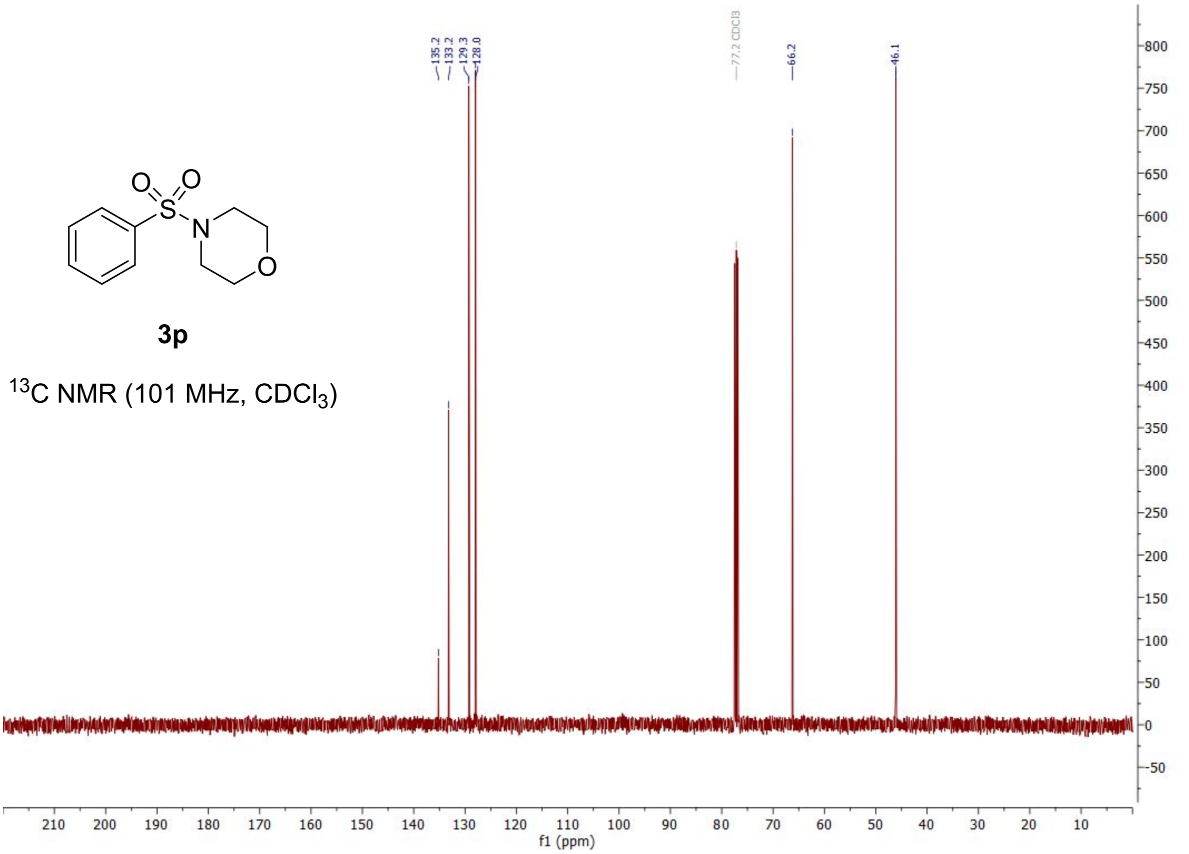

3p

^{13}C NMR (101 MHz, $CDCl_3$)

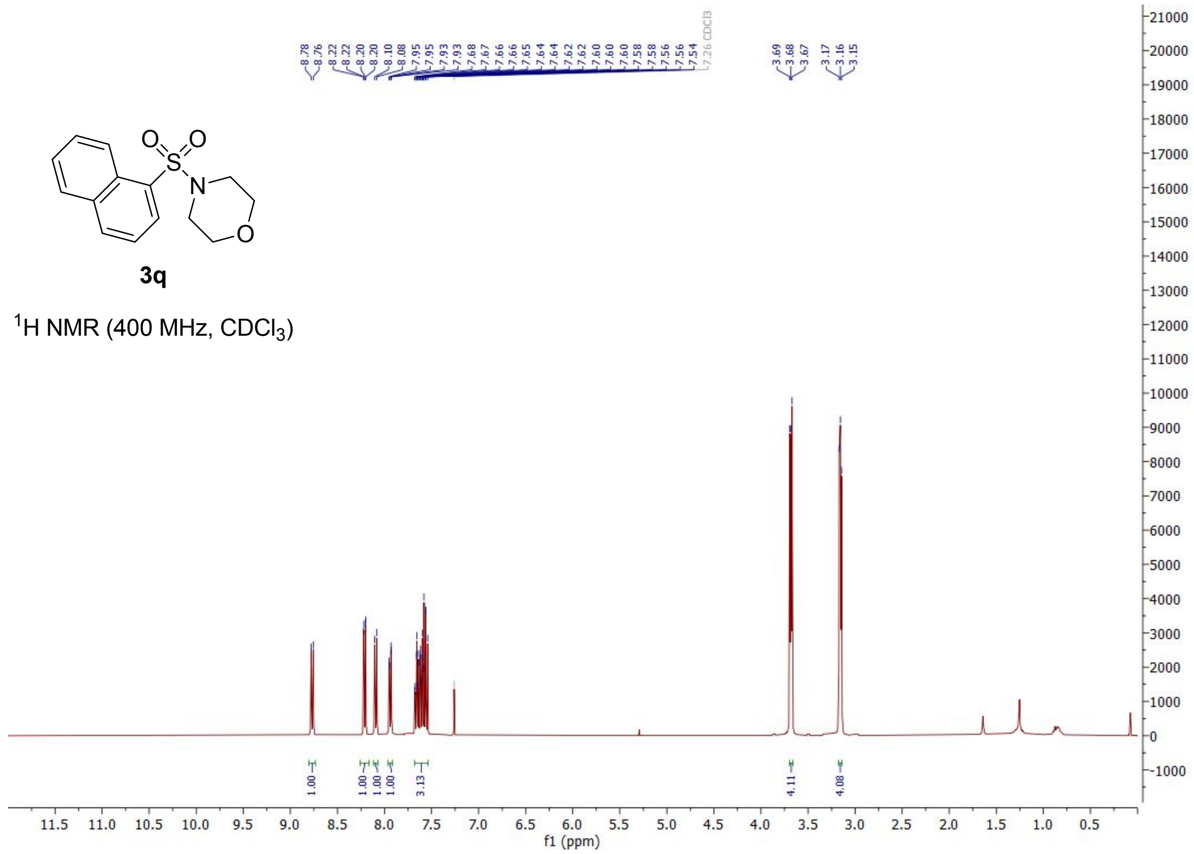
3q
¹H NMR (400 MHz, CDCl₃)
8.78
8.76
8.22
8.22
8.20
8.20
8.10
8.08
7.95
7.95
7.93
7.93
7.68
7.67
7.66
7.65
7.64
7.64
7.62
7.62
7.60
7.60
7.60
7.58
7.58
7.56
7.56
7.54
7.26 CDCl3
3.69
3.68
3.67
3.17
3.16
3.15
1.00
1.00
1.00
1.00
3.13
4.11
4.08
f1 (ppm)

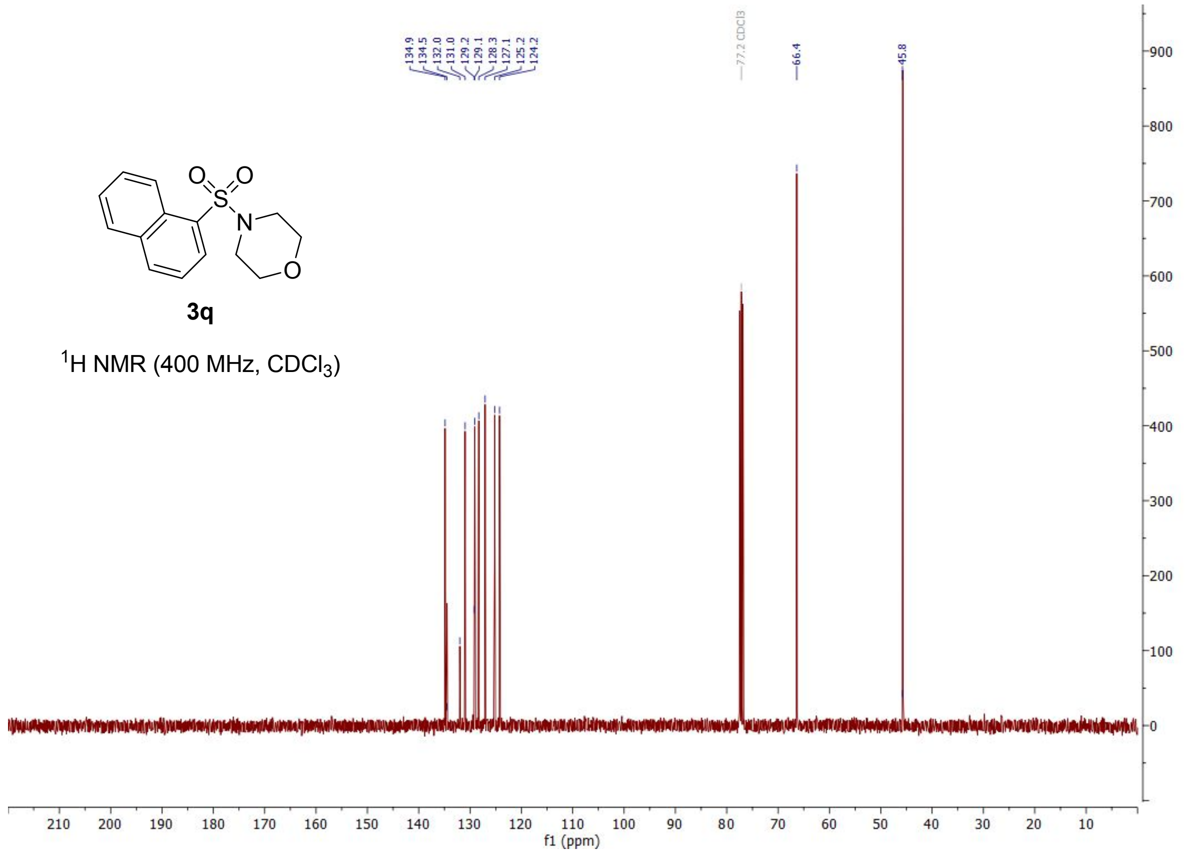
3q
¹H NMR (400 MHz, CDCl₃)
134.9
134.5
132.0
131.0
129.2
129.1
128.3
127.1
125.2
124.2
77.2 CDCl3
66.4
45.8
f1 (ppm)

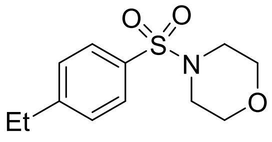

3r

^{1}H NMR (400 MHz, $CDCl_3$)

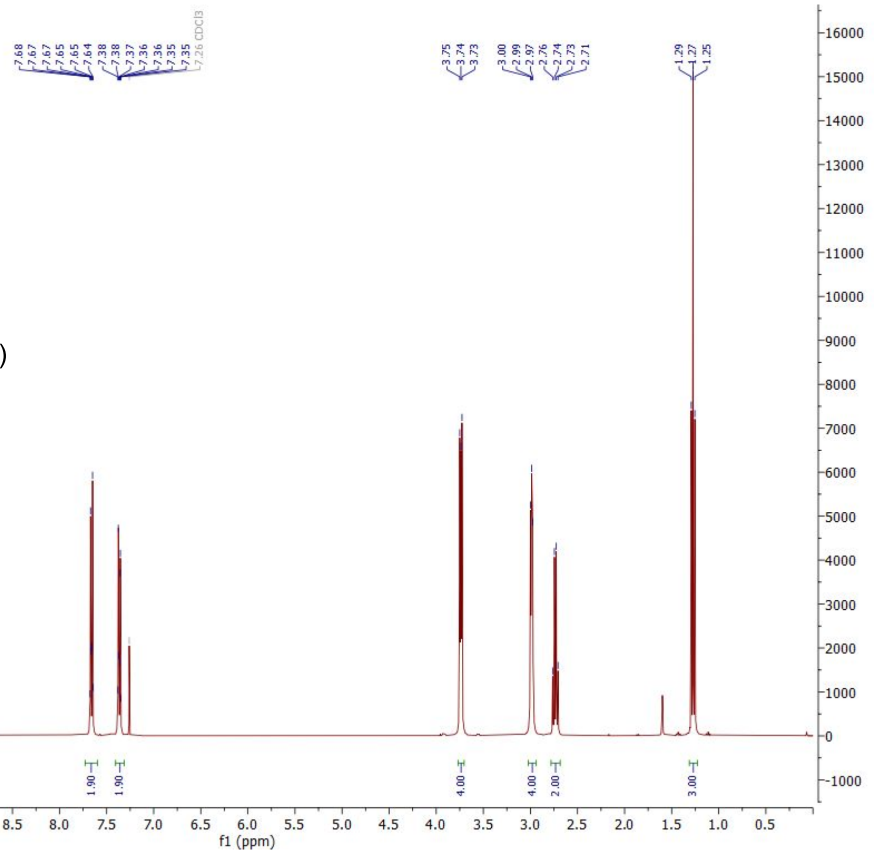

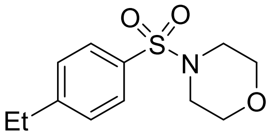

3r

^{13}C NMR (101 MHz, $CDCl_3$)

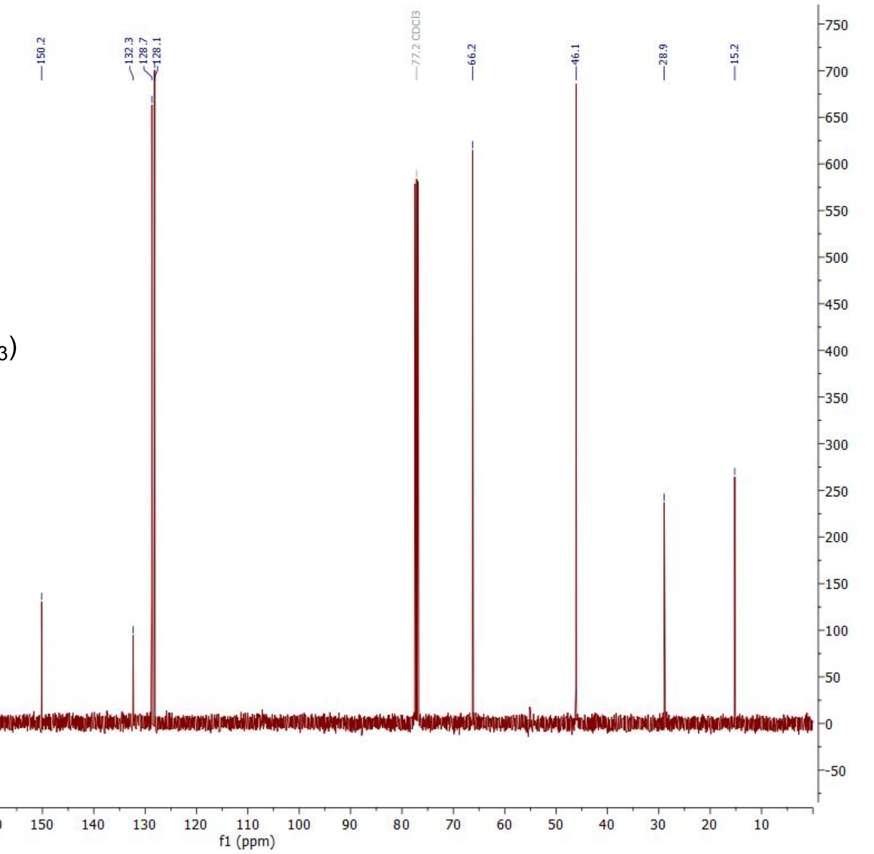

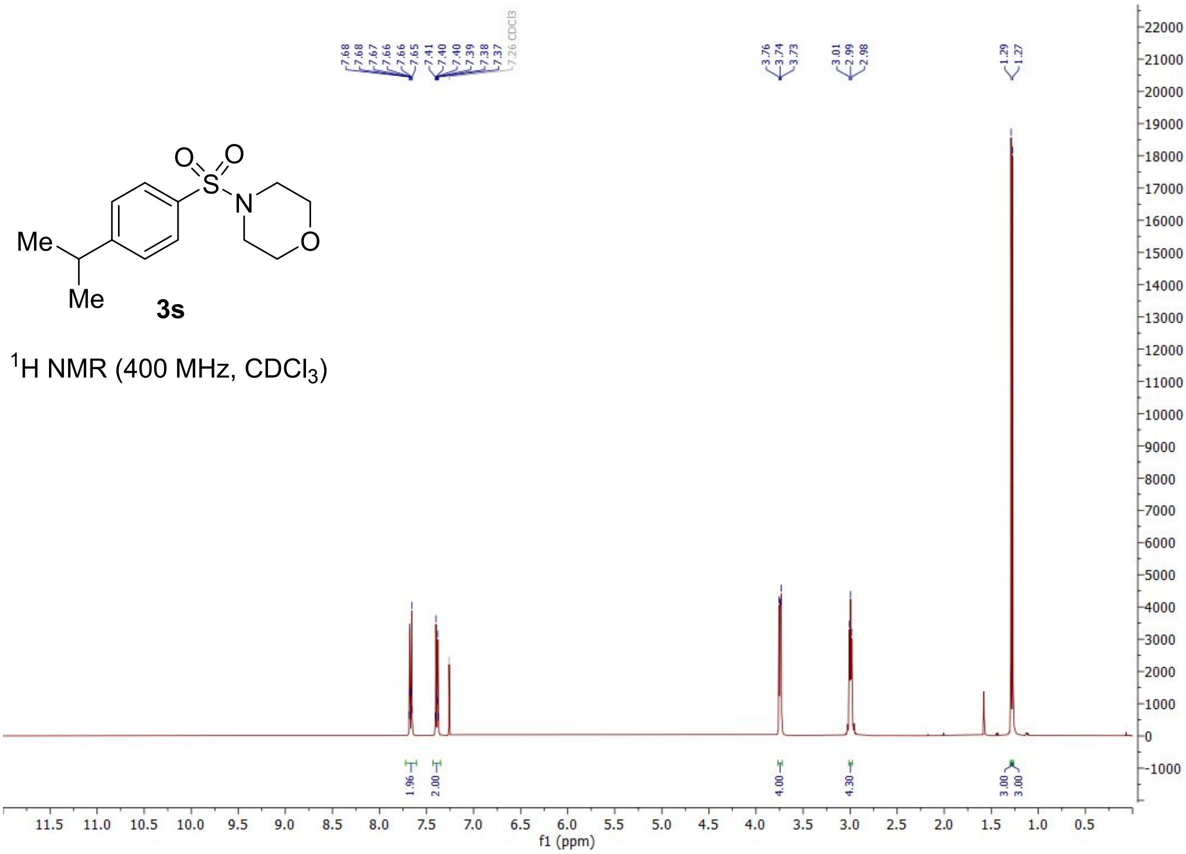
3s
^{1}H NMR (400 MHz, CDCl$_3$)
7.68 7.68 7.67 7.66 7.66 7.65 7.41 7.40 7.40 7.39 7.38 7.37 7.26 CDCl3
3.76 3.74 3.73
3.01 2.99 2.98
1.29 1.27
1.96 2.00 4.00 4.30 3.00 3.00
f1 (ppm)

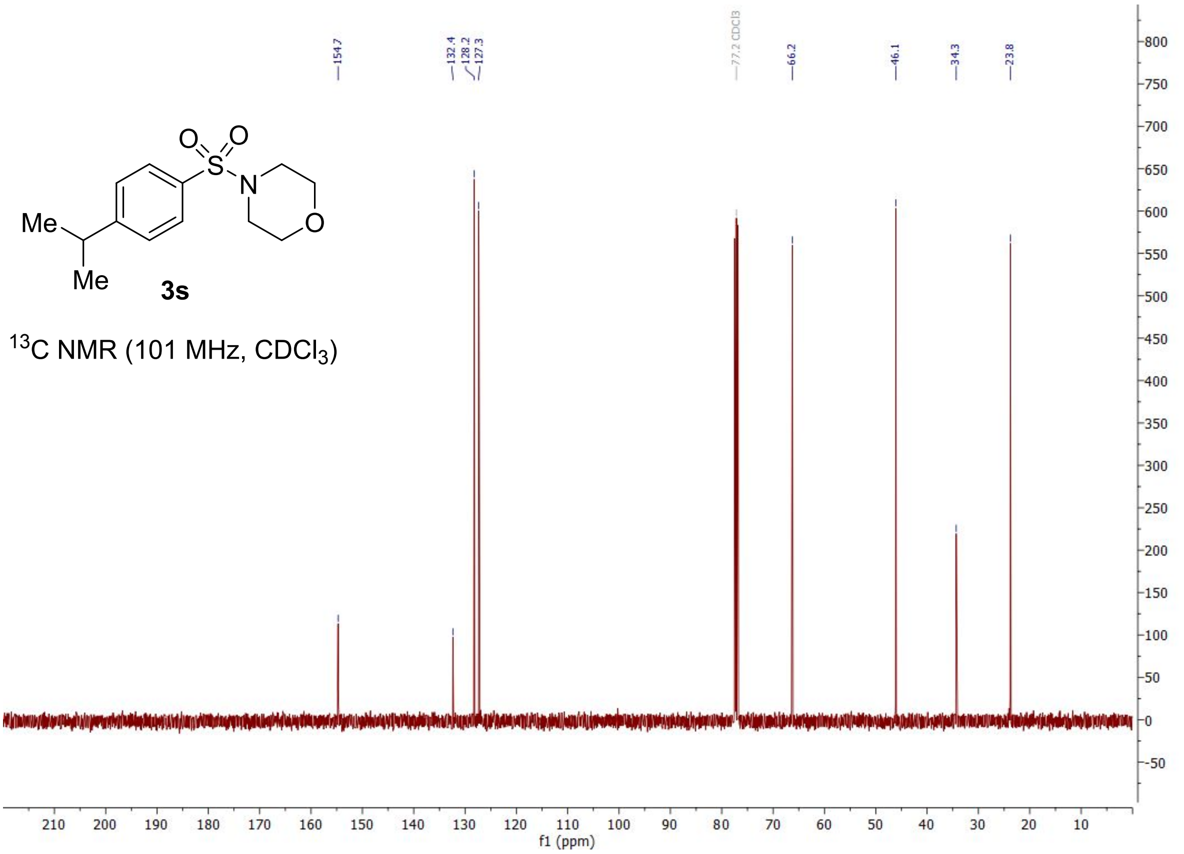
3s
^{13}C NMR (101 MHz, CDCl$_3$)
154.7
132.4 128.2 127.3
77.2 CDCl3
66.2
46.1
34.3
23.8
f1 (ppm)

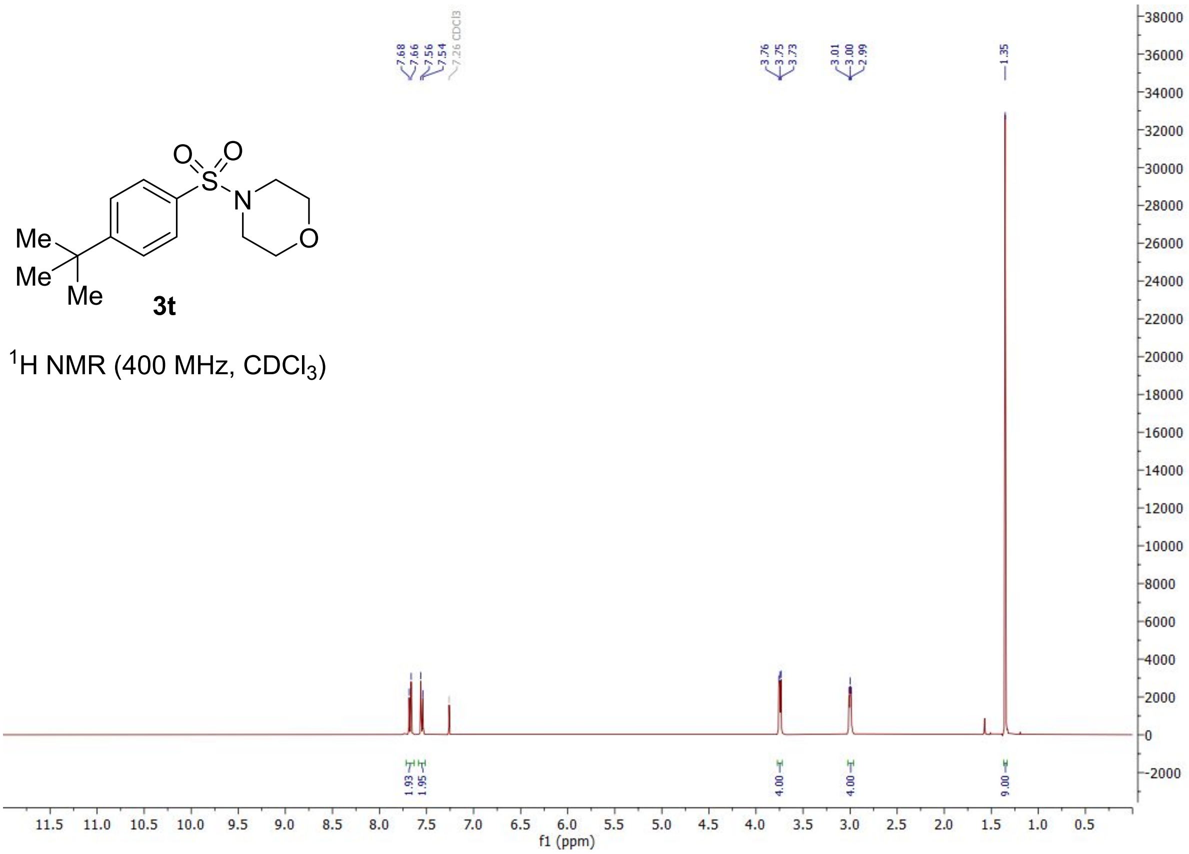

Me
Me
Me
3t
^{1}H NMR (400 MHz, CDCl$_3$)
7.68
7.66
7.56
7.54
7.26 CDCl3
3.76
3.75
3.73
3.01
3.00
2.99
1.35
1.93
1.95
4.00
4.00
9.00
f1 (ppm)

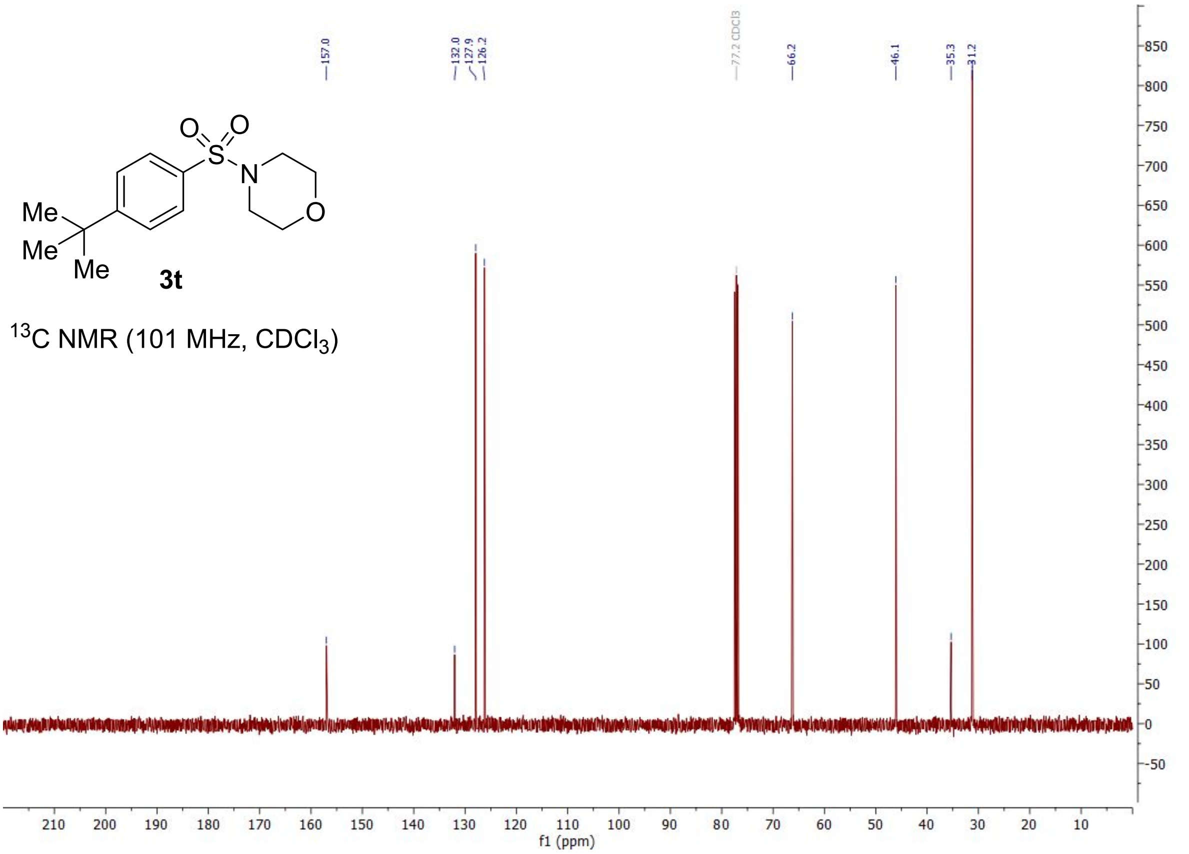

Me
Me
Me
3t
^{13}C NMR (101 MHz, CDCl$_3$)
157.0
132.0
127.9
126.2
77.2 CDCl3
66.2
46.1
35.3
31.2
f1 (ppm)

3u

^{1}H NMR (400 MHz, $CDCl_3$)

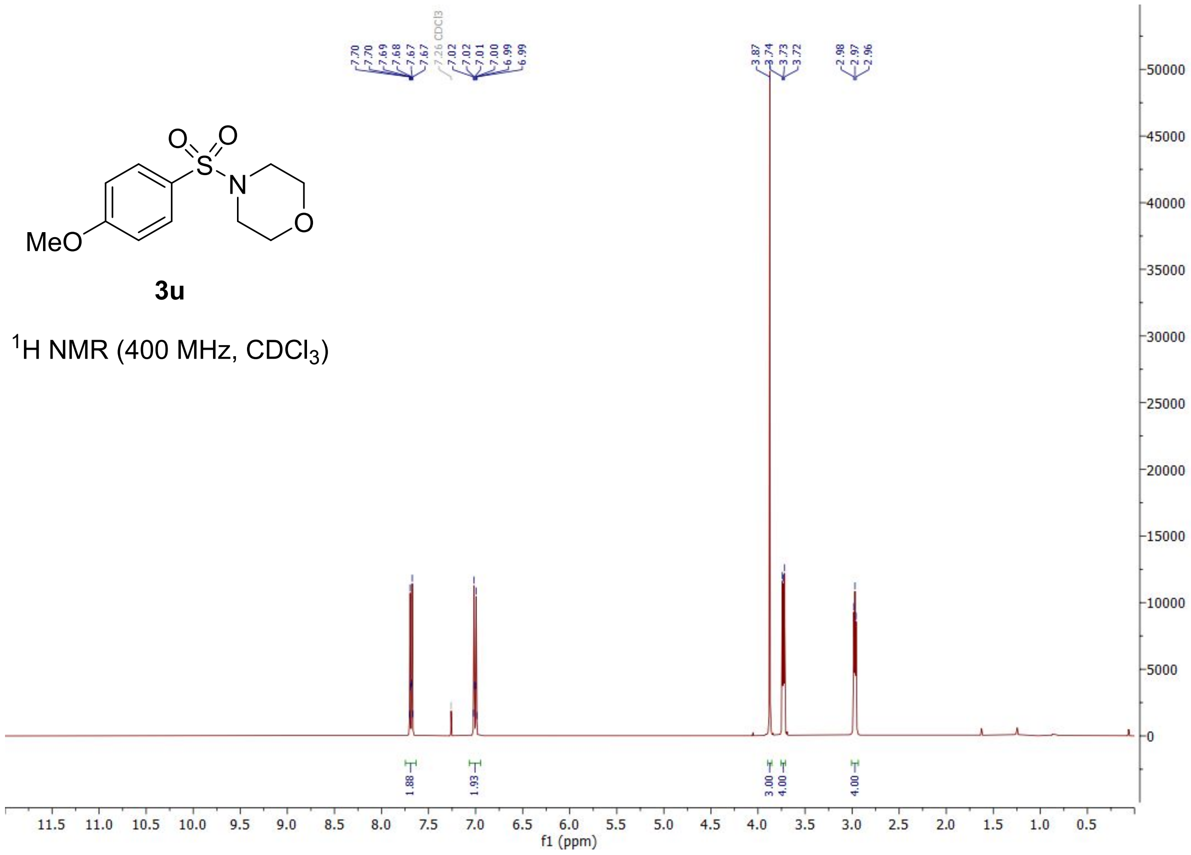

3u

^{13}C NMR (101 MHz, $CDCl_3$)

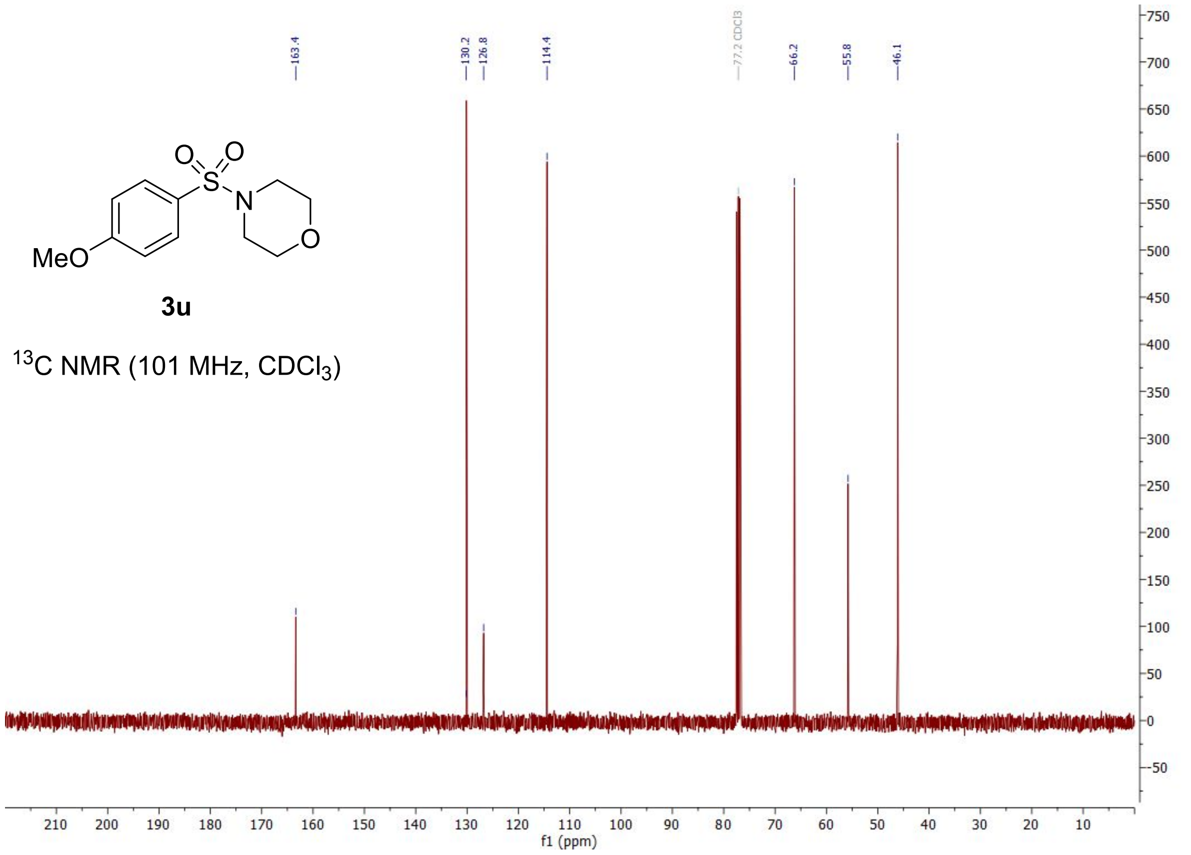

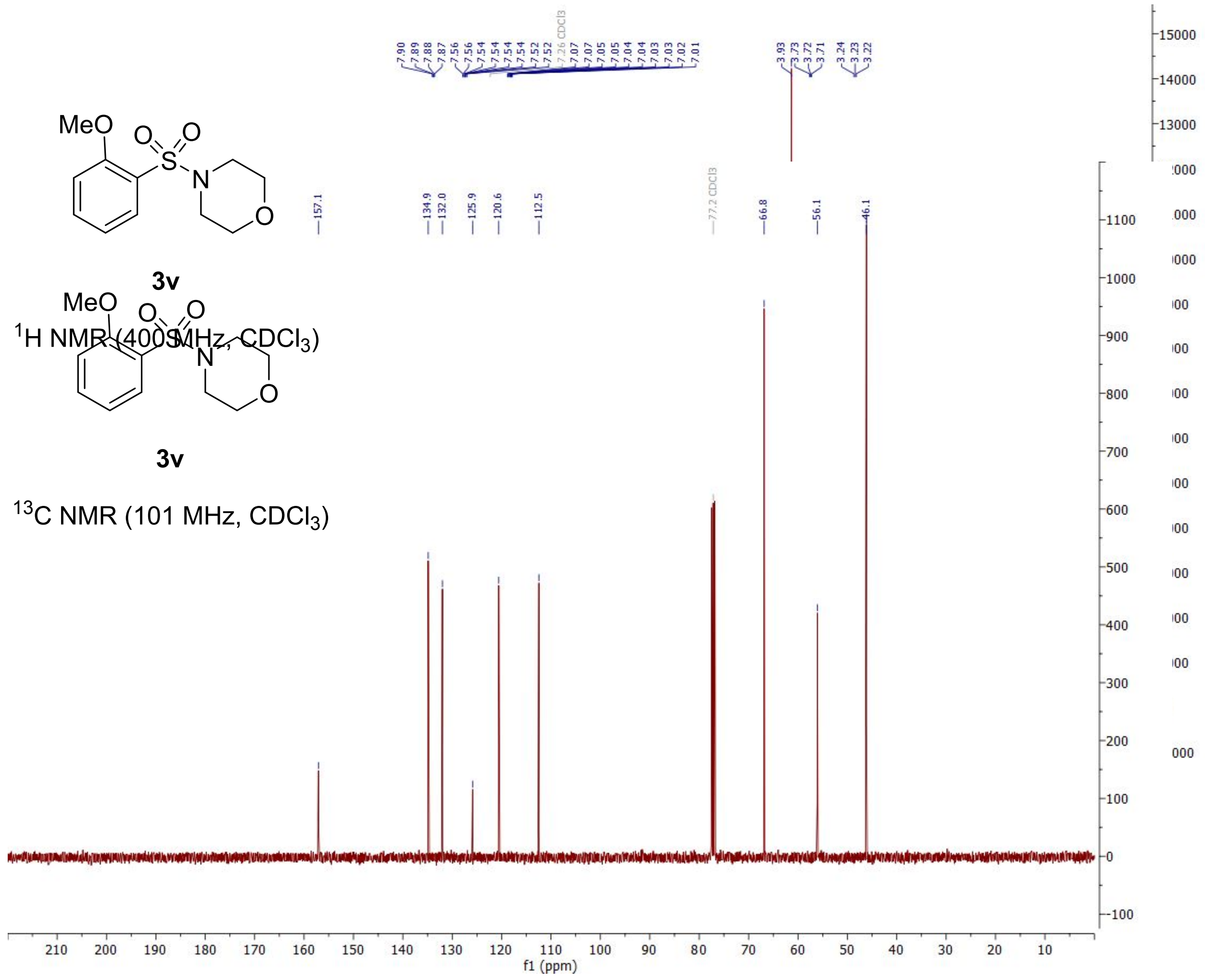

MeO
3v
^{1}H NMR (400 MHz, CDCl$_3$)
MeO
3v
^{13}C NMR (101 MHz, CDCl$_3$)
157.1
134.9
132.0
125.9
120.6
112.5
77.2 CDCl3
66.8
56.1
46.1
f1 (ppm)

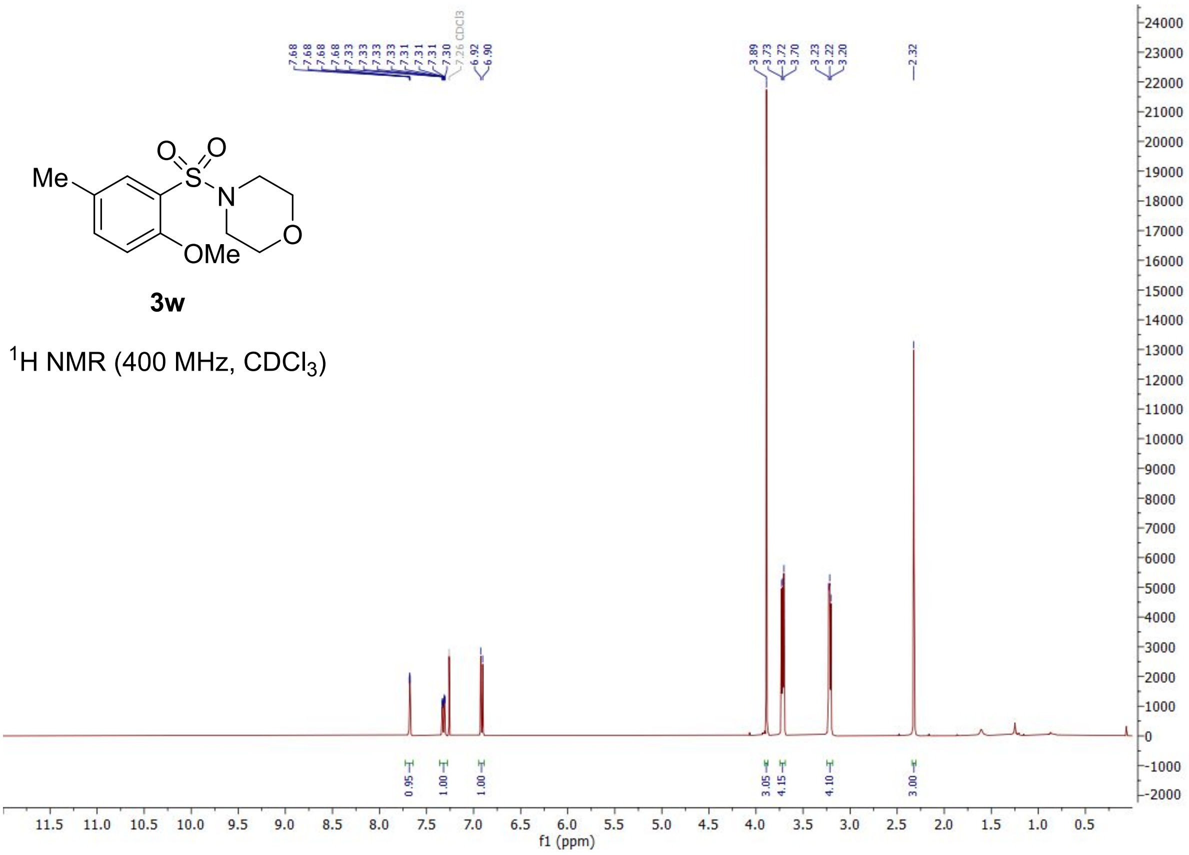
3w
1H NMR (400 MHz, CDCl3)
f1 (ppm)

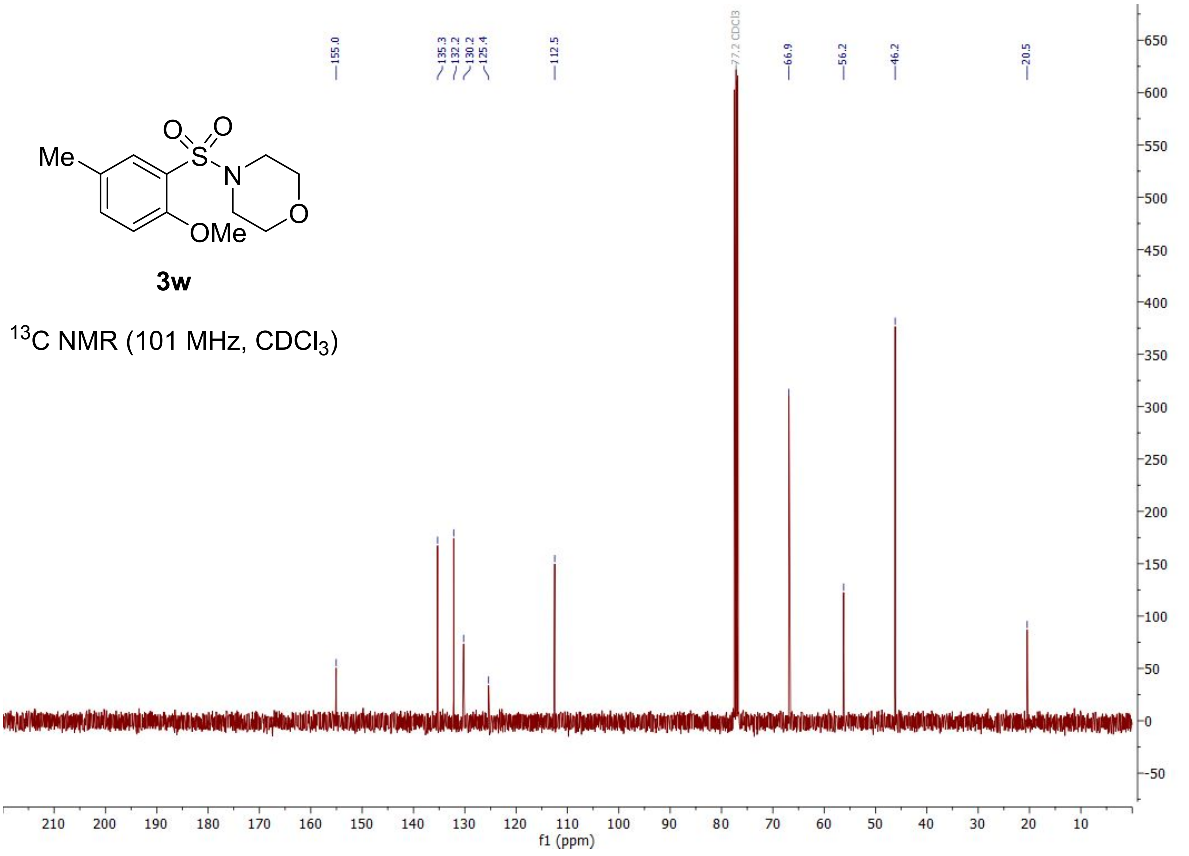
3w
13C NMR (101 MHz, CDCl3)
f1 (ppm)

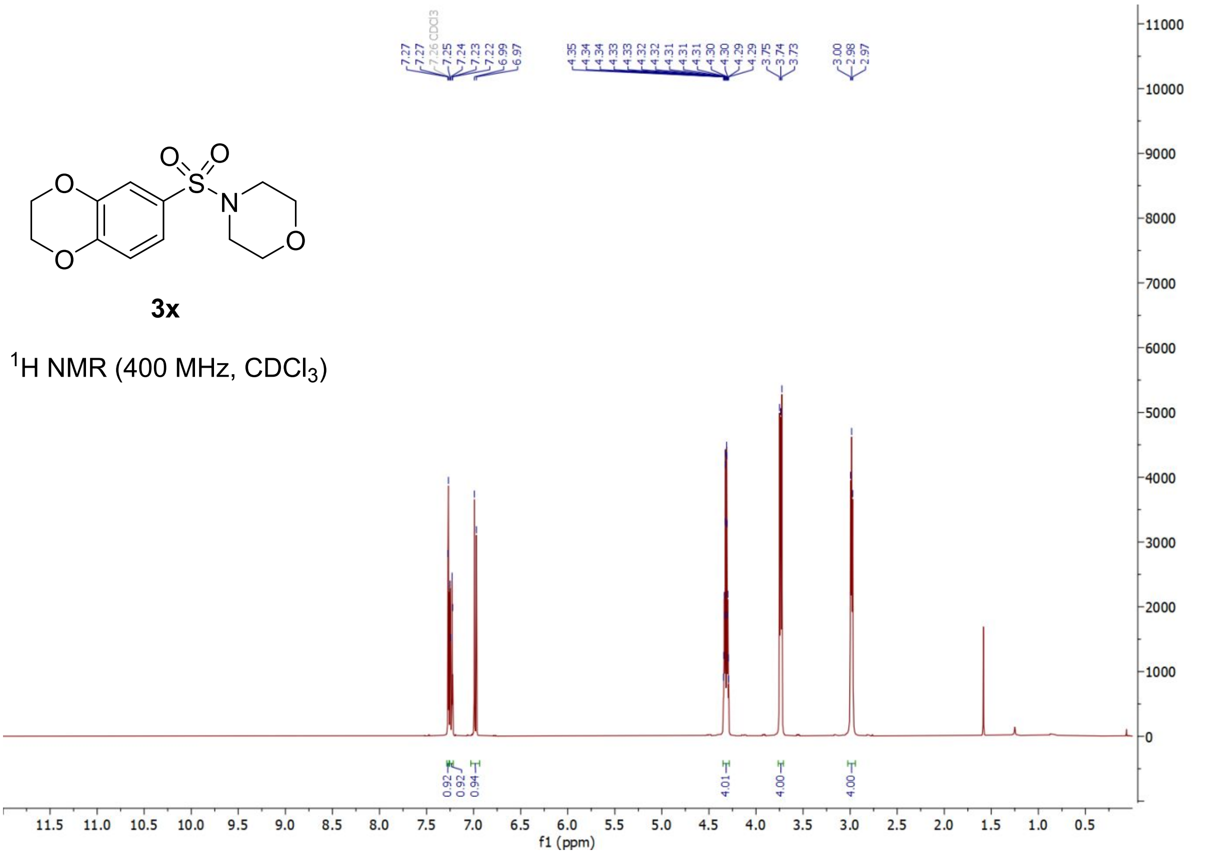
7.27
7.27
7.26 CDCl3
7.25
7.24
7.23
7.22
6.99
6.97
4.35
4.34
4.34
4.33
4.33
4.32
4.32
4.31
4.31
4.31
4.30
4.30
4.29
4.29
3.75
3.74
3.73
3.00
2.98
2.97
3x
1H NMR (400 MHz, CDCl3)
0.92
0.92
0.94
4.01
4.00
4.00
f1 (ppm)

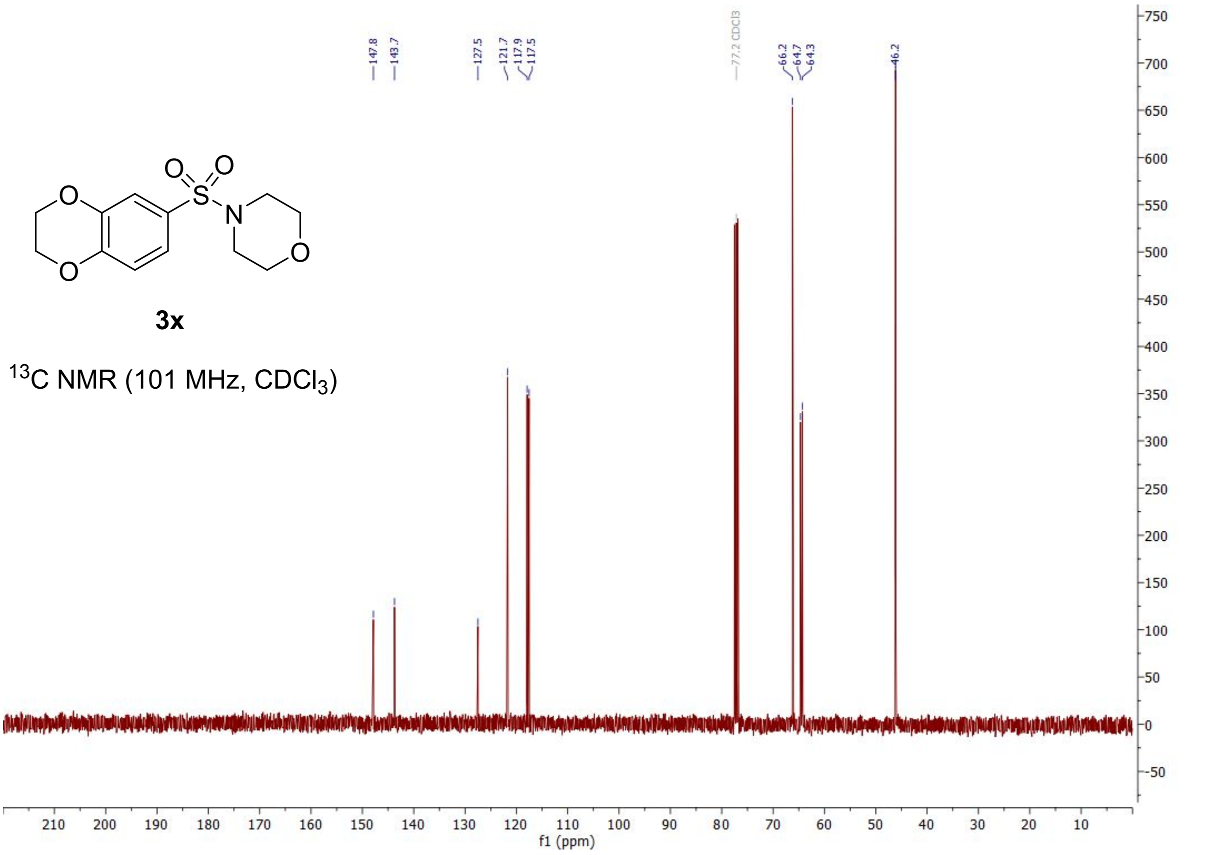
147.8
143.7
127.5
121.7
117.9
117.5
77.2 CDCl3
66.2
64.7
64.3
46.2
3x
13C NMR (101 MHz, CDCl3)
f1 (ppm)

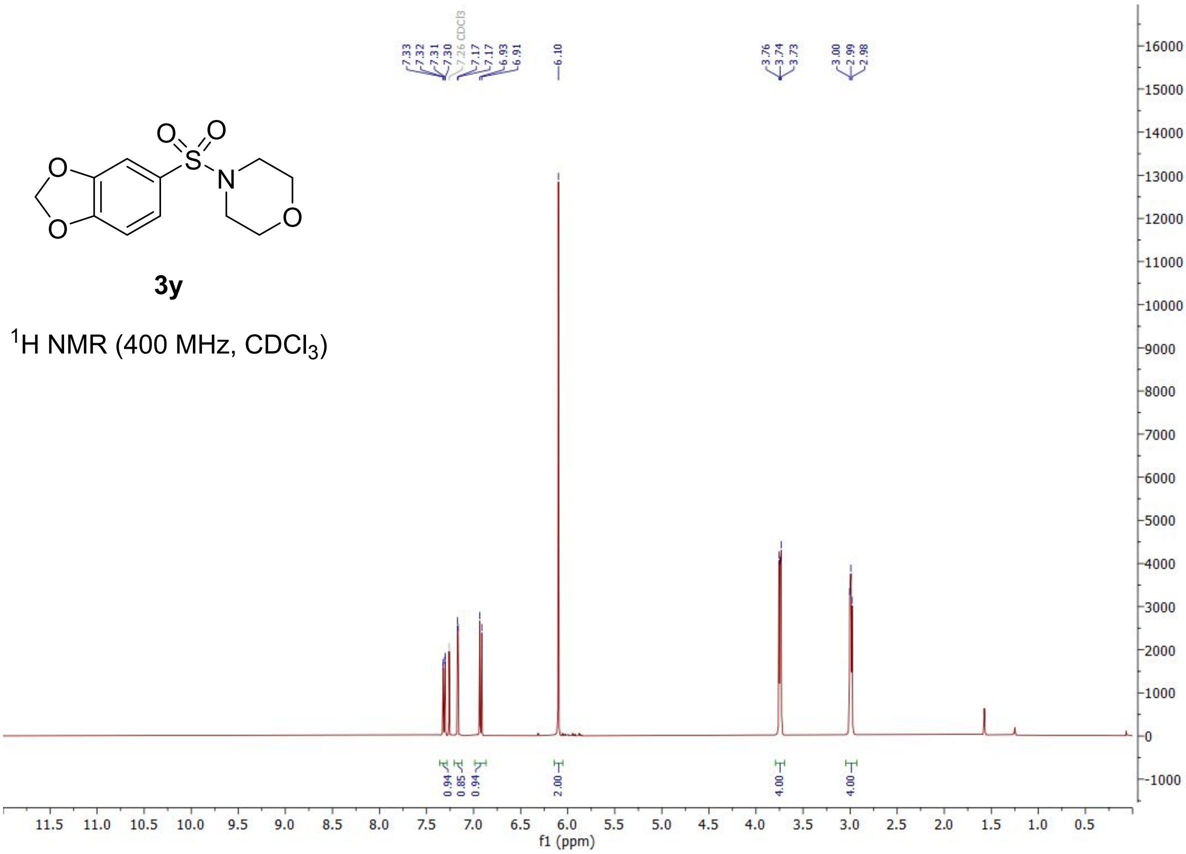

3y

^{1}H NMR (400 MHz, $CDCl_3$)

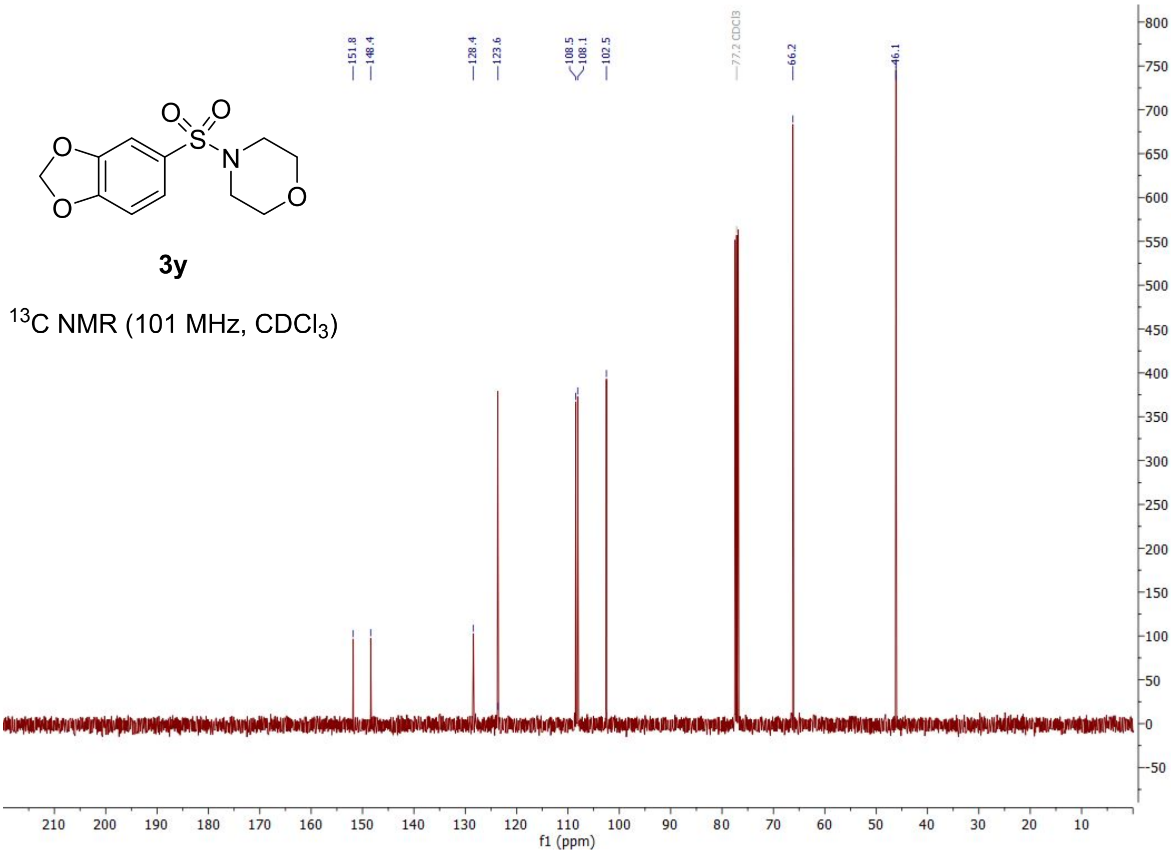

3y

^{13}C NMR (101 MHz, $CDCl_3$)

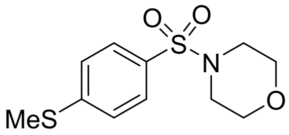

3z

^{1}H NMR (400 MHz, $CDCl_3$)

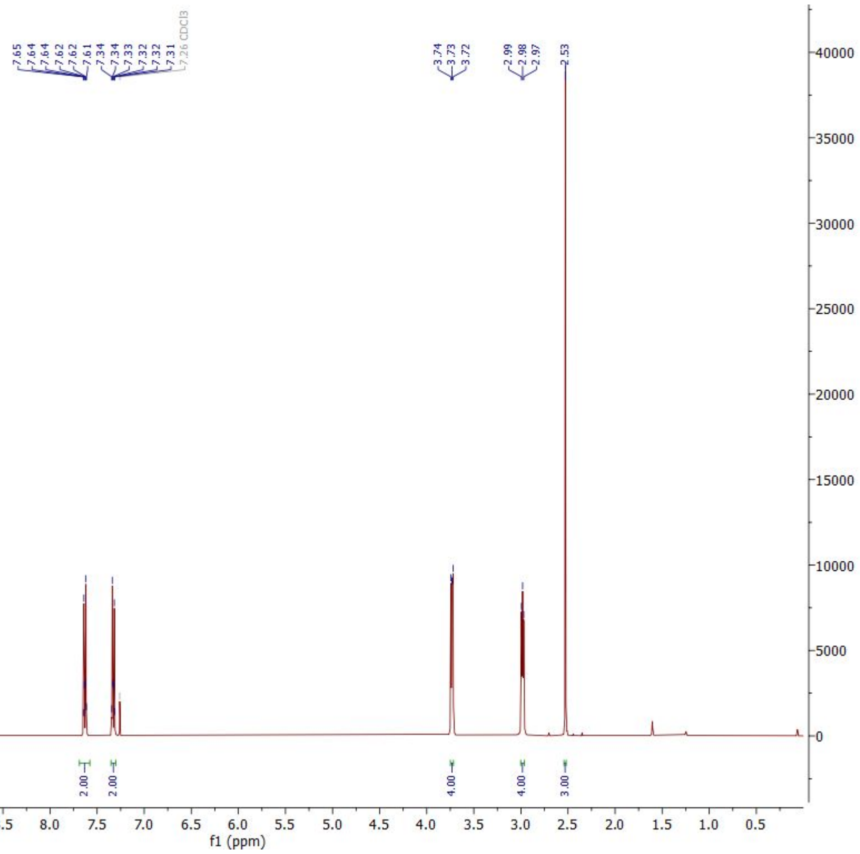

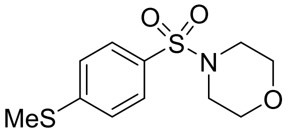

3z

^{13}C NMR (101 MHz, $CDCl_3$)

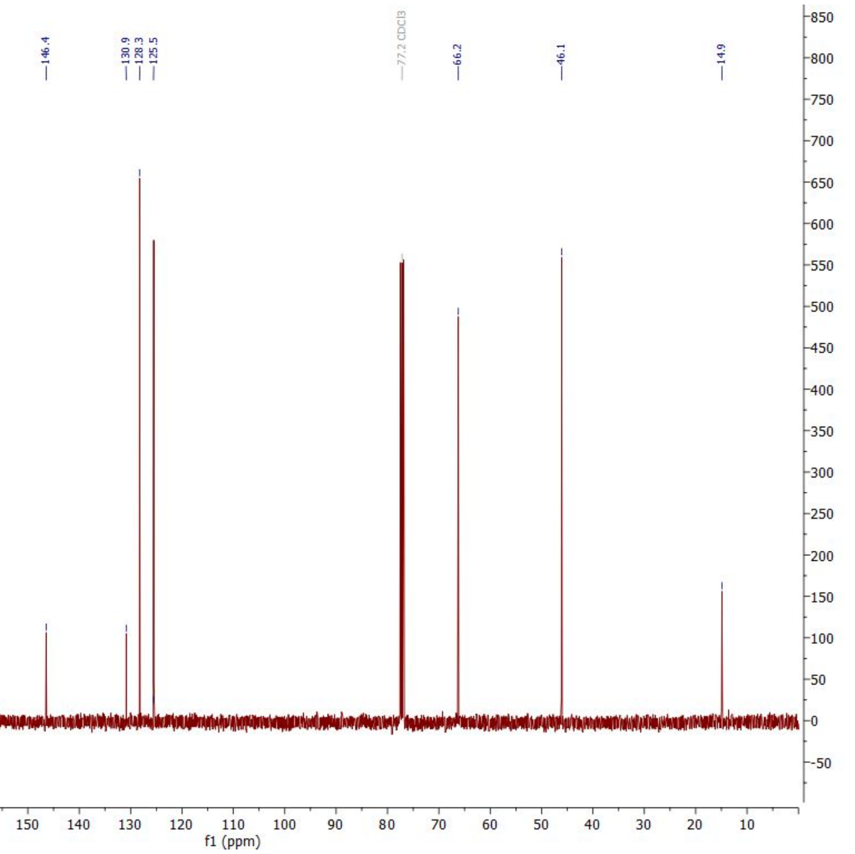

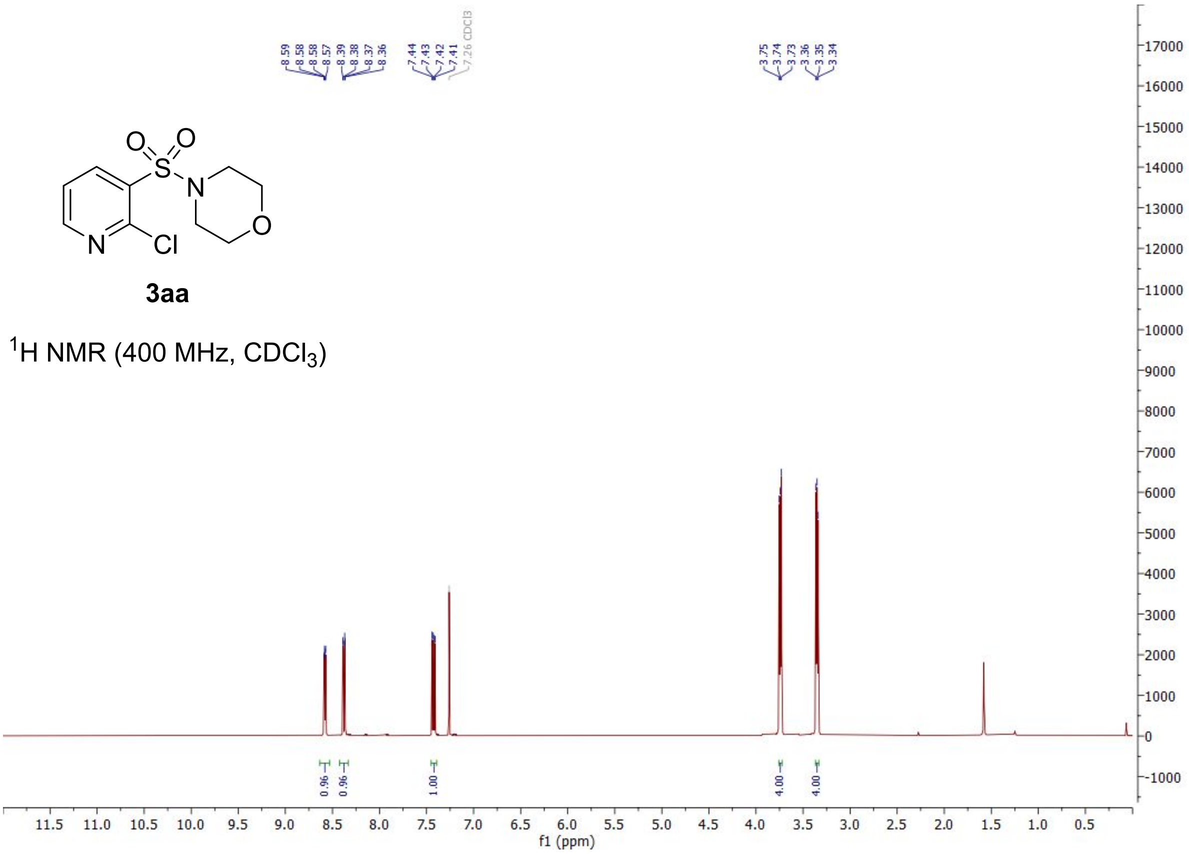

3aa

^{1}H NMR (400 MHz, $CDCl_3$)

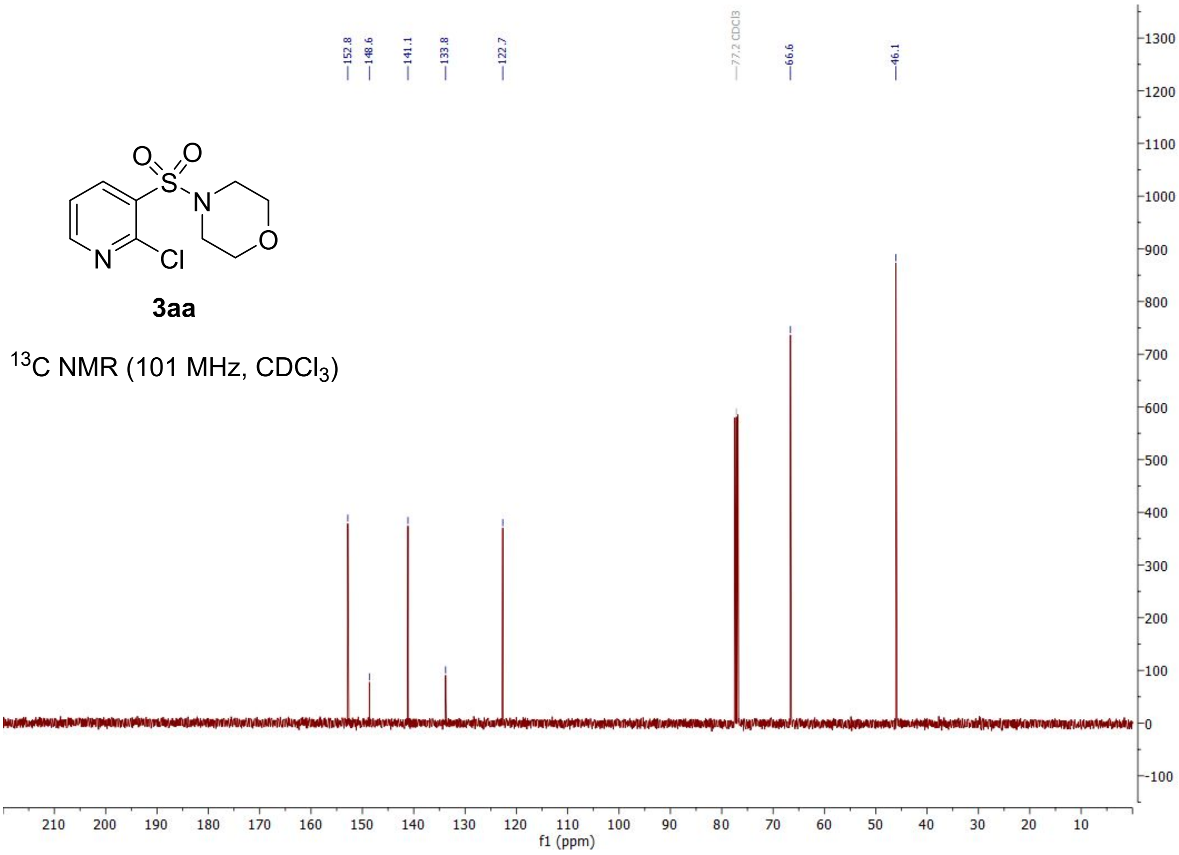

3aa

^{13}C NMR (101 MHz, $CDCl_3$)

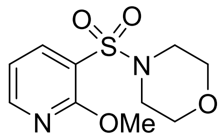

3ab

^{1}H NMR (400 MHz, $CDCl_3$)

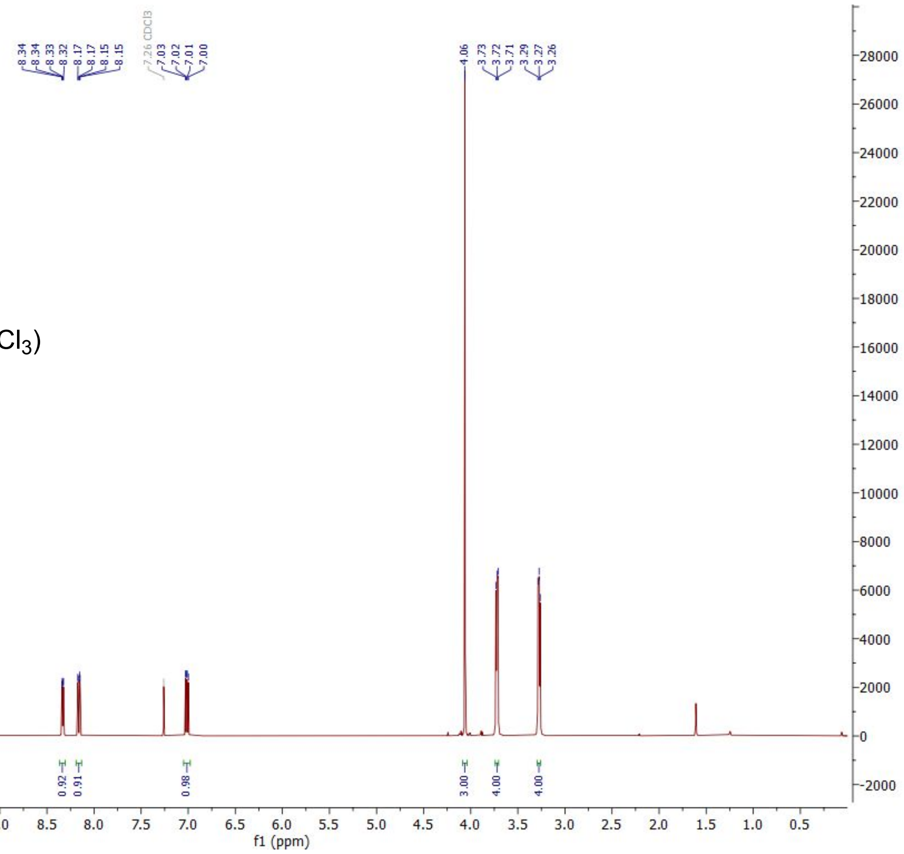

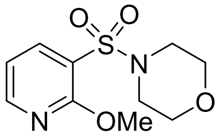

3ab

^{13}C NMR (101 MHz, $CDCl_3$)

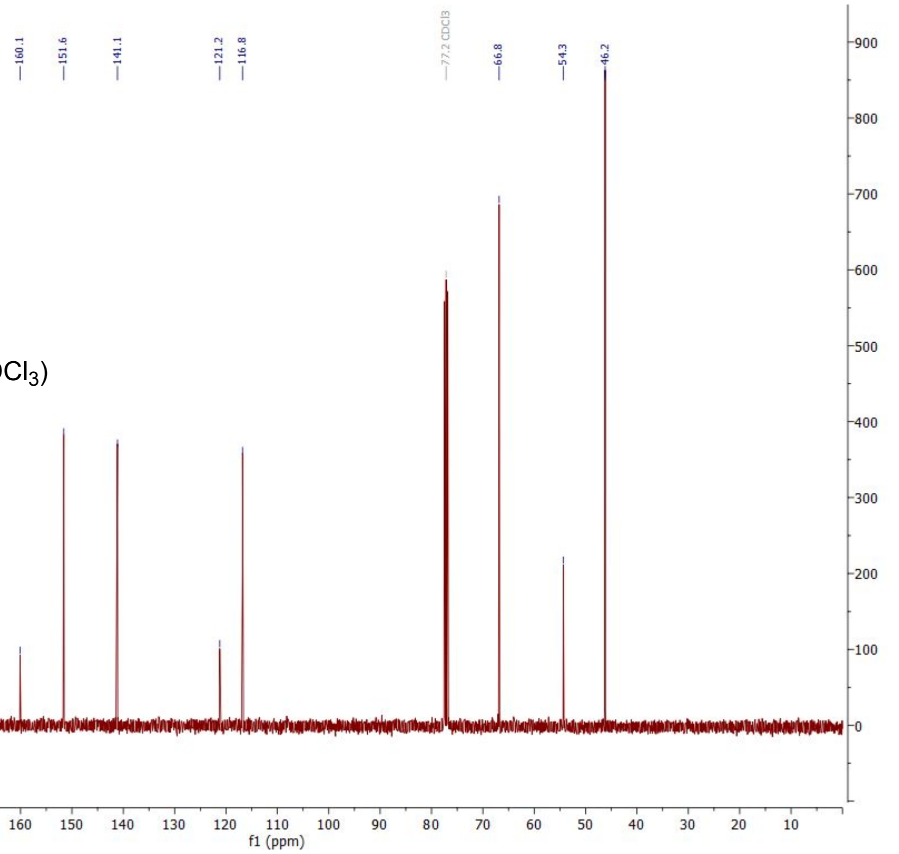

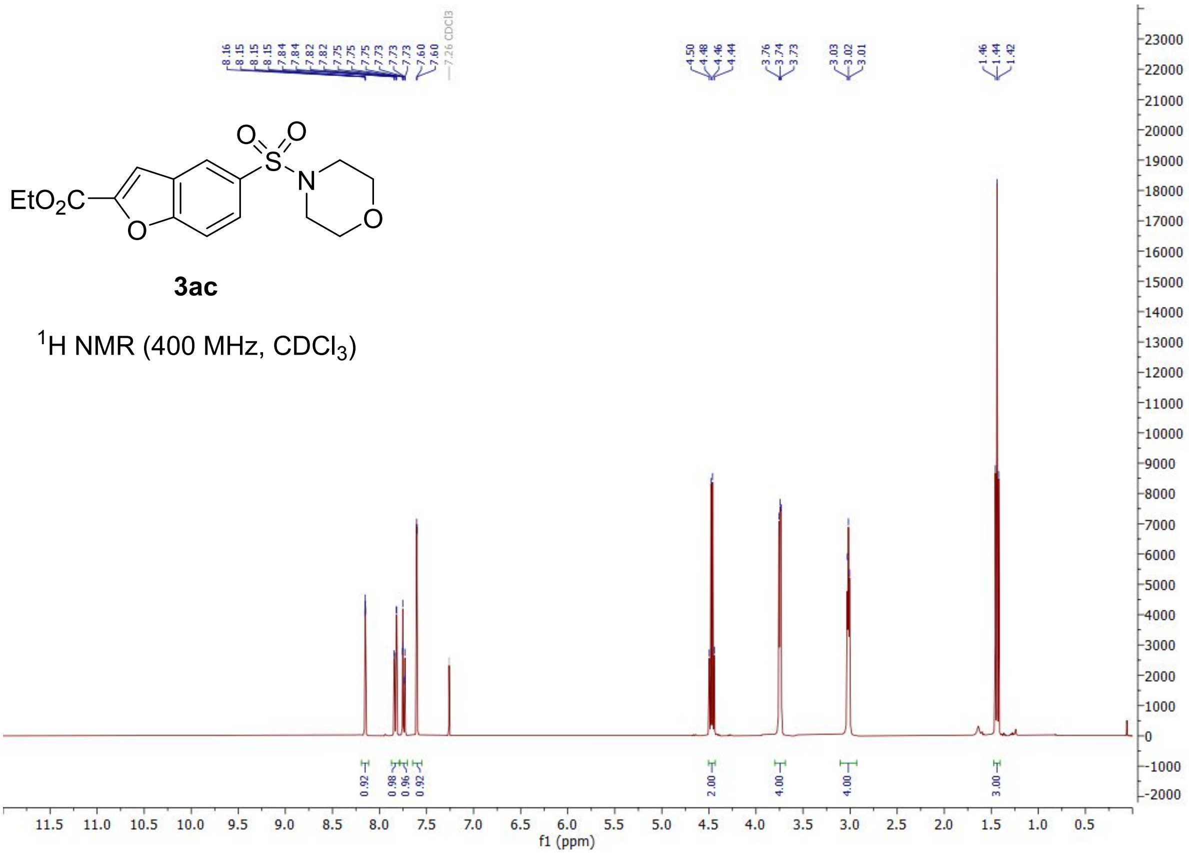
8.16
8.15
8.15
8.15
7.84
7.84
7.82
7.82
7.75
7.75
7.75
7.75
7.73
7.73
7.73
7.60
7.60
7.26 CDCl3
4.50
4.48
4.46
4.44
3.76
3.74
3.73
3.03
3.02
3.01
1.46
1.44
1.42
EtO2C
O
O
S
O
N
O
3ac
1H NMR (400 MHz, CDCl3)
0.92
0.98
0.96
0.92
2.00
4.00
4.00
3.00
f1 (ppm)

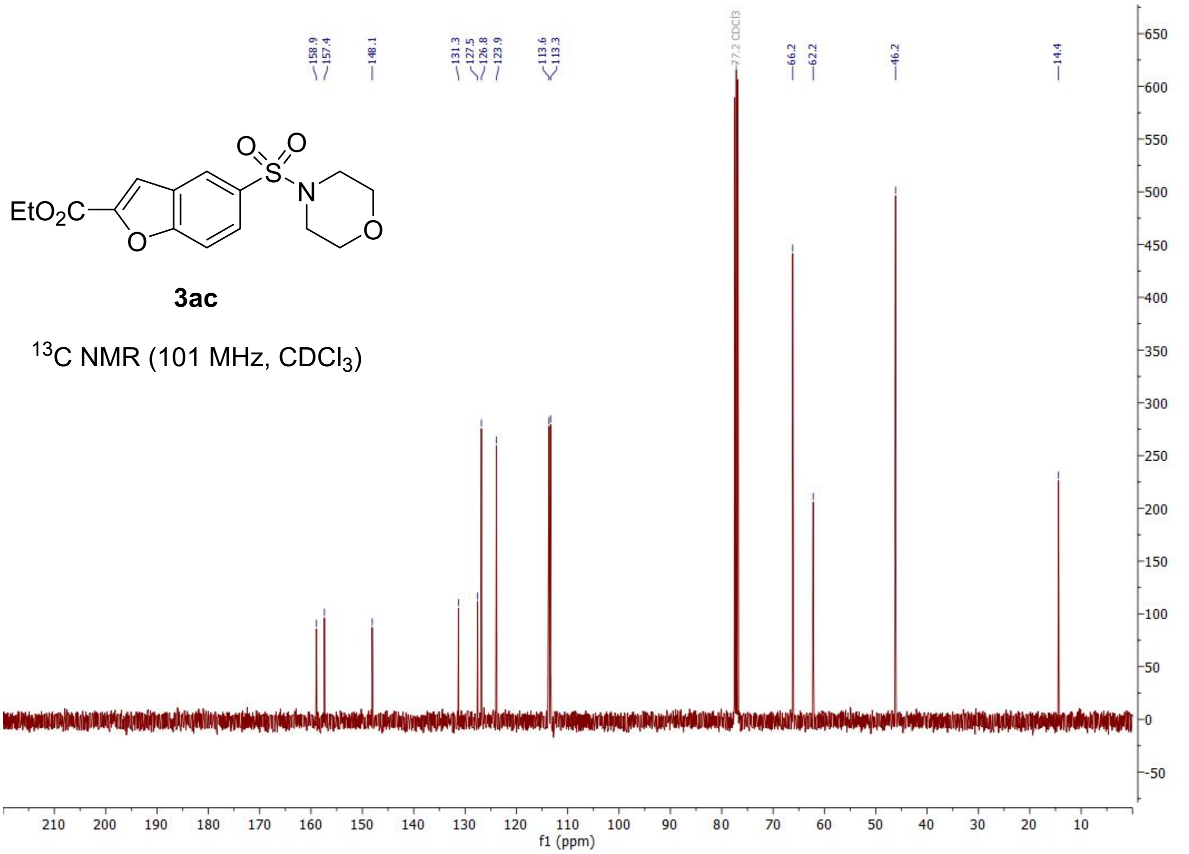
158.9
157.4
148.1
131.3
127.5
126.8
123.9
113.6
113.3
77.2 CDCl3
66.2
62.2
46.2
14.4
EtO2C
O
O
S
O
N
O
3ac
13C NMR (101 MHz, CDCl3)
f1 (ppm)

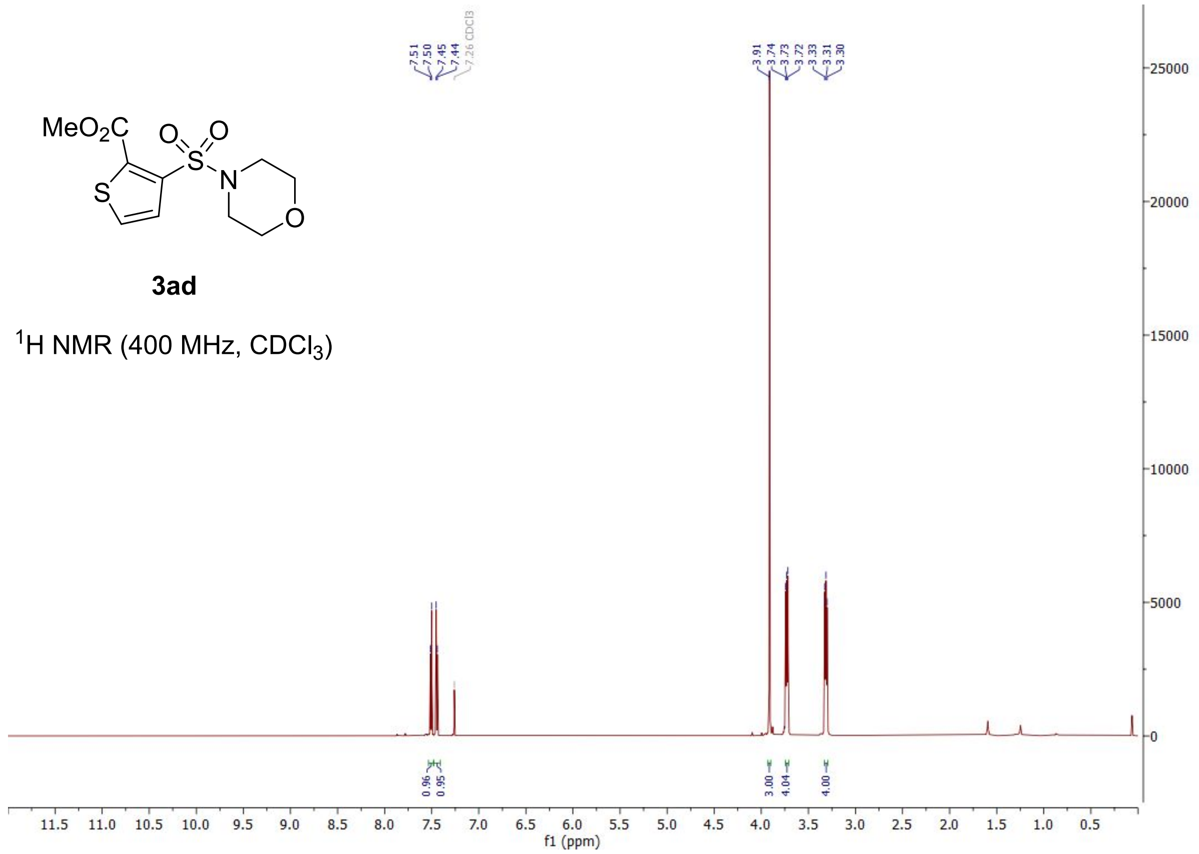
7.51
7.50
7.45
7.44
7.26 CDCl3
3.91
3.74
3.73
3.72
3.33
3.31
3.30
MeO2C
S
N
O
3ad
1H NMR (400 MHz, CDCl3)
0.96
0.95
3.00
4.04
4.00
f1 (ppm)

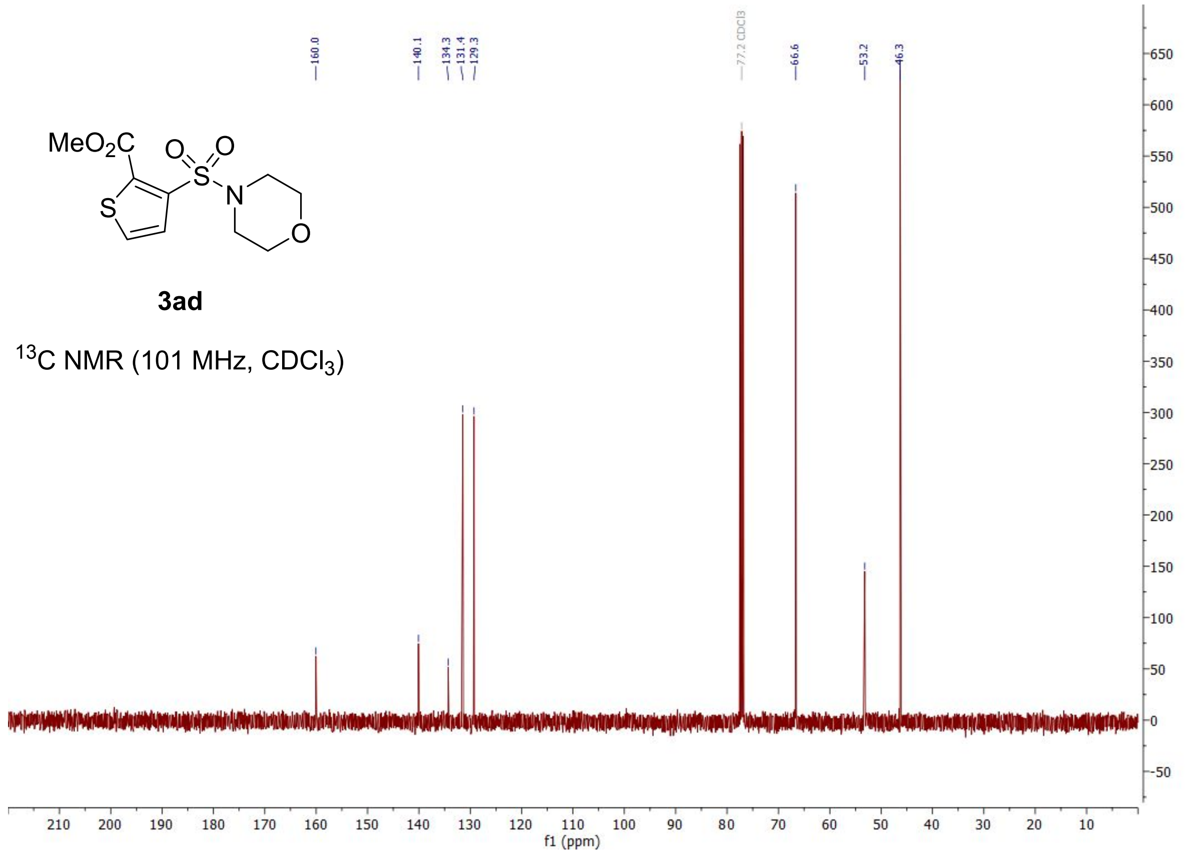
160.0
140.1
134.3
131.4
129.3
77.2 CDCl3
66.6
53.2
46.3
MeO2C
S
N
O
3ad
13C NMR (101 MHz, CDCl3)
f1 (ppm)

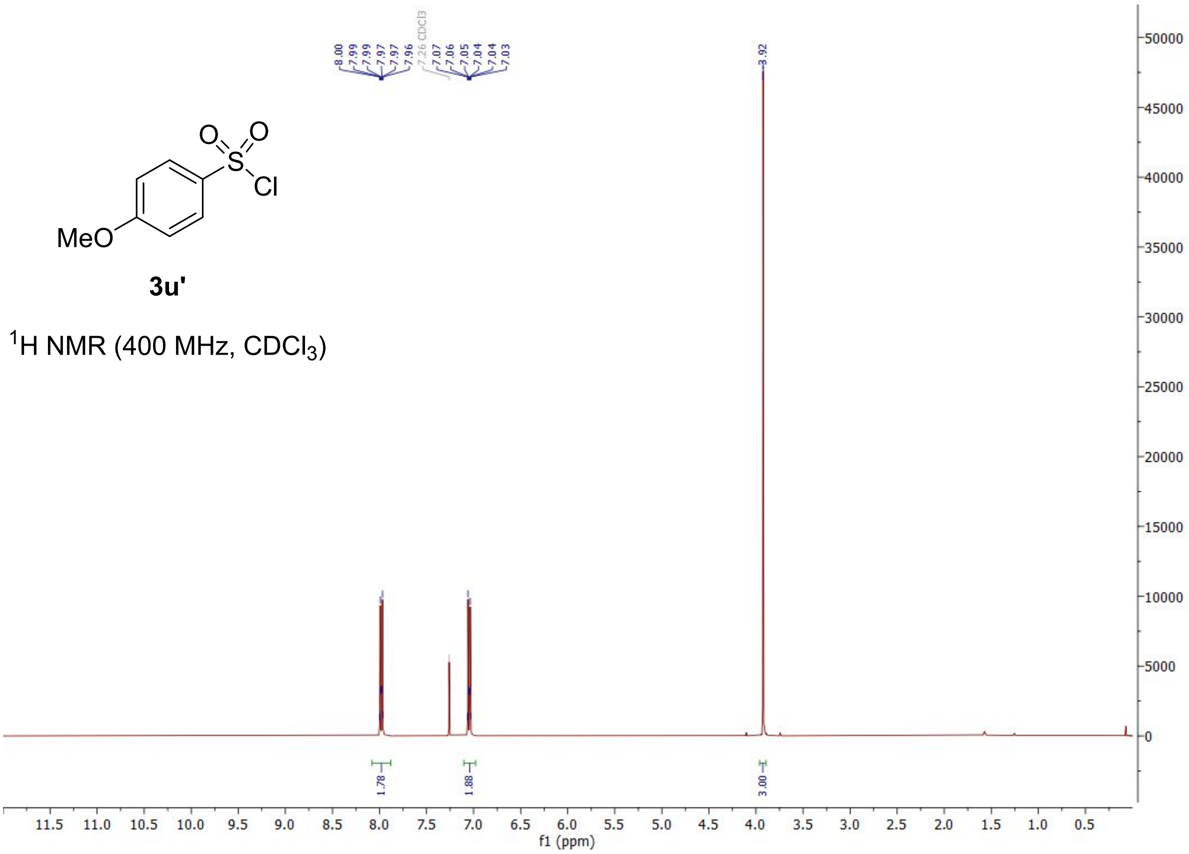

3u'

^{1}H NMR (400 MHz, $CDCl_3$)

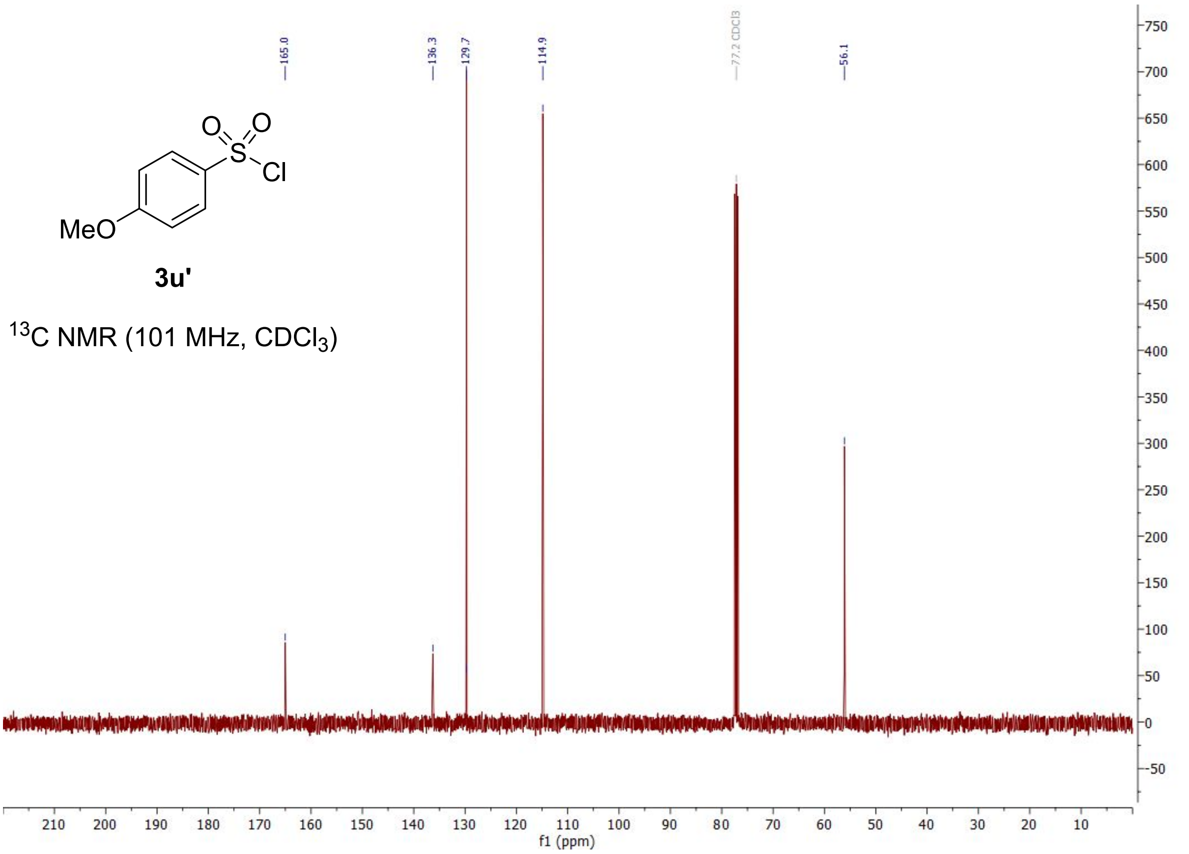

3u'

^{13}C NMR (101 MHz, $CDCl_3$)

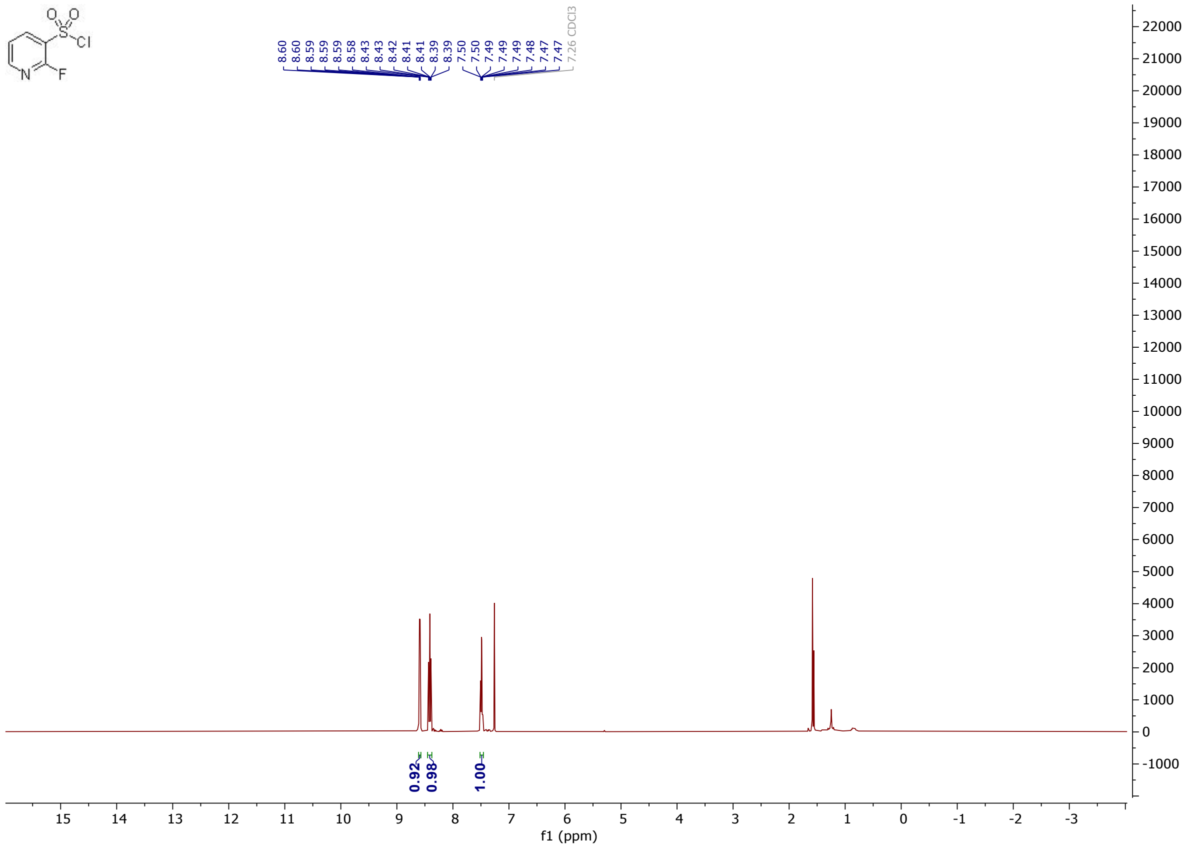

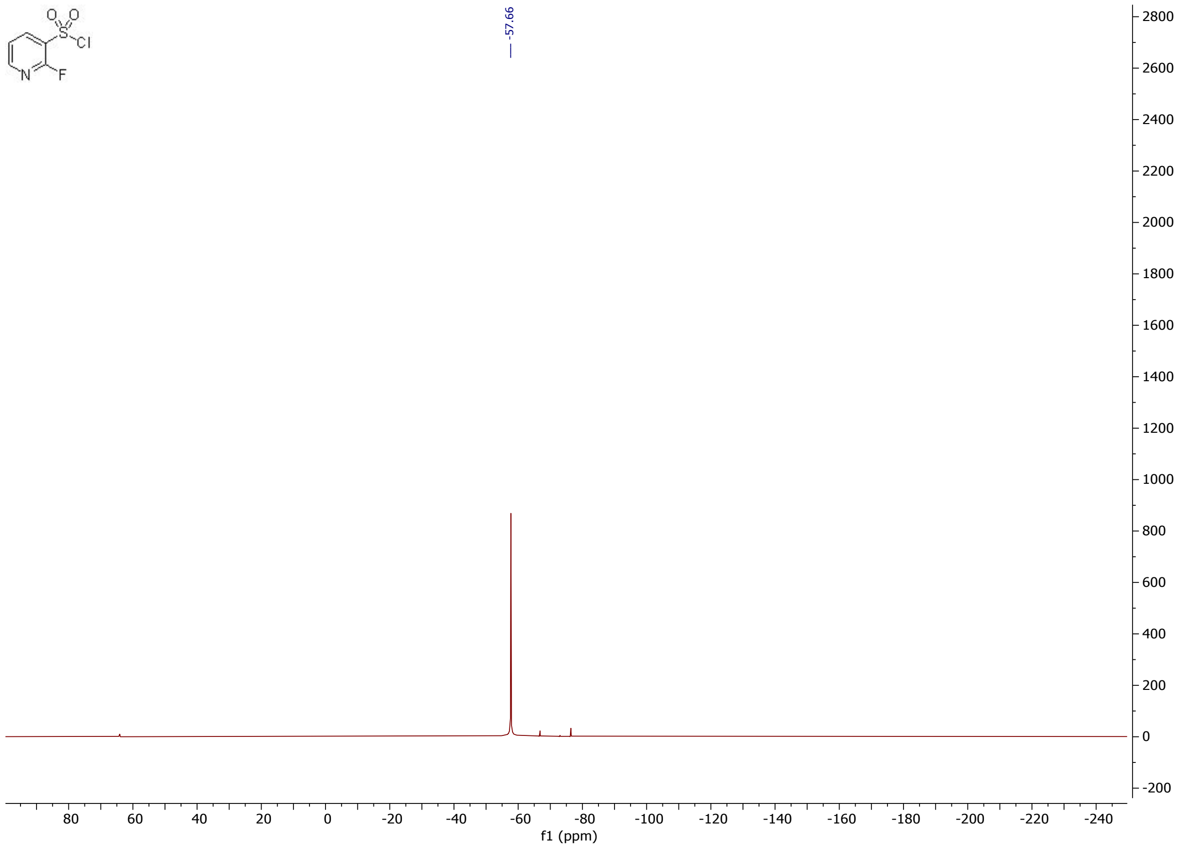

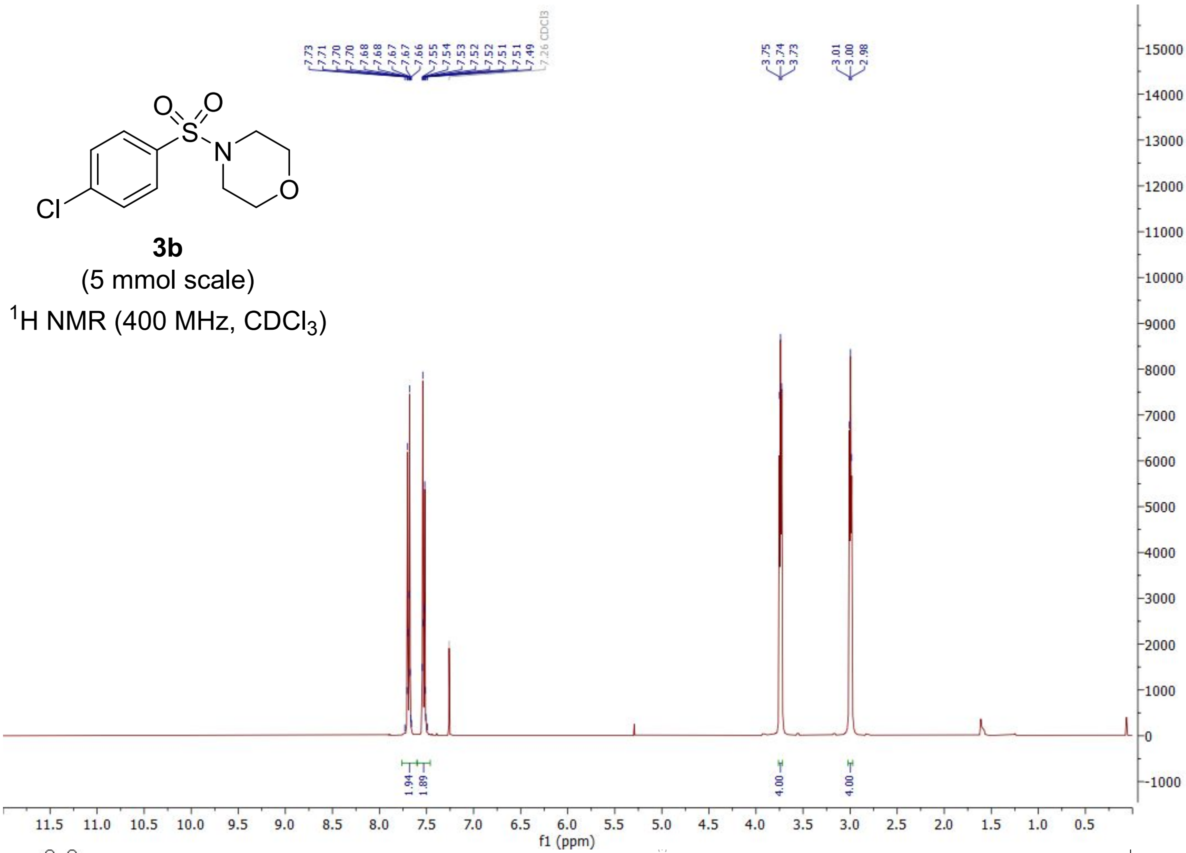
3b
(5 mmol scale)
^{1}H NMR (400 MHz, $CDCl_3$)
7.73 7.71 7.70 7.70 7.68 7.68 7.67 7.67 7.66 7.55 7.54 7.53 7.52 7.52 7.51 7.51 7.49
7.26 CDCl3
3.75 3.74 3.73
3.01 3.00 2.98
1.94 1.89 4.00 4.00
f1 (ppm)

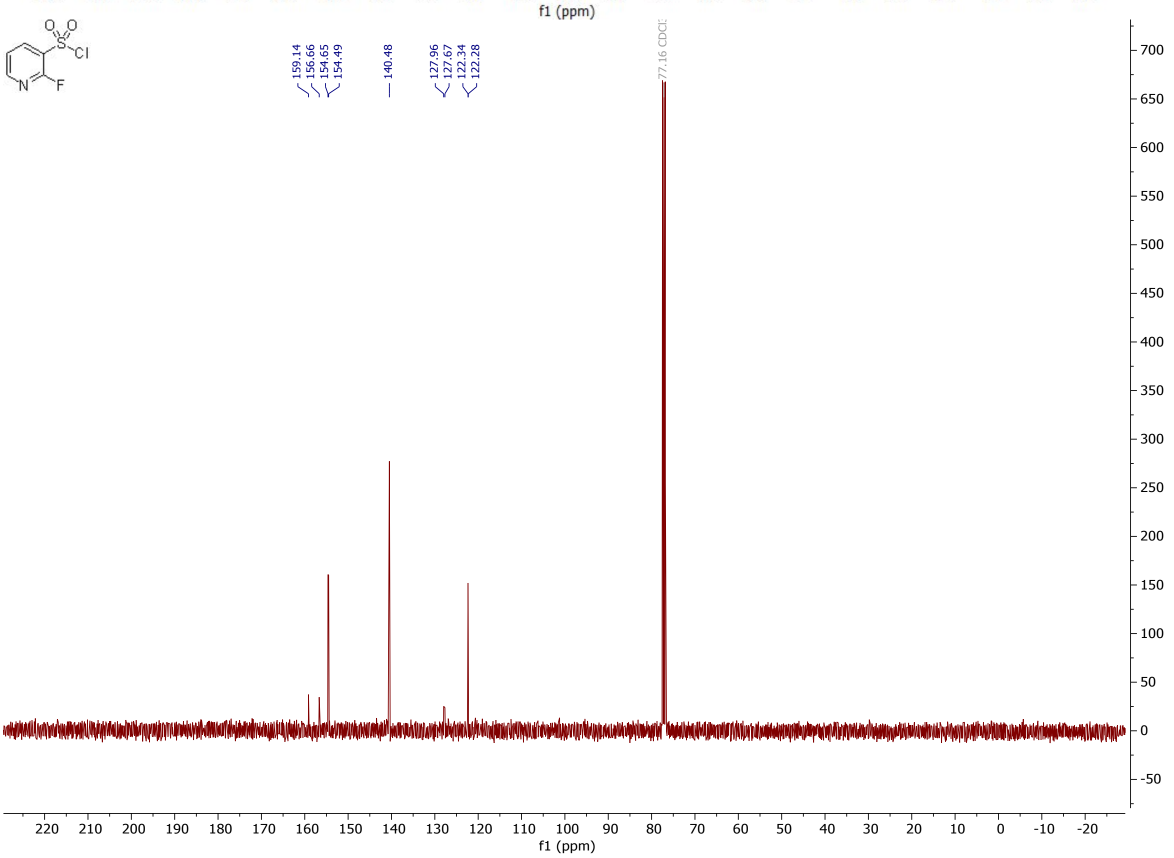
159.14 156.66 154.65 154.49
140.48
127.96 127.67 122.34 122.28
77.16 CDCl
f1 (ppm)

Scale-up

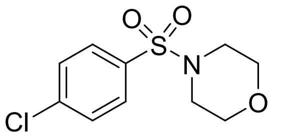

3b
(5 mmol scale)
^{13}C NMR (101 MHz, $CDCl_3$)

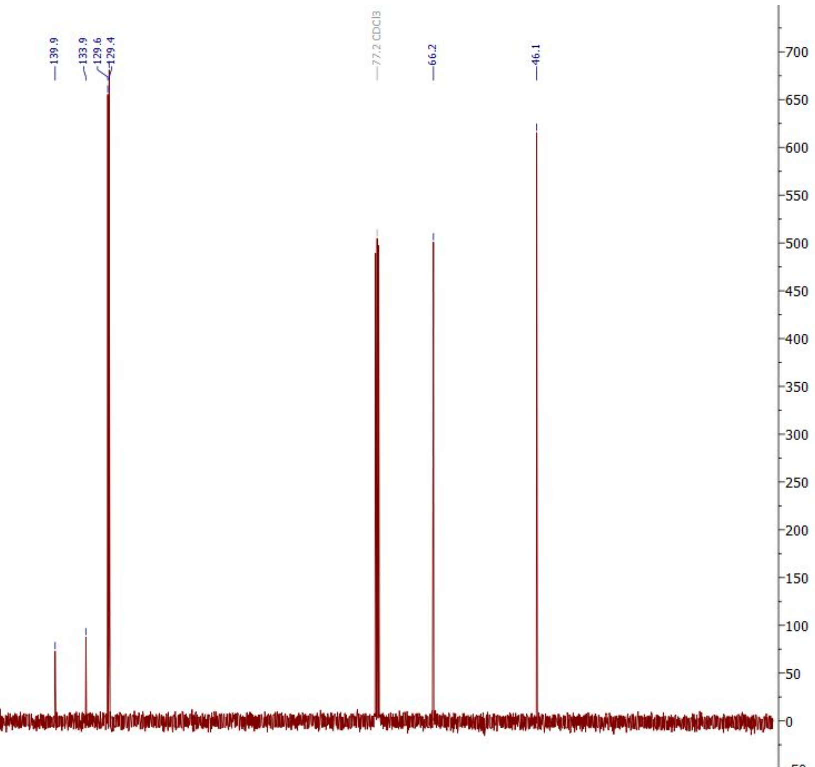

5. GCMS – 4-Methoxybenzenesulfonyl chloride (3u’)

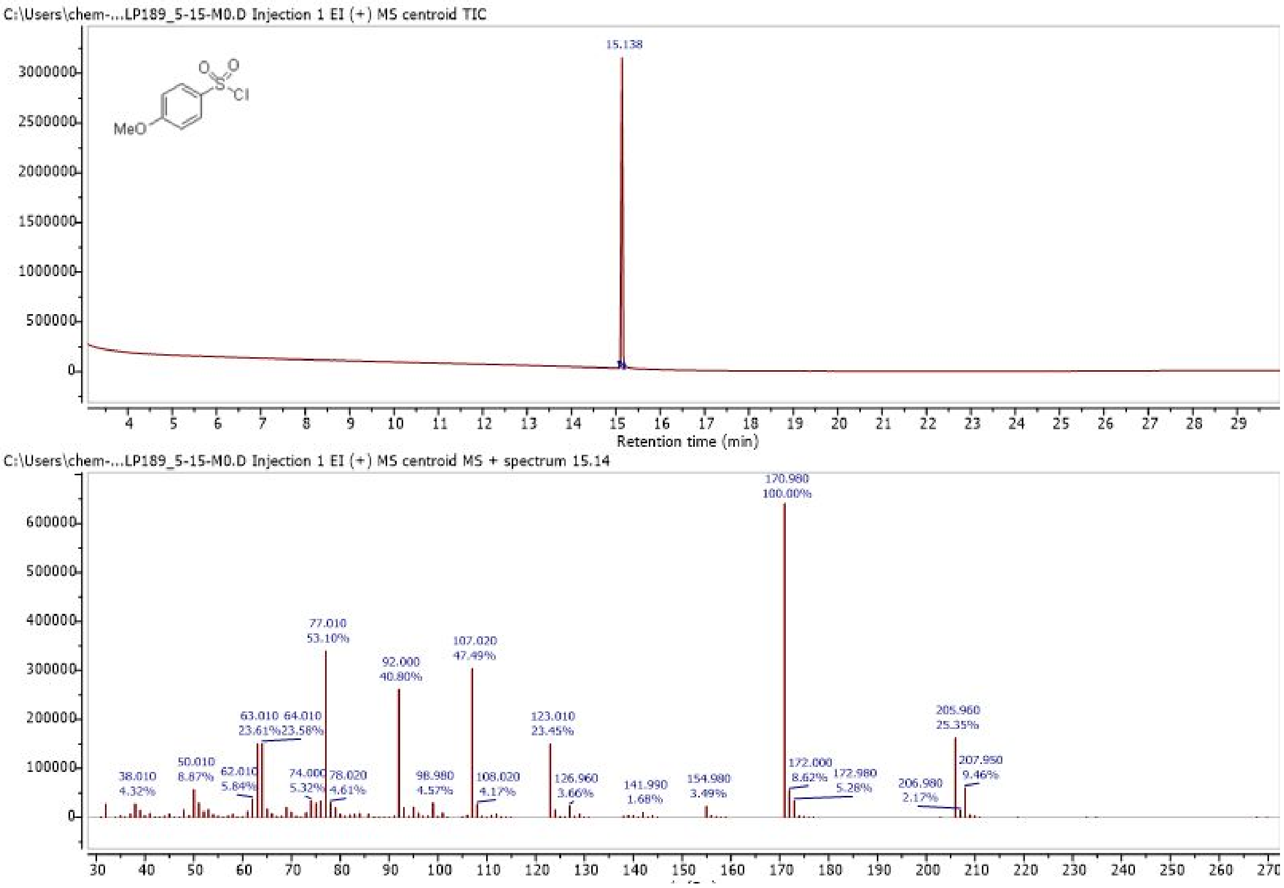

6. References

(1) Chen, Y.; Murray, P. R.; Davies, A. T.; Willis, M. C. Direct copper-catalyzed three-component synthesis of sulfonamides. *J. Am. Chem. Soc.* **2018**, *140* (28), 8781-8787.

(2) Flegeau, E. F.; Harrison, J. M.; Willis, M. C. One-Pot Sulfonamide synthesis exploiting the palladium-catalyzed sulfination of aryl iodides. *Synlett* **2015**, 101-105.

(3) Huang, H.-M.; Bellotti, P.; Erchinger, J. E.; Paulisch, T. O.; Glorius, F. Radical carbonyl umpolung arylation via dual nickel catalysis. *J. Am. Chem. Soc.* **2022**, *144* (4), 1899-1909.

(4) Liu, T.; Zheng, D.; Li, Z.; Wu, J. A Route to O-Aminosulfonates and Sulfonamides through Insertion of Sulfur Dioxide and Hydrogen Atom Transfer. *Adv. Synth. Catal.* **2017**, *359* (15), 2653-2659.

(5) Deeming, A. S.; Russell, C. J.; Willis, M. C. Combining Organometallic Reagents, the Sulfur Dioxide Surrogate DABSO, and Amines: A One-Pot Preparation of Sulfonamides, Amenable to Array Synthesis. *Angew. Chem. Int. Ed.* **2015**, *54* (4), 1168-1171.

(6) Giang Luu, T.; Kim, H. K. Visible-Light-Driven Copper-Catalyzed Synthesis of Sulfonamides from Aryl-N2BF4 through One-pot Tricomponent Reaction. *Adv. Synth. Catal.* **2023**, *365* (10), 1671-1677.

(7) Kariofillis, S. K.; Jiang, S.; Żurański, A. M.; Gandhi, S. S.; Martinez Alvarado, J. I.; Doyle, A. G. Using data science to guide aryl bromide substrate scope analysis in a Ni/photoredox-catalyzed cross-coupling with acetals as alcohol-derived radical sources. *J. Am. Chem. Soc.* **2022**, *144* (2), 1045-1055.

(8) Kirihara, M.; Yamahara, S.; Okada, T.; Matsumuro, H.; Kinoshita, Y.; Kitajima, A.; Takamura, Y.; Odagiri, T.; Asawa, T.; Sugiyama, Y. Synthesis of Sulfonyl Halides from Disulfides or Thiols Using Sodium Hypochlorite Pentahydrate (NaOCl· 5H2O) Crystals. *Synthesis* **2022**, *54* (18), 4120-4128.
